# Rapid Market Screening to assess lead concentrations in consumer products across 25 low- and middle-income countries

**DOI:** 10.1038/s41598-024-59519-0

**Published:** 2024-04-27

**Authors:** Aelita Sargsyan, Emily Nash, Gordon Binkhorst, Jenna E. Forsyth, Barbara Jones, Gabriel Sanchez Ibarra, Sarah Berg, Andrew McCartor, Richard Fuller, Stephan Bose-O’Reilly

**Affiliations:** 1 Pure Earth, 475 Riverside Drive, New York, NY 10115 USA; 2 Doctoral Program in Pollution, Toxicology and Environmental Health, Faculty of Biological Sciences, University of Valencia, c/Dr. Moliner, 50, Burjassot, 46100 Valencia, Spain; 3 Division of Infectious Diseases and Geographic Medicine, Stanford University, Stanford, CA USA; 4 Cardinal Resources, Inc., 4410 Broadway Blvd., Monroeville, PA 15146 USA; 5 Institute and Clinic for Occupational, Social and Environmental Medicine, University Hospital, LMU Munich, Ziemssenstr. 5, 80336 Munich, Germany

**Keywords:** Lead, Metal foodware, Ceramic foodware, Paint, Consumer products, Cosmetics, Spices, Low- and middle-income countries, Ecological epidemiology, Chemical safety

## Abstract

Lead exposure can have serious consequences for health and development. The neurological and behavioral effects of lead are considered irreversible. Young children are particularly vulnerable to lead poisoning. In 2020, Pure Earth and UNICEF estimated that one in three children had elevated blood lead levels above 5 µg/dL. The sources of lead exposure vary around the world and can range from household products, such as spices or foodware, to environmental pollution from nearby industries. The aim of this study was to analyze common products from markets in low- and middle-income countries (LMICs) for their lead content to determine whether they are plausible sources of exposure. In 25 LMICs, the research teams systematically collected consumer products (metal foodware, ceramics, cosmetics, paints, toys, spices and other foods). The items were analyzed on site for detectable lead above 2 ppm using an X-ray fluorescence analyzer. For quality control purposes, a subset of the samples was analyzed in the USA using inductively coupled plasma mass spectrometry. The lead concentrations of the individual product types were compared with established regulatory thresholds. Out of 5007 analyzed products, threshold values (TV) were surpassed in 51% for metal foodware (TV 100 ppm), 45% for ceramics (TV 100 ppm), and 41% for paints (TV 90 ppm). Sources of exposure in LMICs can be diverse, and consumers in LMICs lack adequate protection from preventable sources of lead exposure. Rapid Market Screening is an innovative, simple, and useful tool to identify risky products that could be sources of lead exposure.

## Introduction

Lead is a highly toxic substance especially impacting children’s health^1^. The neurological and behavioral effects of lead are considered to be irreversible^1,2^. Young children are particularly susceptible to lead poisoning. Children absorb up to 4 times better lead compared to adults, their organ systems, especially their cognitive system is still developing and is negatively affected^1^. In 2021, the World Health Organization (WHO) published guidelines on management of lead exposure and recommended that a blood lead level of 5 µg/dL in children should trigger an intervention, although it should be noted that there is no known safe level of lead in blood^3^. In 2020, Pure Earth and UNICEF estimated that one in three children globally suffer from elevated blood lead levels above 5 µg/dL, especially in lower income countries^4^. The Toxic Truth Report highlighted a number of consumer goods that can contribute to lead poisoning, many of which were included in the Rapid Market Screening (RMS) study^4^.

There are multiple lead exposure pathways, from consumption of lead-contaminated dust near industrial hotspots, to ingestion of lead-contaminated paint chips to lead-adulterated spices^2,5–8^. Prior studies have identified lead contamination in a variety of consumer products such as paints, ceramics, spices, foodware, traditional medicines, and cosmetics^1,2,8–13^. However, the geographic variability and overall distribution of lead in these potential exposure sources have not been adequately characterized, particularly in low- and middle-income countries (LMICs). Given that lead exposure sources can vary considerably by location, it is important to identify local sources of lead exposure, especially for young children and particularly within their homes. Local exposure source assessments are important allowing for more accurate interventions that are designed specifically to target local priority sources, allowing for greater effectiveness and efficiency than interventions that are designed without local data.

Therefore, the aim of this study was to systematically analyze products in a range of LMICs for lead. Ultimately, we wanted to determine which product types are more likely to contain lead and how lead concentrations vary around the world. More specifically, the research questions were as follows:

- What is the geographic distribution of lead concentrations among product types identified as containing lead in previous studies in the selected countries?
- How do the lead concentrations in each product type compare to available regulatory standards or health guidelines?

## Methods

The research team developed a “Rapid Market Screening” protocolled by some of the authors (EN, AS, GB, JF), former Pure Earth Senior Director of Programs Petr Sharov and other Pure Earth staff with external expert review prior to implementation (see Supplement A). The protocol describes the information to collect about each market, vendor, and item, and includes standardized analytical approaches for each product type.

### Country selection

The overall goal was to select 25 geographically diverse LMICs, including at least one country from each of the six World Bank-classified regions that represent the majority of LMICs: Africa, Middle East and North Africa, East Asia and the Pacific, South Asia, Europe and Central Asia, and Latin America and the Caribbean. Within each of these regions, candidate countries were listed and weighted for inclusion based on several factors:

- Prioritizing countries with evidence of a high prevalence and/or severity of childhood lead poisoning^14^. For this purpose, an extensive literature review was performed.
- Within each global region, balancing a mix of countries with and without evidence of lead-tainted products (or countries that are hypothesized to sell these products based on cultural similarities).
- Priority was given to countries in which Pure Earth employees or its partners have capacities and in which there is sufficient geopolitical stability to be able to work there safely.

**Figure 1:**
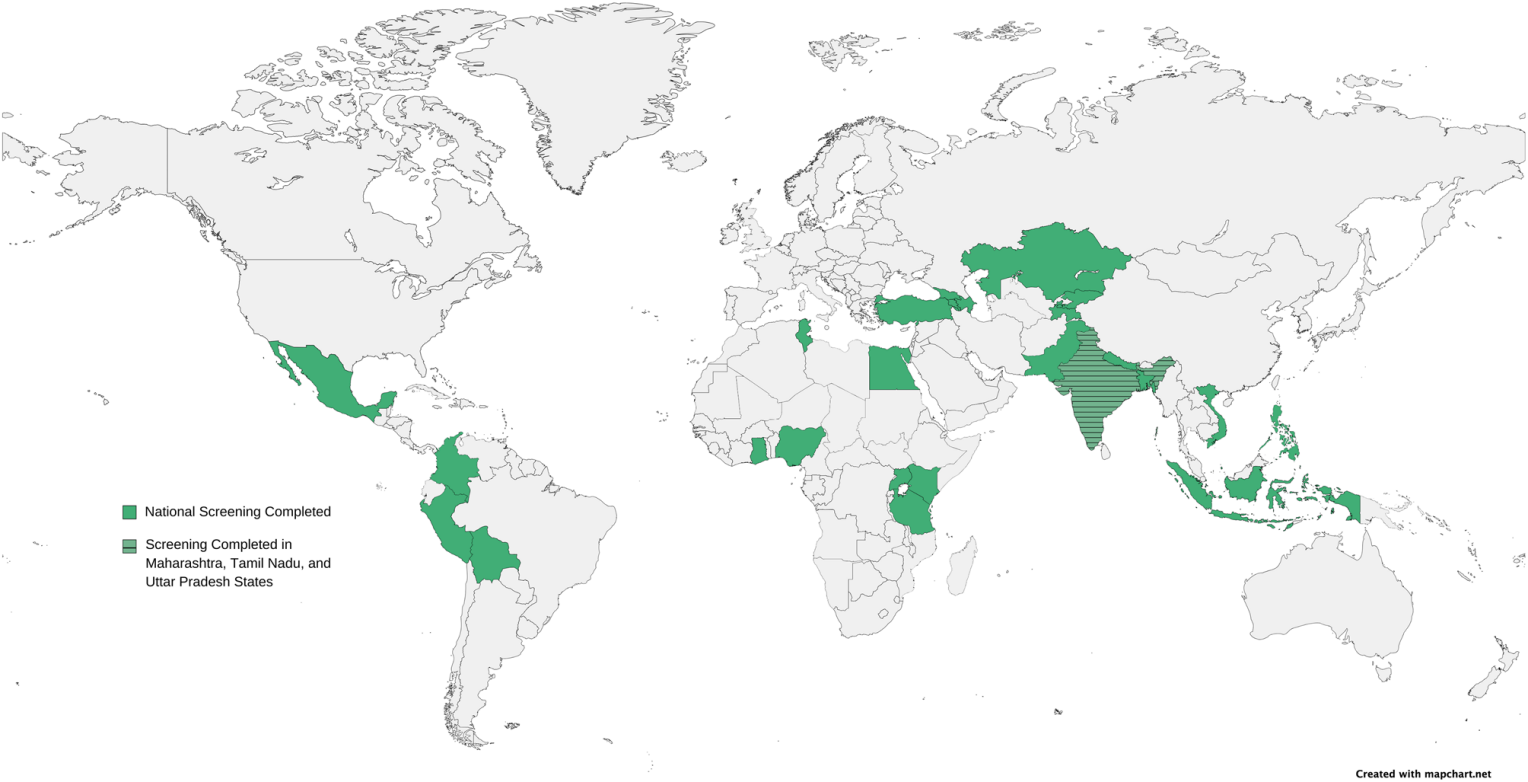
Countries in which the study was conducted.

The countries were evaluated on the basis of the above-mentioned characteristics and ultimately selected by the project team in a discourse. Consequently, a total of 25 countries, including India (Fig. 1), were chosen for the study. However, recognizing the vast size and diversity of India, the sampling strategy focused on three geographically distinct states within the country. As such, the study covered 27 locations, including Armenia; Azerbaijan; Bangladesh; Bolivia; Colombia; Egypt; Georgia; Ghana; the Indian states of Maharashtra, Tamil Nadu, and Uttar Pradesh; Indonesia; Kazakhstan; Kenya; Kyrgyzstan; Mexico; Nepal; Nigeria; Pakistan; Peru; the Philippines; Tajikistan; Tanzania; Tunisia; Turkey; Uganda; and Vietnam).

The cities were samples were selected were:

- Ghana – Cape Coast, Koforidua, Kumasi
- Maharashtra (India) – Kolhapur, Nagpur, Pune
- Uttar Pradesh (India) – Gaziabad, Lucknow, Prayagraj
- Tamil Nadu (India) – Kancheepuram, Namakkal, Tiruvallur
- Indonesia – Makassar, Medan, Surabaya
- Bangladesh – Barisal, Khulna, Rajshahi
- The Philippines – Sebu, Davao, Mabalacat, San Fernando
- Colombia – Barranquilla, Bogota, Bucaramanga, Cali
- Tajikistan – Bokhtar, Dushanbe, Khudjand, Kulyab
- Kyrgyzstan – Bishkek, Cholpon-Ata, Osh
- Kazakhstan – Saryagash, Shymkent, Turkestan
- Georgia – Mestia, Tbilisi, Zugdidi
- Armenia – Gyumri, Vagharshapat, Vanadzor
- Mexico – Cuautia, Huazulco, Puebla, Toluca
- Peru – Callao, Cusco, Lima, Pucallpa
- Tanzania – Arusha, Dar Es Salaam, Mwanza
- Bolivia – Cochabamba, La Paz, Oruro
- Egypt – Alexandria, Cairo, Giza
- Kenya—Kisumu, Mombasa, Nairobi
- Tunisia—Sfax, Tataouin, Tunis
- Nigeria—Abuja, Lagos, Port Harcourt
- Uganda—Kampala, Lira, Mbarara
- Pakistan – Karachi, Lahore, Rawalpindi
- Nepal – Biratnagar, Kathmandu, Neplagunj
- Azerbaijan – Baku, Ganja, Imishli
- Vietnam – Da Nang, Dong Ha, Nam Dinh
- Turkey – Ankara, Bursa, Eskisehir

### Product selection

The general types of products sampled were selected following a series of global desk assessments that reviewed literature on lead concentrations in a variety of products in LMICs^14–17^. An initial list of product types and a sample desk assessment were then provided to the researchers in the countries. The initial list was designed to examine common items across geographies, but also allowed for flexibility based on local contexts. The researchers chose any relevant items for their countries, supporting their point with references (both published and unpublished), and carried out initial product screening. Based on this formative research, eleven product types were selected for analysis: ceramic foodware, metal foodware, plastic foodware, cosmetics, toys, paints intended for large surfaces, paints for art and crafts, spices, sweets, staple dry foods, and traditional and herbal medicines.

### Product sampling

Sample collectors in each country selected in at least three or four geographically diverse cities, and selected, when possible, at least one wholesale bazaar and one retail market in each city to purchase items. Within each market, sample collectors interviewed the sellers and purchased items from at least two vendors. There are different probabilities of lead contamination between whole, unprocessed spices and processed or powdered spices as well as between loose and packaged spices. We opted for the loose processed spices to reduce the likelihood of getting an invalid result due to technical detection issues if there was indeed a problem. Loose processed spices were usually more homogeneous in composition compared to whole or unprocessed spices. The homogeneity reduced the risk of variability and led to more consistent samples. Samples were collected between September 2022 and May 2023.

### Product analysis

The primary screening tools used for the study were portable X-ray fluorescence analyzers (XRF). The portable XRF machine is a valuable screening device for this type of study because it is easy to use, field portable, and gives immediate results that are comparable to laboratory data. Once an XRF device has been purchased, there are few consumable costs, with the exception of ancillary supplies like gloves, bags and labels. The protocol outlined how to prepare each item for testing and specified the number of XRF readings required for each item. In every country except Bangladesh, samples were analyzed with a Thermo Scientific Niton XL3T XRF, using the “Test All” mode, which is designed for consumer goods. In Bangladesh, an Olympus Vanta Series C was used in “Soil” mode, with a subset of those items later also tested with a Thermo Scientific Niton XL3T for confirmation purposes and uniformity of data acquisition. The testers were instructed in the provided protocols and in on-line training sessions to regularly check the accuracy of the XRF using the provided "standard" samples with known lead concentrations. Investigators were instructed to check calibration standards at the beginning of the day, anytime the instrument has been off for 30 min, and at the end of each session. All data collected by the investigators was entered into a central database using the platform SurveyCTO. Subsets of samples from each country were sent to Pure Earth’s Headquarters in New York for quality control (see Supplement B) which consisted of confirmatory testing of 354 representative samples by a certified laboratory and spot checking of field XRF results using the provided protocols and Thermo Scientific Niton XL3T XRF. Local versus New York XRF measurements generally compared well except in the cases of sample heterogeneity (e.g. toys with different colored paints). New York XRF and laboratory data also indicated issues with the field XRF readings from Tajikistan and Kazakhstan, which limited the data from these countries to samples that were sent to New York. Literature shows a XRF and laboratory devices results correlate well in case of soil and sediment samples, however there is not much research done in consumer items^18,19^. For certain product types, especially powdered spices, XRF lead levels correlated strongly with laboratory results, with correlation coefficients (R2) ranging from 0.70 to 0.98 for plastic toys, cosmetics, plastic foodware and spices. For other product types, however, the agreement between XRF values and laboratory results is not well documented and may depend on the form and preparation of the sample. Literature shows that XRF and laboratory devices results do well correlate in case of soil and sediment samples, however there is not much research done in consumer items^20^.

### Statistical methodology

The values were determined using the XRF; they were recorded in Survey CTO, and the statistical analysis was conducted in Microsoft Power BI. Minimum, maximum, quartile and median levels of lead in various products were determined. There was a significant portion of the XRF results below the XRF’s lower limit of detection (LOD). LOD refers to the minimum concentration of an element detectable with reasonable confidence. It signifies whether an element is present or absent, without guaranteeing the accuracy of the obtained value. Typically, the LOD is quantified as three standard deviations (or 1.5 times the reported measurement error) observed in a sample containing either none or only a trace amount of the analyte. For this reason, and since the data was skewed to the left, we do not report mean, but median values. For product types that were expected to be largely homogenous, such as ground spices or paint samples, researchers were requested to take only one reading. For product types with the potential for a high level of heterogeneity, such as toys, ceramics, which could have multiple colors glazes, or metal foodware, which could have components like rivets, researchers were requested to take between 3 and 5 readings. Due to this potential for heterogeneity and the lack of certainty of how each reading would contribute to the exposure profile for that item, the highest of the readings for a given item was used in the analysis. Furthermore, where there are existing regulations for the items included in the study, these regulations apply to all components of an item, rather than an average.

### Reference values

To provide context to the concentrations of lead found in the various products, a “reference level” for each product type was selected. These reference levels serve as thresholds indicating where United Nations (UN) agencies including WHO or particularly well-resourced regulatory authorities have established public health guidance, a level of concern, or a regulatory limit for lead in each product class. Although countries often have unique standards for lead concentrations in different products, a uniform reference level for each product type was applied for this study to facilitate comparisons across countries^21^. The inclusion of uniform reference levels is not a suggestion that any one guidance value or regulatory standard is superior to any other, or that concentrations below the reference levels are safe. Rather, the reference levels used here are simply an attempt to contextualize concentrations and highlight particularly concerning results. We selected existing regulatory standards and guidance values promulgated by UN agencies, the European Union (EU), and the United States (US), prioritized in that order. We could not identify existing reference levels for total lead in foodware (items used to cook, serve, consume, and store food). While standards for leachable lead from foodware exist, field testing of leachable lead in foodware was not possible. We engaged in a substantial effort to test the degree to which lead leaches from metal foodware (mostly aluminum) with a variety of lead concentrations under various cooking scenarios. Results of leachate testing are unpublished as of February 2024, but are being prepared for publication. For this assessment a reference level of 100 ppm of total lead for all types of foodware was applied. This reference level for total lead is not based on an existing regulatory standard, and lead doses per use likely vary between product types of foodware, and indeed between individual products. The lead dose per use is likely also affected by the type of food prepared, the method and duration of cooking, and other contextual factors. In the absence of any available standards for total lead content for these product types of foodware, we used the US Consumer Product Safety Commission total lead standard for “children’s products'' (also used in this study for the product type of toys). The use of 100 ppm total lead as a conservative threshold is supported by leaching tests on over 100 metal foodware items, which indicated that pots with total lead below 100 ppm leached less than the World Health Organization (WHO) drinking water standard of 10 ppb (17 out of 18 pots tested)^22^.

Reference levels and measured lead levels in this study are expressed in parts per million (ppm), which is equivalent to milligrams per kilogram (mg/kg). The following reference values used in this study are listed in Table 1.

**Table 1:** Reference level per product type in ppm.

Product type	Reference level [ppm]	Reference
Ceramic foodware	100	PE (1)
Metal foodware	100	PE (1)
Plastic foodware	100	PE (1)
Cosmetics	2	EU/BFV (2)
Toys	100	US CPSC (3)
Paint	90	UNEP (4)
Spices	2	EU (5)
Sweets	0.1	US FDA (6)
Staple dry foods	0.2	FAO (7)
Herbal/traditional medicines	10	WHO (8)

(1) As explained in the text above, Pure Earth (PE) applied 100 ppm as reference level for foodware; (2) EU regulations state that cosmetics cannot contain heavy metals. They provide exceptions for unavoidable concentrations but do not define these. The German Office of Consumer Protection and Food Safety (BFV) states that for most cosmetics, levels above 2 ppm are avoidable^23^; (3) The 100 ppm limit we used is from the US Consumer Product Safety Commission (US CPSC)^24^. There is a much lower EU standard but it is the amount of lead that can migrate from the toy as opposed to the total lead standard from the US^25^. The EU has a toy standard, but it is a “migration” standard that measures lead leaching from products during an acid bath and is not applicable to XRF measurements of total lead; (4) UNEP “Model Law and Guidance for Regulating Lead Paint”^26^; (5) The EU has several regulatory levels that apply to various spice types , 2 ppm is the highest^27^; (6) US Food and Drug Administration (FDA): Lead in Candy^28^; (7) Food and Agriculture Organization of the United Nations (FAO) – Codex Alimentarius—General Standard for Contaminants and Toxins in Food and Feed^29^; (8) World Health Organization (WHO): WHO guidelines for assessing quality of herbal medicines with reference to contaminants and residues^30^.

## Results

This study analyzed a wide variety of consumer products and materials. Samples included both small batch, informally produced items, as well as large-scale, commercially produced items. There are 382 shopping venues where sampling took place (markets, shopping areas, malls, stores). For metal foodware, total lead levels in 51% of the 520 samples collected were above the reference level of 100 ppm. In 17 locations (countries or, in the case of India, states), even median levels exceeded the reference level and in 9 locations the maximum level exceeded 10,000 ppm (see Supplement D). Of the items in the metal foodware product type that were found to be above the reference level, 69% were pots and pans, 17% were vessels for food or water not intended to be exposed to direct heat, and 14% were cooking utensils. Across all item types, 57% of the items found to be above the reference level were reported to be made of or labeled as aluminum or aluminum alloys. For 35% of the items, we were not able to determine the metal composition based on the item description or label. Other metal types—including brass, copper, and iron alloys—made up the remaining 8% of samples found to be above the reference level. Leachate testing was conducted on more than 100 pots from 25 countries and 5 regions in the US. Lead levels above 100 ppm were common across all regions, indicating the potential to leach lead above the 10 ppb WHO drinking water standard. Notably the 100 ppb threshold could result in a blood lead level of around 0.5 ug/dL based on the US EPA’s IEUBK model, assuming daily intake of 250 g of food at this lead concentration. For ceramic foodware, high lead levels were common across all regions (see Supplement D), with 45% of 308 samples above reference level of 100 ppm. In 11 locations, the median sample exceeded the reference level, suggesting that contaminated items are common. In 25 of the study locations (all but Pakistan and Uttar Pradesh State, India), the maximum lead level was more than 10 times the reference level (see Supplement D). Out of 364 plastic foodware samples, 12% showed lead levels exceeding the reference level of 100 ppm. Unlike ceramic and metal foodware, for which many countries had samples with maximum concentrations above 10,000 ppm, all samples of plastic foodware were below 3300 ppm.

For paints**,** high lead concentrations were prevalent in new paints even among countries that have already adopted a 90 ppm limit (see Supplement D). The paint samples were divided into two product types: paints intended for use on large surfaces, such as interior and exterior walls, and paints intended for crafts, art, and other specialty uses. This division was based on the recognition that exposure pathways may be different between wall paints, where exposure likely results from deteriorating paint that becomes dust, and specialty paints, where exposure may be more directly related to the application of the paint or use of the painted product (e.g. a toddler getting art paints in the mouth or mouthing a painted toy). Note that we were not able to determine the primary purpose of all paint samples collected, and therefore we have included an additional “unclassified” product type category. We also note that the protocol for testing paint was amended during the RMS study to specify testing only dried paint samples as opposed to allowing analysis of wet samples. In total, 41% of the 437 samples of paint for large surfaces were above reference level of 90 ppm. Out of 70 samples of paint for crafts, art, and other specialty uses, 11% showed lead levels exceeding 90 ppm. Among 102 paint samples not classified, 47% showed lead concentrations exceeding 90 ppm. More than half of the study locations (14 of 27) had maximum lead concentrations exceeding 10,000 ppm, while 10 locations had samples exceeding 20,000 ppm (see Supplement D).

For toys**,** high lead levels above the reference level of 100 ppm were found in 13% of 781 samples tested. This product type encompasses a variety of hard toys, composed primarily of plastic items, but also including metal, wood or other materials. Some of these toys were also found to have paint or coatings on them, with lead concentrations either in the paint of the underlying substrate. In addition to the variety at the product type level, many toys were heterogeneous, made from a combination of materials. We found many toys to contain internal electronic or metal parts, which were responsible for some of the highest lead readings observed by XRF. Such readings may not necessarily best reflect the potential exposure risk for that item, as the reference level relates to “accessible parts” to children.

For cosmetics**,** 12% of the 812 samples were above reference level of 2 ppm, across many subcategories. The highest concentrations were reported in traditional eyeliners (see Supplement C). Cosmetics with elevated lead levels were found in 21 of the 25 countries (see Supplement D).

The two items with the highest lead concentration were both eyeliners, known as kajal or kohl, from Pakistan. These samples had concentrations of 637,600 ppm (64%) and one million ppm (100%) lead as assessed by XRF, with lower but still extremely high concentrations (29% and 32%) reported by confirmatory laboratory testing. In some cultures, kajal/kohl is applied to infants and children. The item with the third highest lead concentration of lead (128,400 ppm) was face paint intended specifically for children. Among the samples with elevated lead levels, the most common item was nail polish (29 items, maximum lead concentration of 6,751 ppm), followed by lipstick (15 items, maximum lead concentration of 42,350 ppm), and eyeshadow (13 items, maximum lead concentration of 974 ppm). As noted above and in the Quality Control section (see Supplement B), some deviations were observed between the XRF and lab-based measurements of lead concentration at the highest concentrations among the cosmetics. Nevertheless, at such extreme concentrations, the risk is still significant even with a wide margin of error.

For spices**,** 2% of 1,084 samples were above reference level of 2 ppm. The highest concentrations were found in turmeric and blends (see Supplement C).

Less than 5% of herbal/traditional medicine (4%), sweets (3%) and staple dry food (1%) were above reference levels (for details see Supplement C).

In summary, out of a total of 5,007 product samples from 25 countries, 913 samples had concentrations of lead exceeding the relevant reference level based on XRF readings, representing 18% of all samples. Metal foodware, ceramic foodware, and paints most frequently exceeded the relevant reference levels (see Table 2).

**Table 2:** Distribution of lead concentrations and the distribution of highly lead-tainted samples above the reference level across the 11 product types, aggregated for all 25 countries.

Product type	Total #	Minimum	25th percentile	Median	75th percentile	Maximum	> Reference level [%]
Metal foodware	520	ND	ND	118	754	119,500	51
Ceramic foodware	308	ND	ND	73	3665	397,100	45
Plastic foodware	364	ND	ND	ND	ND	3289	12
Toys	781	ND	ND	ND	13	97,300	13
Cosmetics	812	ND	ND	ND	ND	1,000,000	12
Paints – large surface	437	ND	ND	1	1518	807,309	41
Paint craft/art	70	ND	ND	ND	ND	93,500	11
Paint—unclassified	102	ND	ND	10	3400	79,000	47
Herbal/traditional medicines	54	ND	ND	ND	ND	31	4
Sweets	111	ND	ND	ND	ND	5	3
Spices	1084	ND	ND	ND	ND	622	2
Staple dry food	364	ND	ND	ND	ND	17	1
Total N	5007						

Results in ppm lead. *ND* “non-detect”. The effective limit of detection was in general 2 ppm lead.

**Figure 2:**
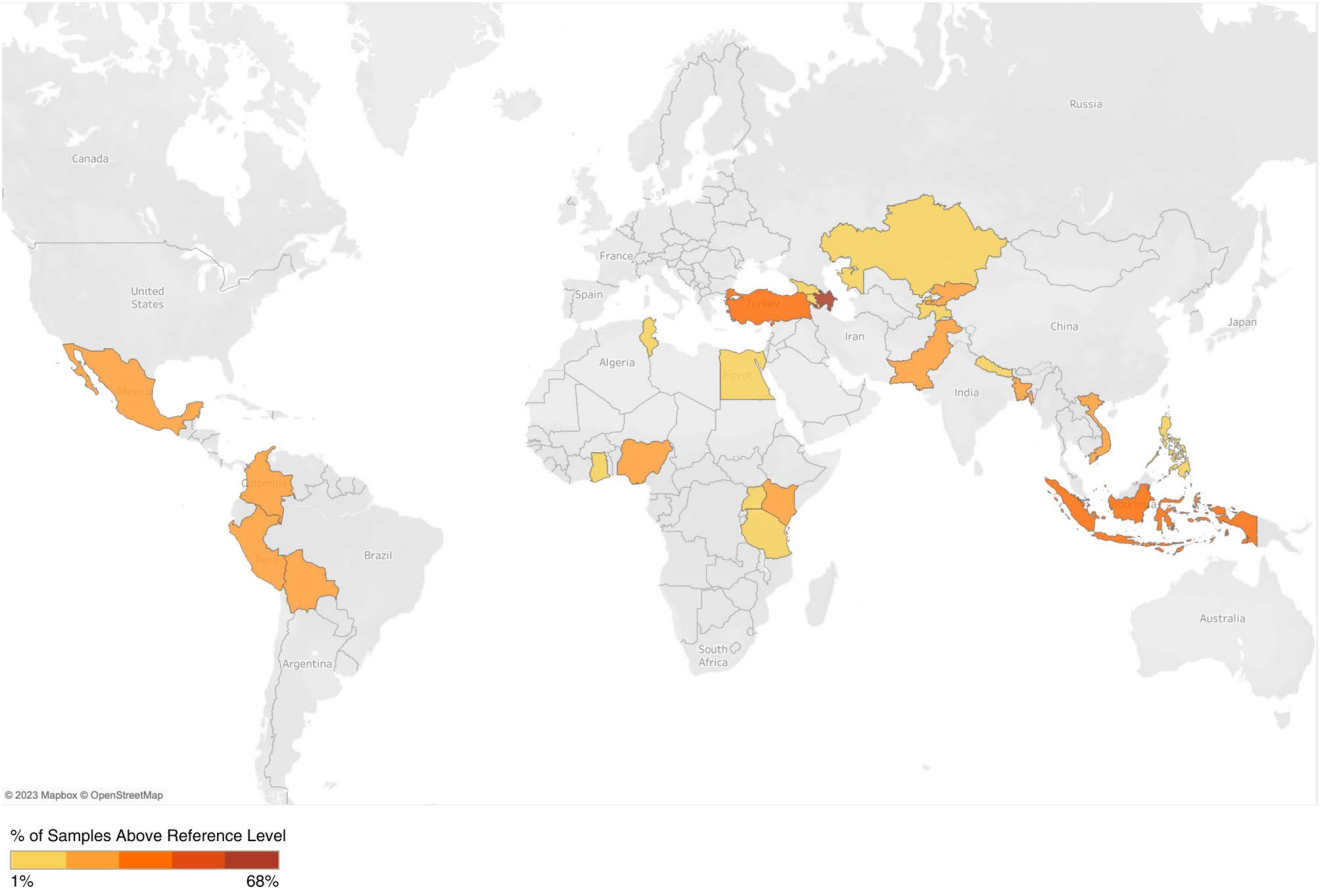
Percentage of samples above reference level by country.

In all 25 countries where this assessment was performed consumer products were identified that exceeded at least some reference levels (see Fig. 2).

The Table 3 is organized by country and shows percentages of samples of each product and food product type that exceeded the relevant reference level.

**Table 3:** Percent of samples exceeding reference level per product type and by country.

Region	Country	Ceramic foodware	Metallic foodware	Plastic foodware	Cosmetics	Toys	Paint—large surfaces	Paints—crafts/art	Spices	Sweets	Staple/dry foods	Herbal/trad medicine
Caucasus	Armenia	36%	11%	6%	7%	3%	0%	0%*	4%	NA	11%	NA
Caucasus	Azerbaijan	100%	63%	60%*	10%	69%	100%	NA	0%*	NA	NA	NA
Caucasus	Georgia	48%	16%	0%*	0%	3%	50%*	7%	0%	NA	0%*	NA
C. Asia	Kazakhstan	NA	NA	NA	0%*	33%*	NA	NA	0%	NA	0%*	NA
C. Asia	Kyrgyzstan	44%	19%	13%	15%	6%	33%	NA	0%	NA	0%	NA
C. Asia	Tajikistan	100%*	NA	NA	0%*	0%*	NA	NA	60%*	NA	0%*	NA
S.S. Africa	Ghana	18%	55%	0%	7%	14%	0%*	0%*	0%	NA	0%	NA
S.S. Africa	Kenya	62%	53%	25%	6%	3%	36%	NA	0%	NA	0%	NA
S.S. Africa	Nigeria	29%	66%	4%	18%	16%	76%	NA	0%	NA	0%	NA
S.S. Africa	Tanzania	67%*	35%	4%	3%	10%	7%	NA	2%	3%	0%	NA
S.S. Africa	Uganda	8%	73%	20%	2%	0%	16%	NA	0%	NA	6%	100%*
L. America	Bolivia	60%	54%	14%	46%	6%	0%*	NA	0%	0%*	0%	NA
L. America	Colombia	50%	40%	24%	10%	12%	31%	11%	2%	0%	0%	0%
L. America	Mexico	67%	25%	8%	7%	22%	93%	NA	3%	4%	0%	0%*
L. America	Peru	42%	69%	17%	9%	2%	10%	0%	2%	NA	0%	0%*
MENA	Egypt	50%	55%	13%	42%	4%	0%*	NA	2%	NA	0%*	0%
MENA	Tunisia	56%	12%	4%	11%	4%	50%	NA	0%	NA	0%	17%
MENA	Turkey	53%	67%	19%	100%*	29%	70%	NA	25%*	NA	NA	NA
S. Asia	Bangladesh	44%	59%	9%	6%	13%	0%*	50%*	7%	NA	17%	NA
S. Asia	Maharashtra, lndia	71%	63%	19%	3%	21%	19%	17%	0%	NA	0%	0%*
S. Asia	Tamil Nadu, India	50%	70%	14%	9%	23%	57%	NA	0%	NA	0%	NA
S. Asia	Uttar Pradesh, India	0%	65%	0%	2%	24%	42%	NA	12%	NA	0%*	0%*
S. Asia	Nepal	9%	100%	12%	0%	0%	0%	NA	0%	NA	0%	0%
S. Asia	Pakistan	20%*	75%	8%	30%	13%	35%	NA	9%	0%	0%	NA
SE Asia	Indonesia	NA	60%	NA	33%	10%	97%	NA	0%	NA	0%	NA
SE Asia	Philippines	13%	24%	0%	13%	6%	16%	0%*	0%	NA	2%	0%
SE Asia	Vietnam	29%	56%	0%	23%	7%	59%	50%	3%	NA	0%	0%*

*Results from 5 or fewer samples.

In Table 4 the regional variation of the results is shown. There are regional trends detectable.

**Table 4:**
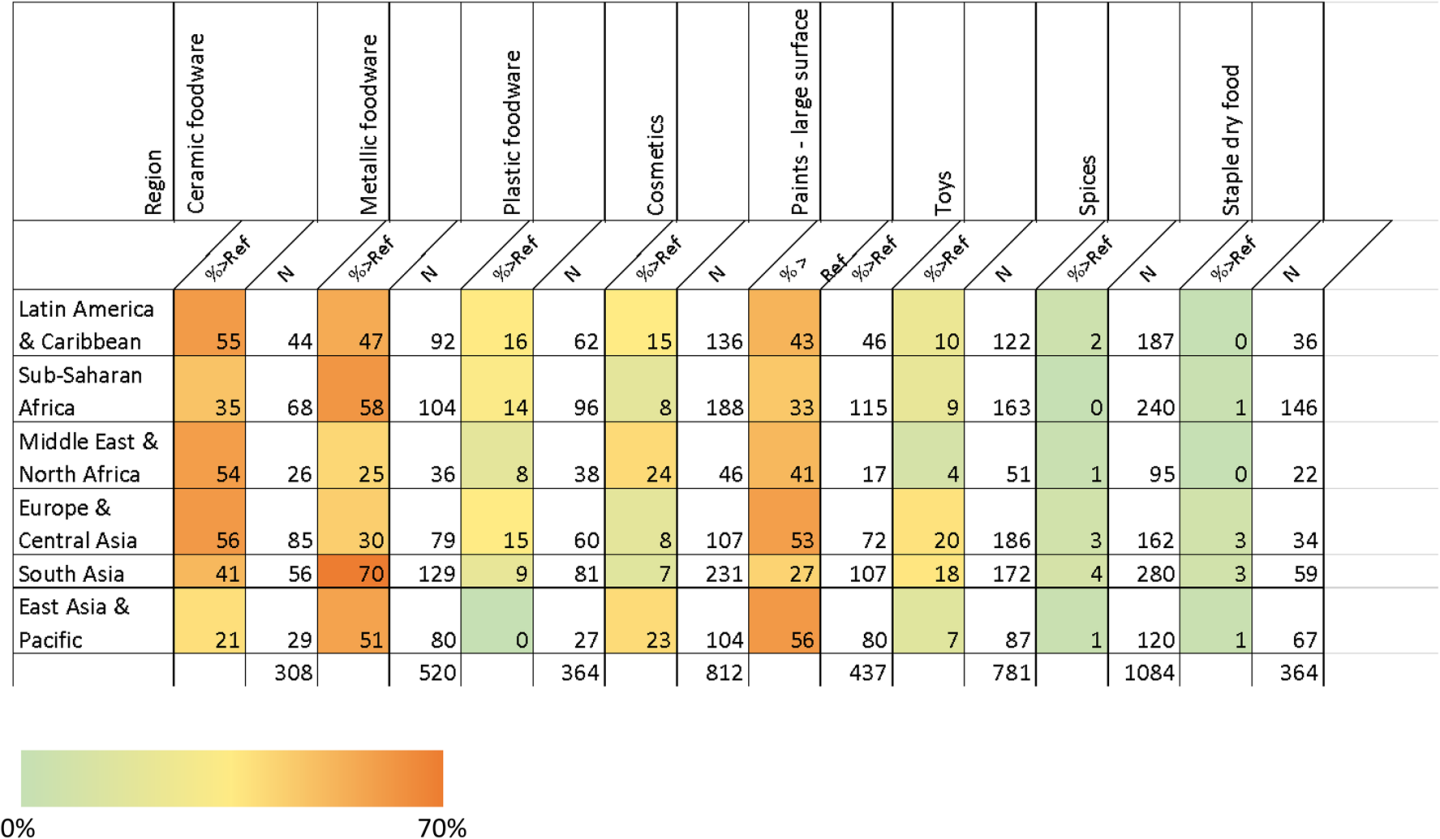
Distribution of lead concentrations and the distribution of highly lead-tainted samples above the reference level analyzed by region.

Results in ppm lead. Only product types with sufficient numbers of samples shown.

## Discussion

Previous studies have highlighted elevated levels of total and leachable lead in metal foodware made in LMICs, particularly in inexpensive aluminum foodware^11,12,31,32^. These pots are generally light, inexpensive, and have good conductivity, which helps conserve fuel usage. Such foodware has previously been found to be made from mixed scrap metal from engine parts, radiators, electronic appliances, and aluminum cans^31^. In all countries, total lead levels were above the reference level of 100 ppm; in South East Asia 70% of the samples were above this level (see Table 4).

The leachable lead from these pots represents an exposure source through ingested food cooked in these pots. Pure Earth has conducted leachability testing of more than 100 aluminum foodware samples to improve our understanding of the allocation of leachable lead in LMICs and potential doses of lead per use. The results of the leachate investigations in aluminum foodware are currently being prepared for publication. Of the 102 pots tested, 45% had lead concentrations in the leachate exceeding 10 µg/L which could result in a blood lead level in children above 0.47 μg/dL based on modeling conducted using IEUBK. The highest leachate concentration found was 2900 μg/L in a pot from Indonesia, a concentration that could result in a BLL of about 45 μg/dL. The pots with the highest leachate concentrations were from South and Southeast Asia. We found a high frequency (45%) and relatively wide geographic distribution of lead in ceramic foodware (see Table 4). Challenges regarding the use of lead-based glazes have been well-documented in Mexico^9,33,34^, and have been identified elsewhere^35^, but the RMS study shows a fairly uniform geographic distribution of contaminated items. This does not necessarily mean that these pieces all contribute to exposure equally. As with all forms of foodware, a high lead concentration on exterior surfaces does not tell us how much lead is leaching into food. The type of glaze, temperature in which it is fired, types of food prepared or served, and ways in which the item is used can all affect leachability and thus exposure^36^. Ceramics with high concentrations of lead were not limited to handmade, artisanal, or traditional pieces, but included mass-produced pieces that may have been imported to the country where they were purchased. The leachability of lead from various ceramic glazes produced and used under different conditions is an area that requires further research that was beyond the scope of this study.

As of January 2024, 48% of countries in the world had legally binding controls on lead concentrations in new paints^37^. Many of these have adopted regulations based on a model law establishing a maximum lead concentration of 90 ppm^26^. However, many of the paint samples analyzed through the RMS study that exceeded 90 ppm were collected from countries that have a 90 ppm regulatory limit. In eight of the countries (Colombia, Georgia, Kenya, Kyrgyzstan, Mexico, Pakistan, Philippines, Vietnam) and Indian States (Maharashtra, Tamil Nadu Uttar Pradesh) were such regulations apply, more than 10% of paint samples had lead concentrations above 90 ppm (details see Supplement C). This suggests a considerable enforcement gap in these locations.

In cosmetics**,** in addition to high lead levels in kajal/kohl, elevated lead levels were found in other traditional products, including henna and kumkum (a red powder made of turmeric and other ingredients and used for social and religious purposes in India)^38^. Lead levels above the reference level were also found in a variety of conventional cosmetics, such as nail polish, lipstick, and eyeshadow as previously described, as well as face powder, mascara, eyeliner, liquid foundation/concealer, and hair products^23^.

Previous studies have identified elevated lead levels in certain spices from countries around the Mediterranean, the Caucasus, and South Asia, among other^39–43^. In several countries, prior programs have confirmed that elevated lead concentrations were the result of producers adding lead-based pigments to spices to make their colors brighter^8^. The RMS study was not designed to focus specifically on countries known to have contaminated spices, nor to focus solely on the types of spices that have been identified as more often contaminated. Rather, the RMS study includes a broad range of spice types from countries that were selected based on product-agnostic criteria. As a result, the findings generally show low levels of lead in spices. The minimum detection level for the XRF is often between 2 to 4 ppm for spices, and thus it is possible that some samples had a reading of “non-detect” but exceeded the reference level of 2 ppm.

To our knowledge, this screening effort encompasses sampling from the widest range of consumer product types and geographies to date. Still, there are limitations in sample size for any given product type due to budgetary and logistical constraints. In some instances, there are only a few samples of a certain product type for a specific country. This study includes samples from 25 countries, which were selected at convenience. From each country, mainly three or four geographically diverse major cities were selected. The authors do not purport that these findings can be considered definitive for a country or region or globally, but they provide indications of potential sources of concern and hotspots geographically where supply chains do not effectively regulate for lead contamination.

## Conclusion

This screening effort revealed elevated lead levels among multiple product types and across diverse geographies. Total lead in metal foodware exceeded the 100 ppm reference value in more than half of the metal foodware tested, with samples above the reference level in all 25 countries in this study. The situation is similar with total lead content in ceramic foodware. In both cases, it is the amount of lead leaching out of these items into food that represents a potential exposure source. XRF analysis of foodware for total lead is relatively cheap and simple, especially compared to heated leachate testing and associated laboratory costs for analysis with limited resources in LMICs. Further studies on leachability—especially of metal and ceramic foodware—are required to establish usable total lead reference values. A transfer factor for lead from foodware to food would allow more LMICs to better protect consumers from lead exposure from foodware.

The problem of lead paint is very well known, but as our results show existing national and international measures to eliminate lead in paint are not sufficient. In this study, we bought new paints on the markets 41% of which contained lead above the threshold value of 90 ppm. This means that lead-based paints are still being sold and used today to paint large surfaces in homes, exposing more generations to lead in the air and dust that is harmful to the development of the nervous system. Better lead paint laws need to be enacted and/or regulations enforced. There is also a need to analyze the supply chains for cosmetics to determine where these high lead cosmetics come from and how they are traded to end up in local markets.

With so many consumer products in 25 countries containing significant amounts of lead, there is a clear global public health impact as lead from these products exposes pregnant women, infants, children, adolescents and adults. Few LMICs conduct large surveys or ongoing monitoring of children’s blood lead levels. The result is that there is little visibility into the prevalence, severity, and geographic distribution of lead poisoning for many countries. This study highlights the allocation of potential lead exposure sources and the importance of blood lead level surveillance and related source apportionment to prioritize product- and non-product lead exposure sources and their elimination in low- and middle income-countries.

### Supplementary Information

Supplementary Information 1.

Supplementary Information 2.

## Data Availability

The RMS dataset supporting the conclusions of this article is) available in the Zenodo repository under 10.5281/zenodo.10444602 and https://zenodo.org/records/10444602.

## Abbreviations

AAS: Atomic absorption spectrometry
CDC: Center of disease control
EU: European Union
FAO: Food and Agriculture Organization of the United Nations
ICP-MS: Inductively coupled plasma mass spectrometry
LMIC: Low- and middle-income country
LOD: Limit of detection
ND: “Non-detect”
PB: Lead
PE: Pure earth
ppb: Parts per billion
ppm: Parts per million
RMS: Rapid market screening
WHO: World Health Organization
XRF: X-ray fluorescence
UN: United Nations
UNEP: United Nations Environment Programme
US: US Consumer Product Safety Commission
