# Supplementary material for: Rapid Market Screening to assess lead concentrations in consumer products across 25 low- and middle-income countries
Source: Sci Rep. 2024 Apr 27;14:9713. doi: 10.1038/s41598-024-59519-0 (PMC11055946; doi:10.1038/s41598-024-59519-0)
Supplement: Supplementary file 1 — Supplementary Information 1. [file 41598_2024_59519_MOESM1_ESM.pdf]

PURE EARTH

Rapid Market Screening Program

Annex A

RMS Final Protocol

Table of Contents

Market Selection and Sampling Approach 3
Information and Sample Collection 3
IN THE FIELD 4
A. Market-level questions 4
B. Vendor-level questions 5
C. Item sample-level questions 6
AT HOME 8
IMPORTANT NOTES 8
Specific Sampling Guidance for Different Products 9
Spices 9
Ceramics/pottery 9
Metal cookware 10
Plastic kitchen items 11
Traditional medicines 11
Cosmetics, religious powders, and personal care products 12
Paints 12
Toys 13
Food items 14
Other 14
Appendix A: Number of items to be sampled from each of 3 cities 14
Appendix B: Number code for each item type 16
Appendix C: Number code for each country 16
Appendix D: Guidelines for XRF Use 18
Appendix E: Sample XRF Data Collection Table 23

Market Selection and Sampling Approach

RMS Phase 2 sampling must be done in three large cities in each selected country or Indian state. The sampled items will be tested with a portable XRF, and data uploaded into SurveyCTO. Selected items will be shipped to the New York for lab analysis.

1. Identify at least two markets in each city - one major wholesale bazaar and one major retail market. Please speak to several sources to identify the wholesale bazaar. Do not automatically pick the one closest to you. Ideally, we are looking for the bazaar that supplies the city, or at least a major portion of it. The second market can be any big retail market from which small businesses and retail consumers purchase items. Both markets should have multiple varieties of items of interest available.
2. Upon arrival at the market, take a picture of the entrance. Ask around for vendors of the items of interest. Identify vendors who are set up sufficiently apart from each other. This is for two reasons: to procure distinct samples from the same market, and to avoid interference or suspicion of one vendor while speaking with another. Ensure that the vendors have sufficient time and are generally cooperative. You will need to spend at least 20 minutes speaking with each vendor. **You will speak to at least two vendors at each market, making it a minimum of four rounds of items and data collection**, so plan well and pace yourself accordingly.

The numbers of samples of each item type to be sampled is presented in the Quick guide. Please focus on the most commonly used brands of the items of interest.

Once you have identified the markets or after an early familiarization visit, note down the following information:

General information (observe, don't need to ask)

1. Country
2. City
3. Item of interest to be sampled (from list presented in Appendix A)
4. Market type – Wholesale, Retail, or Wholesale & Retail
5. Total number of vendors of the item of interest (estimate)
6. Vendor number (you define this code based on the order visited, e.g., 01, 02, 03…10, 11…)

Information and Sample Collection

1. Introducing yourself. It is best to identify yourself and strike up a rapport with the vendor to avoid suspicion.

You can introduce yourself as a researcher and explain that you're primarily interested in understanding the different types, prices, quality, and potential health risks of items available. You can adapt your approach given the local context.

You can assure the vendors that any specific information about them or their stall will be kept confidential. Only the city and market name will be made public.

2**. Identifying types of the item of interest.** Where applicable, start by asking what different types of items of interest the vendor sells. Types may vary by geographical location of manufacture or import, or other factors (such as batch or production year). **If both vendors have the same types of items, collect from both but try your best to get a varied sample.**

3. Gathering information. Consider memorizing the main aspects of the questionnaire so that you can have a natural conversation with the vendors. Based on your conversation with the vendor, start noting down important information to later fill into the online SurveyCTO form (see the “AT HOME” section below). Avoid using electronic devices (phones, tablets) to note the information while at the market as they tend to attract attention. Use a notebook and pen, and fill out digitally later. If permissible (and not too noisy), ask to record (audio only) the person for supplementary information that may help you later. However, please have your phone or other device ready for snapping quick photos, as described below.

The questionnaire can be divided into three sections:

A. General and Market-level (for each market)
B. Vendor-level (for each vendor)
C. Sample-level (for each sample)

Questions marked with an asterisk (*) are mandatory.

IN THE FIELD

A. Market-level questions

A1*. Sampling date

A2*. Sample collector (your) name

A3*. Country (or state) name

A4*. City name

A5*. Market name

A6*. GPS data of the market (enter the longitude and the latitude of the market)

A7*. Market type (wholesale, retail, retail supplied by adjacent wholesale market or other (write in))

A8*. Estimated number of vendors of the item of interest in marketplace

Pictures:
Take a picture of the entrance to the market. Prior to uploading, name the picture file: *"country code (see below) market name.jpg"*

B. Vendor-level questions

B1*. Vendor number for samples (define this code based on the order, e.g., 01, 02, 03…10, 11)

B2. Vendor contact information (if provided, e.g., vendor name, store name, phone number from a receipt)

B3*. Types of items for sale by vendor (select all that apply)

B4* Customers: Who are the vendor's main customers? (You can select more than one option)

Pictures: Take a picture of the storefront (if the vendor allows). It is not important to get the vendor in the picture. Use this picture, below, from a wholesale vendor in Bangladesh as a reference.

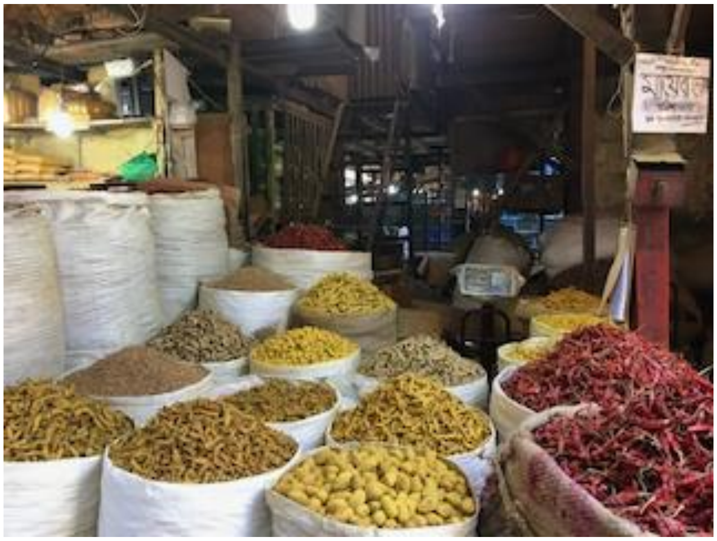

For your picture of the vendor's storefront, please re-name the file as follows: *"country name city name ITEM type.jpg"* (the "ITEM type" is a number, e.g. "1" for spices)

C. Item sample-level questions

Ask the vendor about and take a sample of EACH item of interest that is for sale. Note that the goal is to gain supply chain information about each item of interest to help gain an understanding of the potential source(s) of lead impacts to these items. For example, to understand whether all of the lead impacted toys are imported from a particular country or manufacturer.

For each sample, record the following information:

C1* How many total samples of all items were purchased from this vendor?
After you enter the number, the questions about the samples will be repeated this same number of times, giving you the opportunity to enter data about every sample.

C1.1* Item of interest

C1.2* Please specify the exact item of interest
Examples:

- Spice: what exact spice? Turmeric, chili powder, etc.
- Paint: type of paint, color
- Cosmetic: what type of cosmetic? Lip stick, eye shadow, nail polish, etc.

C1.2.1 The **3-digit number** of the sample (Example: 003; 045; 256)

Please assign the samples consecutive numbers. If 2 teams are collecting data, one of the teams can use even numbers while the other team uses odd numbers, so that there is no duplication of numbers.

C1.3* Manufacturer and brand. If there is no brand, write “unbranded.” The question on “brand” will appear when product type is chosen (e.g., “metal cookware”).

C1.4* Sale price in USD per gram/per kg or per item (if price format is different, enter best guess)

C1.5 Production location (country/region) “country/region of origin.” This may be within the country or imported from abroad. For spices, indicate harvest location AND polishing/processing location if known.

C.1.6 Provide any available information on the supplier of this item (i.e. who the vendor gets it from)

Label each item or sample bag with the following sample ID. **Be very careful while labelling your samples so as not to mix them at the market.** Do not underestimate this step since you will end up handling many different samples by the end of the process. Be systematic and carefully follow protocol for packing and labelling.

Pictures:

Take a picture of each bagged and ID-labeled sample. Upload the photo of the labeled sample. **Prior to uploading, the name of the image file should be changed from what the camera assigned to the sample ID.

SAMPLE ID: Unique ID code for every sample:

- number code for country
- 3 letters of city name
- 3 digits for sample number

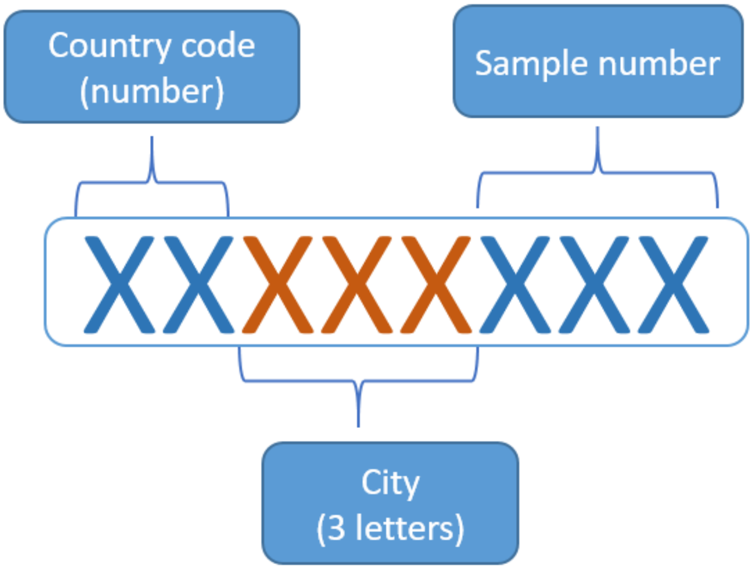

C1.7* Sample ID

The sample ID will be automatically provided by SurveyCTO during data entry. Just move to the next question.

C 1.8* How did you measure the concentration of lead (Pb) in the sample? (via XRF, or in the laboratory, or both)

C 1.9* How many measurements of the sample were taken via XRF? (record number of readings taken of the same item, if applicable))

After you enter the number (up to 5) of measurements taken, the questions about the XRF measurements will be repeated this same number of times, giving you the opportunity to enter data about every measurement.

C 1.9.1* Concentration of lead (Pb) via XRF – record multiple readings (up to 5) of the same item (if applicable) in ppm as well as XRF mode (use “Test all” mode.)

After entering the XRF measurement data, you are encouraged to enter additional notes, observations, or information about each sample or item. This could include physical description, use of item (e.g., decorative, edible, cooking), any labels or warnings on the products, etc.

C 1.10 Concentration of lead (Pb) via laboratory testing

Please enter the concentration of lead, unit of measurement, and method of analysis used in the lab.

Enter any additional notes about each sample or item. This could include physical description, any labels or warnings on the products, etc.. The details on what information to record is provided in the specific guidance for each item category, below.

AT THE OFFICE/HOME

Samples are purchased in the field. They are prepped and analyzed at the office/home, which is also where data entry is completed.

Fill out one form for each vendor.

The form includes both multiple choice and open-ended questions. For open-ended questions, please write as much detail as possible.

If you select “other” as a response, please add additional details.

At the end of the form, there is a “notes” section where you can add any more thoughts or details.

IMPORTANT NOTES

Please retain all samples. A subset will be selected to be mailed to Pure Earth HQ for further analysis.

Careful data collection and recording is critical to this project. To ensure data is collected in a consistent way, please follow the XRF guidance carefully and record XRF readings in the format in Appendix E.

Specific Sampling Guidance for Different Products

This section details how to take measurements for each main type of item. Before operating the XRF, fully review Appendix D, which provides important information on operating the XRF safely and ensuring high quality data.

Careful data collection and recording is critical to this project. To ensure data is collected in a consistent way, please follow the XRF guidance carefully and record XRF readings in the format in Appendix E. In addition, XRF data must be downloaded from the analyzer in Excel format for reference and documentation purposes. Tables with handwritten entries (as in Appendix E) and Excel file(s) for each date of screening must be provided electronically to Pure Earth HQ.

As noted in Appendix D, at the beginning of the day or anytime the instrument has been off for 30 minutes, conduct a calibration verification. Select the appropriate standard for the sample type or matrix you will be analyzing. This data should be recorded on the form provided in Appendix E for documentation.

Spices

- Each sample should be about 100 to 200 grams.
- Spices may be available in a range of forms - loose powder, packaged powder (branded or no brand), and other.
- Each unique form counts as one type - polished finger roots grown in Andhra and polished in Kerala would be considered a different type than powder from those same polished finger roots. Note that sometimes powder can be a mixture of different root types.
- Collect samples of different production or harvest year, if available.
- Take samples of EACH of the different kinds of spices EXCEPT packaged branded powder.
- Analyze samples with the XRF in *Test All* mode.
- Spices must be in thin clear plastic bag and well-mixed/uniform. Sample should be at least 1cm thick when shot with the XRF.
- Each sample must be shot at least 1 time as samples should be homogeneous (okay to evaluate this with more than one reading).
- Reference level: 0.6-2 ppm depending on the spice (EU)

Ceramics/pottery

- Lead has been found in glazes used on ceramics and pottery (particularly those fired at low temperatures). Prioritize items that will come into direct contact with food, especially in the presence of heat or acid, such as cookware or cups/mugs.
- Use the *Test All* mode for analysis.

- Each item should be shot at least 3 times, prioritizing measurements in the inside of the item that is in contact with food/drink.
- **NOTE**: An XRF is a screening tool for total metals only. Leaching tests must be completed to evaluate potential lead exposure through food or liquids. Keep ceramic samples for possible future leachate testing.

Metal cookware

- Lead has been identified in cookware items, particularly inexpensive, locally or informally manufactured aluminum cookware.
- Some examples include pots, pans, woks, pressure cookers, stewpots, Dutch ovens.
- The directions below apply to any metal cookware, including aluminum or other metals. Ceramic cookware is covered above. Plastic kitchen items are covered below.

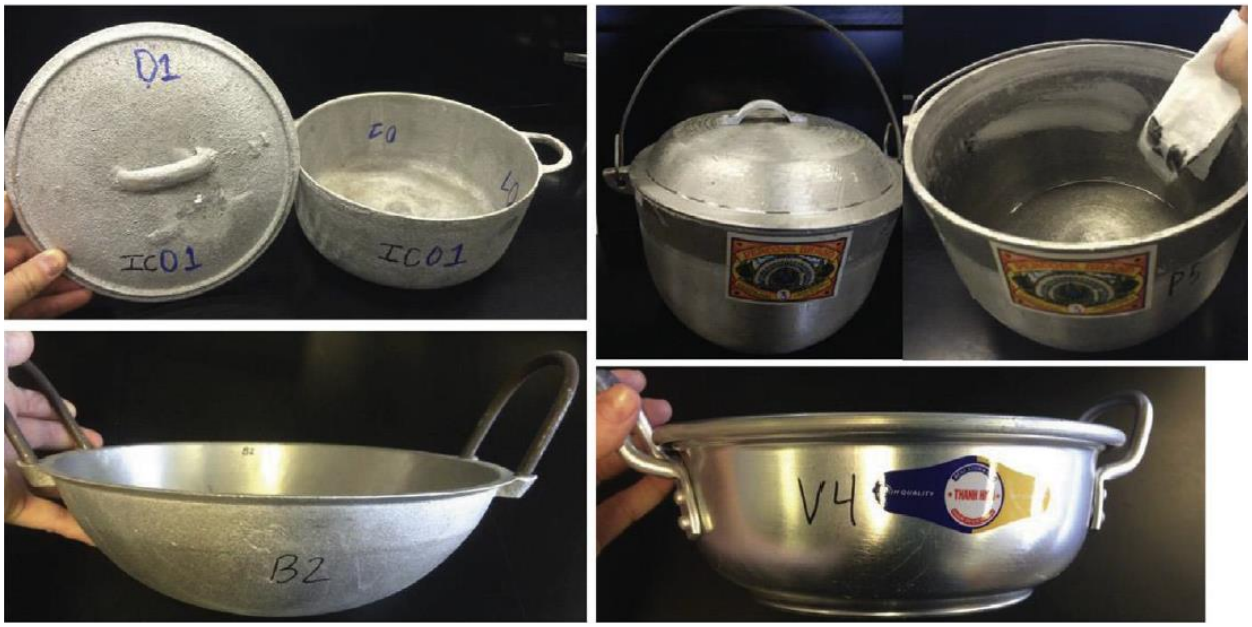

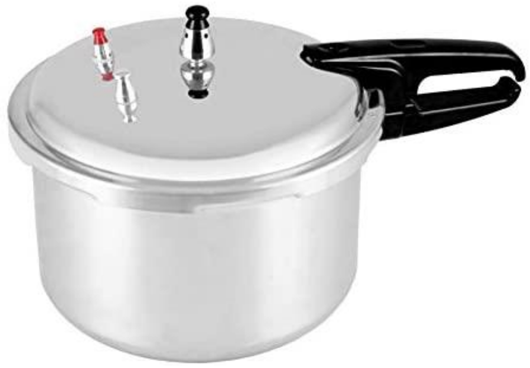

Photo credit: Weidenhamer et al 2017[1]; https://untilgone.com/products/aluminum-pressure-cooker.html#product-accordion

- Record the type of cookware - the local term can be used. Record any information on what material the pot is made of. Record if there is a coating on the pot, such as non-stick.
- Take at least 5 XRF readings on each item in *Test All* mode. If possible, take readings on the inside of the item (the cooking surface). Include readings of any rivets (e.g., for handles) or fixtures on tops (e.g., valves for pressure cookers). Record what part of the item each reading corresponds to. See attached example table in Appendix E). If in doubt, please discuss with HQ to evaluate best options.
- **NOTE**: Using an XRF is a screening tool for total metals only. Leaching tests must be completed to evaluate potential lead exposure through food or liquids.

Plastic kitchen items

- This category includes plastic items that are used for food consumption and storage, particularly bowls and cups where there is a long-contact time with the food. Prioritize items used by children.
- Use the *Test All* mode on the XRF (1 reading).

Traditional medicines

- This category includes folk or herbal remedies, ayurvedic or other traditional medicine.
- Medicines in loose or tablet form can be put in a plastic bag.

[1] Weidenhamer JD, Fitzpatrick MP, Biro AM, Kobunski PA, Hudson MR, Corbin RW, Gottesfeld P. Metal exposures from aluminum cookware: An unrecognized public health risk in developing countries. Sci Total Environ. 2017 Feb 1;579:805-813. doi: 10.1016/j.scitotenv.2016.11.023. Epub 2016 Nov 18. PMID: 27866735.

- Tablets – tablets or other forms should be ground and homogenized for consistent XRF readings.[2]
    - Prepare by using a mortar and pestle (or equivalent) to crush whole tablets placed inside a plastic bag to prevent cross contamination, and secured within a piece of folded card stock.[3]
- Capsules should be emptied into a plastic bag, wearing clean gloves.
- The samples should be well mixed/uniform and should be at least 1cm thick when shot with the XRF.
- Record the advertised composition and use of the product.
- Collect XRF measurements in *Test All* mode (1 reading).
- Reference level: 10 ppm (WHO)

Cosmetics, religious powders, and personal care products

- Dry powders – treat like spices. Remove from the original container and put into a plastic bag before analyzing. For example, blush or eyeshadow may be sold pressed in a metal tray – the powder should be removed from this tray and put into a plastic bag before testing.
- Lipsticks/lotions/creams – you do not need to dry the product. It can be placed in a plastic bag before testing.
- Where possible, samples should be at least 1cm thick.
- Nail polish – you will create a sample of dried nail polish (a "swatch"). Label the swatch with the same sample ID you assigned to the container of nail polish when it was purchased. Apply at least 3 thick layers of the polish to white poster board, card stock, cardboard, laminated material, wooden stick, or a Lenata paint chart. Allow to dry completely. Be sure to collect a "blank reading" of the material you are painting to ensure there is no detectable lead in the background. The swatch should be at least $1cm^2$ in area.
- The samples should be analyzed in *Test All* mode. (1 reading)
- Reference level: 2 ppm (EU/Germany)

Paints

- Mostly solvent-based paints should be purchased. Where solvent- or oil-based (enamel, alkyd, etc.) paints are not available, water-based (emulsion) paints may be purchased.

---

[2] Reames G, Charlton V. Lead Detection in Food, Medicinal, and Ceremonial Items Using a Portable X-Ray Fluorescence (XRF) Instrument. Journal of environmental health. 2013;75(6):16-21.
[3] Kathryn G. McIntosh, Diana Guimarães, Matthew J. Cusack, Alexei Vershinin, Z.W. Chen, Karl Yang & Patrick J. Parsons (2016) Evaluation of portable XRF instrumentation for assessing potential environmental exposure to toxic elements, International Journal of Environmental Analytical Chemistry, 96:1, 15-37.

- We are most interested in paints that individuals, especially children, are likely to encounter through their homes, toys, or playgrounds. It is possible that industrial paints may be sold and used for such purposes so it is important to observe what types are readily available to consumers.
- Record information on the paint sampled.
  - Indicate the primary use of the paint:
    - Intended for large surfaces – such as that which would be used on interior or exterior walls **(ITEM CATEGORY = 9)**
    - Art or craft paint – including children's paint and specialty paints **(ITEM CATEGORY = 13)**
    - Unknown or other (**ITEM CATEGORY = 15**)
  - If possible, record the type of paint:
    - Solvent-based
    - Oil-based (enamel, alkyd, etc.)
    - Water-based (emulsion)
  - Record any additional relevant labeling on the can (for example, "lead free" label).
- Focus on brightly colored paints – particularly yellow, orange, red.
- You will create a sample of dried paint (a "swatch"). Label the swatch with the same sample ID you assigned to the container of paint when it was purchased. Apply 3 thick layers of the paint to white poster board, card stock, cardboard, laminated material, wooden craft stick (at least 1 cm wide), or a Lenata paint chart. Allow to dry completely. Be sure to collect a "blank reading" of the material you are painting to ensure there is no detectable lead in the background.
- Measure dried paint in **PLASTICS mode** (with a reading in ppm). Note that readings in PAINT mode with return reading in mg/cm2 which cannot be compared to international paint standards.
- If you encounter paint pigment in powder form, this can be analyzed with the XRF by shooting through a thin plastic bag (like other powders described above). For pigments, the **ITEM CATEGORY = 14.**
- Please retain paint samples for possible additional testing, although laboratory testing is not envisioned at this time.
- Reference level: 90 ppm (UNEP)

Toys

- Record the characteristics of the toy – plastic, wood, metal, whether it is painted.
- Use the *Test All* mode on the XRF. This mode can be used regardless of the material the toy is made out of, and whether it is painted or not.
- Record the specific component of the toy that you measure with the XRF – the part of the toy, the color, the material, etc.
- Make sure there are no metallic parts (wires, screws, batteries, gear boxes) inside toys that may impact XRF reading.
- Please retain toys for possible additional testing.

- Reference level: 100 ppm or 90 ppm in paint or any coatings (US Consumer Product Safety Commission)

Food items

- Collect up to four samples of the country staples - dried carbohydrate/major starch. (Rice, maize, wheat, etc.)
- All food items should be sent to the lab, as concentrations are likely to be lower than the level of detection of the XRF. XRF readings on dry food products can be taken in addition for comparison.
- Reference level: 0.1 ppm for baby formula (WHO); 0.1 ppm for candies (US FDA); 0.5 ppm for food intended for consumption by children (US FDA); 0.1-0.4 ppm for other foods (FAO/WHO)

Other

- Other non-food items – as determined in the country-specific plans. Consider in particular items that are vivid yellow colored (lead chromate) or deep red or orange (other lead salts).

Appendix A: Number of items to be sampled from each of 3 cities

For example, 15 spice samples in each city or 45 total

**What**	**How many**	**Details**	**Select for US Laboratory**
Spices	15/city	If spices were tested during formative research and lead was detected, select those spices with highest lead (3-5 types) and purchase from multiple vendors. If lead was *not* detected in spices during formative research or if spices were not tested, select 3-5 types commonly used in your country and purchase from multiple vendors.	Select 3-5 samples from each city with detectable lead by XRF in medium and high ranges. If no lead is detected, select one sample of each type tested from each city.
Foodware<br>-Metal (Al/other) cooking pots and pans	15/city	Select a variety of most widely sold items used to prepare, store, and contain food, especially hot or acidic food. Do not test utensils or cutlery. If formative research detected lead in cookware or ceramics, select similar types (brands,	Select 2 of each item from each city with detectable lead in medium and high ranges. Pots and pans are the priority. If no lead is detected, select 3 items

-Metal (Al/other) cups and bowls<br>-Ceramic cooking pots, cups and bowls<br>-Plastic cups and bowls		manufacturers). Test a minimum of 5 metal pots or pans, the remainder being cups or bowls made of ceramic, plastic, or metal.	from each city, one metal, one ceramic, and one plastic.<br>*e.g.: 6 aluminum cooking pots (priority); 6 ceramic cooking pots (priority), cups or bowls; 3 plastic cups or bowls*
Toys	10/city	- 5 plastic toys for babies up to age 18 months<br>- 5 painted toys	Select 2 toys from each city (include plastic and painted) with detectable lead in medium and high ranges. If no lead is detected, select 2 plastic baby toys and 1 painted toy.
Paints	10/city	Purchase small quantity or sample size of most commonly purchased types and colors for home or other consumer use (not industrial), according to vendor. Purchase from 2-3 vendors.	XRF testing only; no samples need to be selected for laboratory testing.
Cosmetics	10/city	Select products that are commonly purchased in your country. If henna or kohl or other products with religious significance are used, select 4 or more types of those products. For other cosmetics, select lipstick, nail polish (test like paint), products used on children, and eye color.	XRF testing only is anticipated if XRF detection is at least 10 ppm, which is the reference level. If samples are collected for laboratory analysis, select 2-3 samples of lipstick and/or products used on children from each city.
Major starch	3/city	Select most common nonperishable carbohydrate food(s) typically purchased in your country (rice, flour, wheat, cornmeal, fufu, dried potatoes or potato starch, etc.) from 3 different vendors.	All (~9) samples will be sent to the laboratory because reference levels are lower than XRF detection

Appendix B: Number code for each item type

1	Spices
2	Ceramic foodware
3	Metallic cookware
4	Plastic foodware
5	Herbal medicines
6	Cosmetics
7	Sweets
8	Toys
9	Paints for large surfaces
10	Main starch (e.g. rice, flour etc)
11	Other food
12	Other non-food item
13	Paint craft/art
14	Paint colorant/pigment
15	Paint - unclassified

Appendix C: Number code for each country

NOTE: For India, each state has a unique code.

01 Ghana

02 India - Bihar

03 India - Maharashtra

04 India - Uttar Pradesh

05 India - Gujarat

06 India - Tamil Nadu

07 Indonesia

08 Bangladesh

09 Philippines

10 Colombia

[11] Tajikistan

[12] Kyrgyzstan

[13] Kazakhstan

[14] Georgia

[15] Armenia

[16] Mexico

[17] Peru

[18] Tanzania

[19] Bolivia

[20] Egypt

[21] Morocco

[22] Kenya

[23] Tunisia

[24] Nigeria

[25] Uganda

[26] Pakistan

[27] Nepal

[28] Azerbaijan

[29] Vietnam

[30] Turkey

Appendix D: Guidelines for XRF Use

Correct operation of the XRF is critical for keeping investigators safe and for collecting accurate data.

All investigators operating the XRF should be familiar with the safety information presented in *Chapter 2: Using your analyzer* of Niton XL3 Analyzer User's Guide.

XRF (X-ray fluorescence) is a non-destructive analytical technique used to determine the elemental composition of materials. XRF analyzers determine the chemistry of a sample by measuring the fluorescent (or secondary) X-ray emitted from a sample when it is excited by a primary X-ray source. Each of the elements present in a sample produces a set of characteristic fluorescent X-rays ("a fingerprint") that is unique for that specific element. If you are interested in reading more about how the XRF works, read ThermoFisher's XRF Technology in the Field.

Health and Safety

Primary radiation is radiation that is produced by the analyzer and emitted out through the measurement window.

- **Always treat radiation with respect.**
- **Do not hold your analyzer near the measurement window during testing. Never point your analyzer at yourself or anyone else when the shutter is open.**
- There should always be a sample in contact with the measurement window when the x-ray tube is on.
- The sample will absorb most of the primary-beam radiation unless it is smaller than the instrument's measurement window or of low density and/or thickness. Caution should be taken when analyzing samples that are small, thin, and/or low in density as they may allow much more of the primary beam to escape.

The primary beam is a directed beam out of the front of the analyzer that can have high dose rates. The secondary beam, or scattered beam, has much lower dose rates.

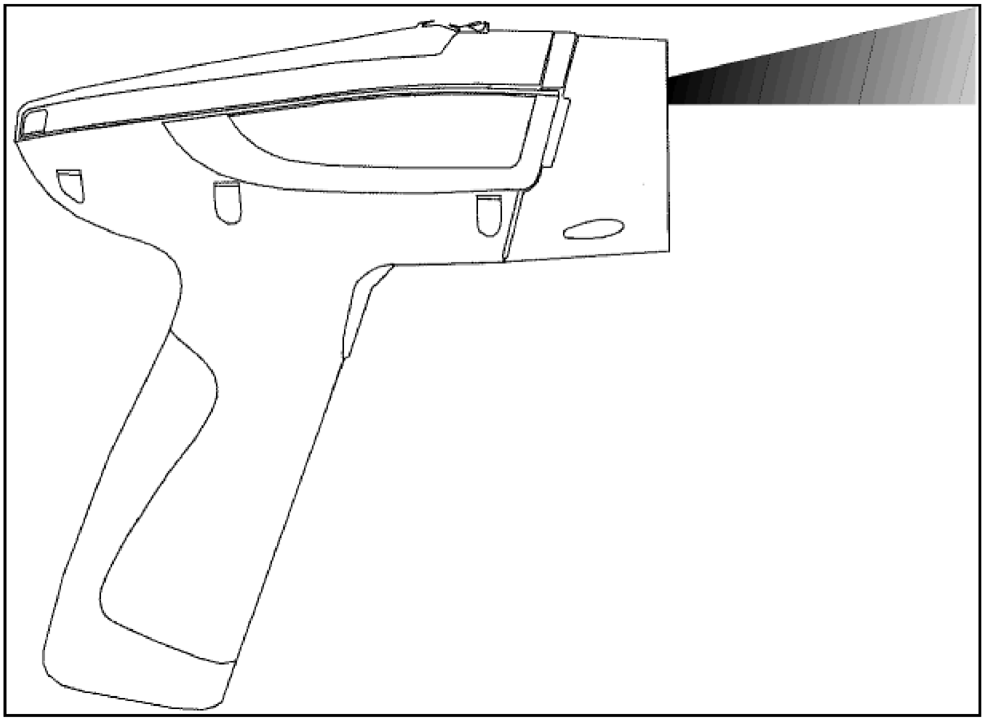

Figure 1. Primary beam

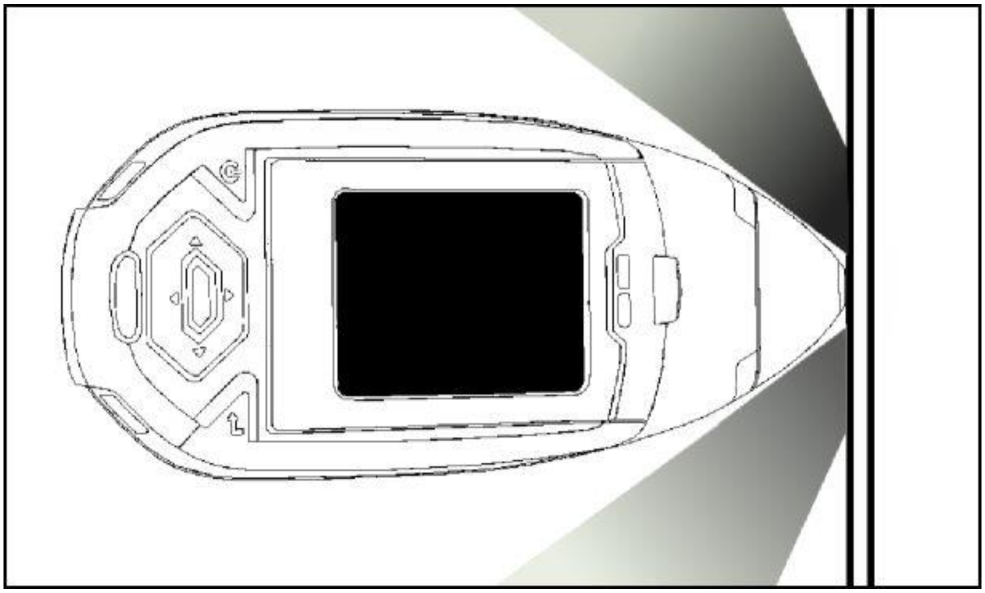

Figure 2. Secondary (scattered) beam

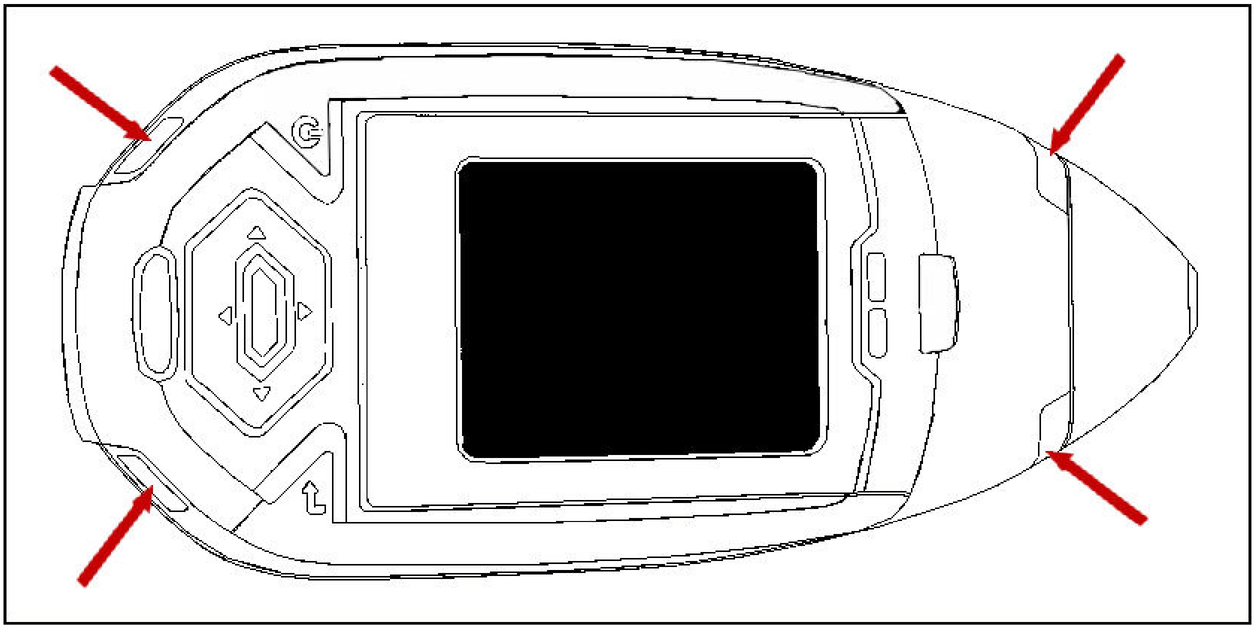

Figure 3. X-ray beam indicator lights

XRF Instrument Preparation

- Inspect the measurement window – circled in red below - for damage (rips) or dirt/debris. Replace if needed (contact HQ).

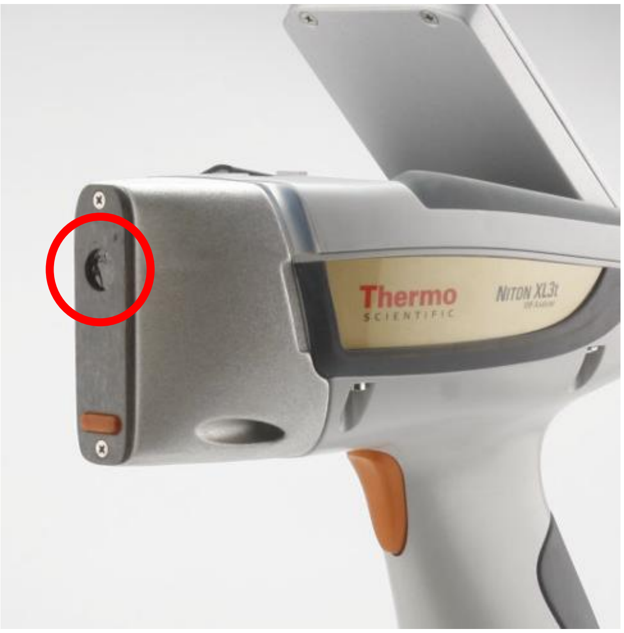

- Each week, select the icon for “System Check.” This allows the instrument to run an internal check – no further interaction is required by the operator. If you receive a failure error, perform a second “System Check.” If you still receive an error, contact NY HQ.
- At the beginning of the day or anytime the instrument has been off for 30 minutes, conduct a calibration verification. Select the appropriate standard for the sample type or matrix you will be analyzing.
- The table below lists appropriate standards for each sample type (or matrix):

**Matrix**	**Expected Pb conc (ppm)**	**Lower acceptable range (-25%)**	**Upper acceptable range (+25%)**
Blank			
SiO2 180-647 (BLANK)	0	ND	10
SiO2 180-428 (BLANK)	0	ND	10
Soil			
SdAR-L2 Soil 180-649A	183	137	229
RCRA 180-661	500	375	625
SdAR-M2 180-706	808	606	1010
NIST 2711a	1400	1050	1750
NIST 2702	133	100	166
Plastic			
PLPE-8ED-31	152	114	190
PN 180-619 Multi-element Reference Sample	150	113	188
Metal			
MBH-1611X SAC305Q	1000	750	1250
MBH Check Sample R 180-696	1200	900	1500

- Record lead reading result for each calibration standard used in the format included in Appendix E.
    - If reading is outside of acceptable range, restart XRF and try again. If still outside range for one or more sample types, contact HQ prior to collecting additional measurements for that mode.
- The calibration verification should also be done at the end of each analysis session.

Sample Preparation

Please see the "Specific Sampling Guidance for Different Products" section of this protocol for sample specific guidance for different materials.

- For loose items like powders, use thin plastic bags (thicker bags may reduce the XRF's signal).
  - Collect "blank" measurement of empty bag to ensure lead is below detectable levels. Record this measurement.
- Mix the sample well to make it more uniform/homogeneous before analyzing.
- For loose items like powders, try to ensure the sample in the bag is ~1cm thick. Denser materials like metal may only need a few millimeters.
- For curved or irregularly shaped items, try to analyze the flattest surfaces.
- Retain all samples (properly labelled and secured) for possible additional screening or laboratory testing.

XRF Operation

- Samples should be placed on a hard surface, not held in your hand when analyzing.
- Take an XRF reading of the table or surface you will be using to analyze the samples to ensure it does not contain lead, as this could interfere with the readings. If lead is detected, find a new surface.
- Ensure XRF is in the appropriate mode for the sample type. We will primarily be using *Test All*.
- XRF readings should be about 30 seconds long.
- If lead is below detection limit, record the detection limit for that reading as displayed on the instrument.

Data Back-up

- At the end of each day when XRF readings are taken, download and retain raw data file as .csv or .xls.
- **NOTE**: Make sure that each XRF reading in the data file is well correlated to each sample reading (e.g., product sample, calibration readings, working surface, blanks, duplicates etc.), otherwise the data file is useless.
- File name should be written with the Country Code and the date that the samples were analyzed as DD-MM-YYYY.
- Contact HQ for assistance with installing and operating the Niton software which allows for downloading the raw XRF data to a PC (Windows only).

Appendix E: Sample XRF Data Collection Table

**Investigator Name:**							Page: ____ of _____
**Location:**							
**Date:**							
**XRF Model:**	**Thermo Niton XL3t 700s**						
**XRF Serial No.:**							
**XRF Standards Check**							
**Sample ID**	**Sample Description**	**XRF Mode**	**XRF Reading Number**	**XRF Reading - Lead (ppm)**	**Standard Error + / - or 2σ**	**XRF Standard Lead Value (ppm)**	**Comments**
MPH-16611X SAC305 R	*Metal Standard "Tin Check"*	*Metals*				***1,000***	
180-661	*Soil Standard "RCRApp 05092201"*	*Soil*				***500***	
180-649A	*Soil Standard "SdAR-L2 Soil"*	*Soil*				***183***	
180-647	*Soil Standard Blank "SiO2 99.995% PP"*	*Soil*				***ND<10***	
180-706 pp	*Soil Standard "USGS SdAR-M2"*	*Soil*				***808***	
PLPE3-8ED-31	*Plastic Standard*	*Plastic*				***152***	
PbPEFIC	Paint Standard (for mass/cm2 only)	*Paint*				**2.31 ug/cm2**	Note: mass concentration is 469 ppm BUT too thin to measure in plastics mode
**Sample Analyses Examples**							
**Sample ID**	**Sample Description**	**XRF Mode**	**XRF Reading Number**	**XRF Reading (ppm)**	**Standard Error + / - or 2σ**		**Comments**
06TAB014	*Plastic Multicolored Toy Car*	*Test All (plastic)*	*131*	*1,300*	*24*		*blue top of car only*
06TAB014	*Plastic Multicolored Toy Car*	*Test All (plastic)*	*132*	*300*	*14*		*black bottom of car only*
14KHU017	*Ground Corn Flour*	*Soil Mode*	*133*	*ND<3*	*Not Applicable*		*XRFd in plastic bag*
11SUB021	*Red Lipstick*	*Test All (plastic)*	*134*	*14.2*	*3*		*XRFd in plastic bag*
11SUB029	*aluminum cookpot*	*Test All (metal)*	*135*	*320*	*32*		*pot interior atop rivet*
**Sample Analyses**							
**Sample ID**	**Sample Description**	**XRF Mode**	**XRF Reading Number**	**XRF Reading (ppm)**	**Standard Error + / - or 2σ**		**Comments**
**For each day of XRF usage, download complete data from XRF in excel format and provide along this table (with hand written notes) to Pure Earth HQ.**							

To download an Excel version of this form, go to:

https://tinyurl.com/RMSXRF
