# Supplementary material for: Rapid Market Screening to assess lead concentrations in consumer products across 25 low- and middle-income countries
Source: Sci Rep. 2024 Apr 27;14:9713. doi: 10.1038/s41598-024-59519-0 (PMC11055946; doi:10.1038/s41598-024-59519-0)
Supplement: Supplementary file 2 — Supplementary Information 2. [file 41598_2024_59519_MOESM2_ESM.docx]

**Supplementary Materials**

**Rapid Market Screening to Assess Lead Concentrations in Consumer Products across 25 Low- and Middle-Income Countries**

**Aelita Sargsyan et al**

**Supplement A: RMS Protocol**

An RMS Protocol was developed by Pure Earth staff with external expert review prior to implementation (available as Annex A on Pure Earth’s RMS web page (<https://www.pureearth.org/wp-content/uploads/2023/09/RMS-Annex-A-RMS-Protocol.pdf)>).

You can find Supplement A as well as PDF file attached:


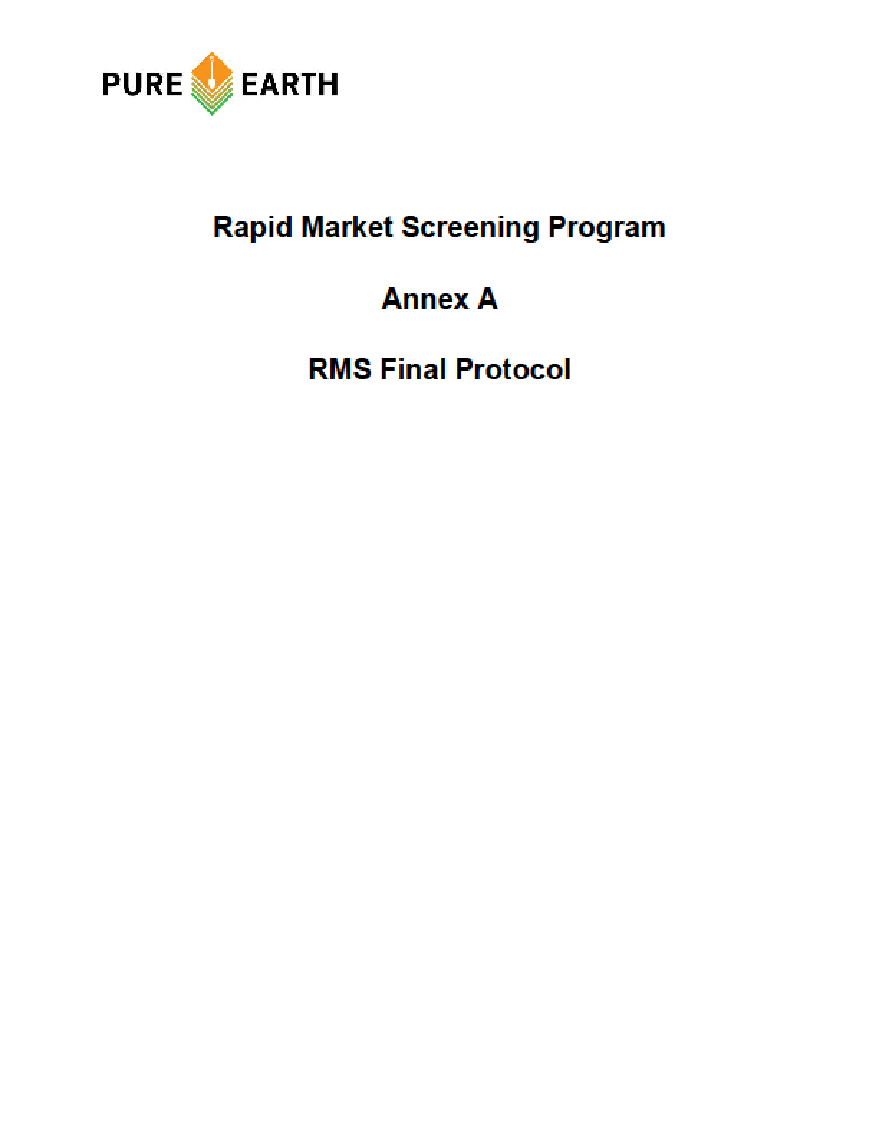


**Supplement B: Quality Control**

Quality Assurance and Quality Control

For this paper, “quality assurance” refers to measures taken to encourage high quality data collection and analysis, such as the development of, and training in a standard RMS implementation protocol (available as Supplement A). “Quality control” refers to processes to identify and correct errors, omissions, or other inaccuracies in the RMS process or data, including confirmatory testing of a subset of samples to validate or invalidate field XRF readings. The following is a summary of RMS quality assurance and quality control process and findings. A more detailed description is available as Annex B on Pure Earth’s RMS web page.

Quality Assurance

Pure Earth’s primary means of encouraging quality data collection and analysis included the development of a standardized RMS Protocol guiding the methods and sequencing of each step of the program, the training of RMS Investigator teams in that protocol, and ongoing supervision and re-training of teams as the program was implemented.

Pure Earth divided the implementation of the RMS program into three phases: a Formative Research Phase in which we piloted the methodology in several countries to troubleshoot any implementation hurdles; Phase 1, in which the first 15 countries implemented the program; and Phase 2, in which the final 10 countries implemented the program. After each phase, the RMS team reviewed challenges and lessons from the implementation and made any necessary revisions to the protocol and training. Notable changes made after the formative phase included: guidance to specify and narrow the types of items purchased and analyzed; additional guidance on the number of samples desired; and clarification of the XRF setting to use during analysis. After Phase 1, the protocol was amended to shift from analyzing wet paint samples to dried paint swatches (to allow transport of the samples). Aside from the paint analysis method, the protocol did not differ substantially between phases and the analytical results from both Phases 1 and 2 are included here.

Quality Control

The primary quality control measures in the RMS program included:

1. Regular communication with, and oversight of RMS Investigator teams to ensure they understood the protocol, felt comfortable in their plans, and to address any challenges or questions.
2. Ongoing reviews of data uploaded into the SurveyCTO database platform to ensure that RMS Investigators collected appropriate sample types and numbers of samples, and that sample logs contained all required information.
3. A review of descriptions and item categorization choices made by RMS Investigators (e.g., are leafy herbs a spice, a medicine, or “other foods?”) to ensure consistency with the RMS protocol and the descriptions and categorization choices made by other country teams.
4. A review of XRF field data uploaded by RMS Investigator teams to the SurveyCTO database to identify any formatting, unit, or input errors and to highlight any outliers that might require further quality control inquiries.
5. Shipping of a subset of more than 1,000 samples (approximately 20%) to New York for confirmatory analysis with an XRF analyzer in Pure Earth’s headquarters that was operated by an experienced expert, was known to be in good working order, had shown consistency with lab results, and was regularly calibrated against a “standard” sample with a known lead concentration.
6. Confirmatory analysis of a subset of 354 samples by accredited laboratories using analytical methods known to be more accurate and sensitive than XRFs.

Summary Of XRF Performance As A Screening Tool

Lead concentrations measured with portable XRF analyzers proved highly comparable to results from laboratory analysis with the following two exceptions. First, the XRFs appeared to inflate lead concentrations among four samples for which lab results exceeded 30,000 ppm (3% lead), suggesting that the XRF’s accuracy may have decreased at very high lead concentrations. Second, confirmatory screening with a New York-based XRF and lab testing suggested that one of the XRFs that was used in both Tajikistan and Kazakhstan provided inaccurate field results; accordingly, the field data from these countries were expunged by the RMS Quality Control team. We do not know if this was due to equipment malfunction, contamination of the XRF measurement window, or another type of user or mechanical error. In the case of these two countries, only samples that were sent to New York and analyzed with an XRF that proved to be consistent with lab results were included in this report.

Finally, we also note that we could not compare XRF results with lab results for items with comparatively low lead concentrations due to the lower detection limit of the XRFs, which for most materials is approximately 2-4 ppm, compared to 0.2-0.5 ppm in the lab. This fact does not suggest inaccuracy among the XRFs but is simply an analytical limitation of the device.

| **Item Type** | **# of samples sent for  lab analysis** |
| --- | --- |
| Ceramics | 1 |
| Cosmetics | 64 |
| Staple Dry Foods | 65 |
| Miscellaneous | 9 |
| Other Foods | 15 |
| Plastic Foodware | 17 |
| Spices | 125 |
| Toy - Painted | 3 |
| Toy - Plastic/Rubber | 57 |
| **Total** | **356** |

Expunging Field XRF Data From Tajikistan And Kazakhstan

Despite the general consistency between field XRF, New York-based XRF, and lab results, the field XRF readings from Tajikistan and Kazakhstan had significant discrepancies across several item types when compared to New York-based XRF and lab results. Ultimately, the Quality Control team expunged all field XRF data from these countries. Only samples that were sent to New York for analysis by a New York-based XRF or lab are included in this report.

Correlations Between XRF And Lab Results

*Toys*: There was excellent correlation between XRF and lab results for the plastic toy samples, with a correlation coefficient of 0.9597. Among the 19 toy samples for which the XRF did not detect lead, the lab analysis also reported no lead (ND<0.50 ppm)) or reported lead up to a level of 6 ppm, significantly below the reference standard.

*Cosmetics*: A total of 64 cosmetics samples were submitted for laboratory testing, including eye shadow, eyeliner, lipstick, face powders, and henna. The 43 cosmetic samples in which the lab detected lead below 1,000 ppm correlated very well with the field XRF results (R^2^ = 0.7754). There was one sample of kohl from Tunisia for which the XRF measured 712 ppm for which the lab reported lead at a concentration of 5.08 ppm. There were four cosmetics samples in which the lab found extremely high lead concentrations ranging from 33,000 to 320,000 ppm. Among these samples, the XRF deviated substantially. The two items with the highest lead concentrations were both eyeliners, known as kajal or kohl, from Pakistan. These samples had concentrations of 637,600 ppm and 1,000,000 ppm (equivalent to 64 and 100%, respectively) based on XRF measurements. However, laboratory testing of these two samples indicated significantly lower concentrations (29% and 32%, respectively), albeit still extremely elevated. Given the accuracy of the XRF at lower lead levels, the results suggested to the Quality Control Team that the XRF’s accuracy may simply diminish at extreme lead concentrations.

*Staple Dry Foods*: A total of 65 samples of dry foods representing local dietary staples were submitted for laboratory testing, including various grains, gain flours, and legumes. Lead was not detected by the lab in 48 of the samples. Lab results for 14 samples showed levels below 1 ppm, which is below the XRF lower detection limit. There was only one sample with lead detected by the XRF at a concentration of 18 ppm with a corresponding laboratory report of 14 ppm for that sample. There was one sample for which the XRF measured 16.46 ppm for which the lab reported ND<0.1 ppm. Lastly, there were two samples for which the XRF did not detect lead (ND<2 ppm) for which the laboratory reported lead at 1.9 and 5.6 ppm.

*Plastic Foodware*: A total of 17 plastic foodware items were submitted for lab testing. There was a fair correlation between XRF and lab results, with a correlation coefficient (R^2^) of 0.7735.

*Spices*: A total of 125 spices were submitted for laboratory testing. Laboratory results for 9 samples are pending. The high number of spice samples sent to the lab reflects the fact that the reference level for spices is near the lower detection limit of XRFs. Types of spices sent to the lab included turmeric, chili, tamarind, peppers, sunelli, and ginger, among others. Lead was not detected by the laboratory in 44 of the samples. Lead was detected by the laboratory at concentrations of <2 ppm in 631 of the 125 samples and at concentrations of between 2 ppm and a high of 320 ppm in 18 of the 125 samples. For samples with laboratory lead concentrations >1 ppm, there was an excellent correlation between XRF and lab results, with a correlation coefficient (R^2^) of 0.9399, with the XRF results coming in slightly higher than lab results. Note that three samples with the highest lab results, ranging from 66 to 320 mg/kg, were turmeric samples obtained from 3 different markets in Tajikistan. There is some uncertainty in the actual levels of lead in spice samples from Pakistan, which is discussed in [Annex B](https://www.pureearth.org/wp-content/uploads/2023/11/RMS-Annex-B-Quality-Assurance-and-Quality-Control.pdf) to Pure Earth’s RMS summary report.

Overall, the XRFs proved to be an excellent screening tool for detecting elevated lead levels in products, particularly among items for which the reference level exceeds the XRF’s lower detection limit. More information regarding the RMS Quality Control methods and comparisons between XRF and lab results which can be found in [Annex B](https://www.pureearth.org/wp-content/uploads/2023/11/RMS-Annex-B-Quality-Assurance-and-Quality-Control.pdf) to Pure Earth’s RMS summary report. .

**Supplement C: Lead in Consumer Goods by Product**

Findings by Product Type

Out of a total of 5,007 product samples from 25 countries, 913 samples had concentrations of lead exceeding the relevant reference level based on XRF readings, representing 18% of all samples.

As shown in the table below, metallic foodware, ceramic foodware, and paints most frequently exceeded the relevant reference levels.

Summary of Sample Distribution by Product Category

| **Product Category** | **Total # of Samples** | **Min Value (ppm)** | **25th% (ppm)** | **Median (ppm)** | **75th% (ppm)** | **Max Value (ppm)** | **% Above Reference** |
| --- | --- | --- | --- | --- | --- | --- | --- |
| Ceramic foodware | 308 | ND | ND | 73 | 3665 | 397100 | 45 |
| Metallic foodware | 520 | ND | ND | 118 | 754 | 119500 | 51 |
| Plastic foodware | 364 | ND | ND | ND | ND | 3289 | 12 |
| Cosmetics | 812 | ND | ND | ND | ND | 1000000 | 12 |
| Toys | 781 | ND | ND | ND | 13 | 97300 | 13 |
| Paint - large surface | 437 | ND | ND | 1 | 1518 | 807309 | 41 |
| Paint - craft/art | 70 | ND | ND | ND | ND | 93500 | 11 |
| Paint - unclassified | 102 | ND | ND | 10 | 3400 | 79000 | 47 |
| Spices | 1084 | ND | ND | ND | ND | 622 | 2 |
| Sweets | 111 | ND | ND | ND | ND | 5 | 3 |
| Staple Dry Foods | 364 | ND | ND | ND | ND | 17 | 1 |
| Herbal/Trad Medicines | 54 | ND | ND | ND | ND | 31 | 4 |

ND = “non-detect” (lead concentration was below the XRF’s lower detection limit)

In Figure 1 the percentage of samples above reference level is shown graphically, to illustrate the magnitude of lead contamination.

*
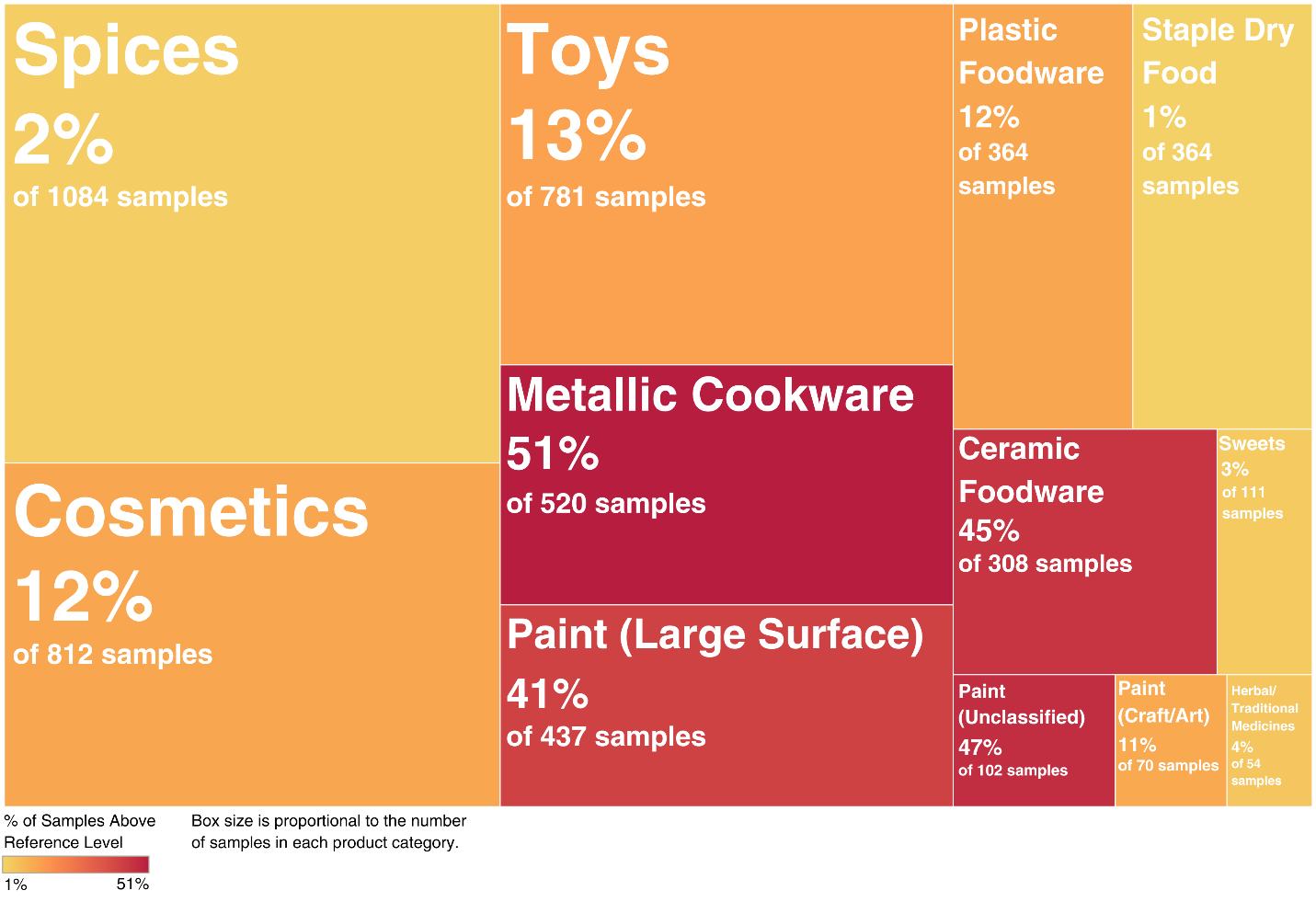
*

Figure 1: Percentage of samples above reference level

Ceramic Foodware

One of the most interesting RMS findings is the high prevalence and wide geographic distribution of ceramic foodware with elevated lead levels. Out of 308 ceramic foodware samples analyzed across the 27 study locations, 45% had lead concentrations exceeding the reference level of 100 ppm.

Many of the ceramic samples analyzed in the RMS had heterogeneous coatings, with multiple colors or differences between interior and exterior coating. Investigators were asked to take at least 3 readings per item, prioritizing measurements in the inside of the item where food or drink would come in contact. As with all categories, the highest lead reading was used where we had multiple readings for the same item.

The use of lead-based glazes has been well documented in Mexico and several other Latin American countries, but the RMS reveals that contamination is highly prevalent across all regions. That is not to say that all regions have the same exposure risks. The leachability of lead from ceramics coatings is influenced by the type of glaze and the conditions under which it is fired and used. Certain lead-based glazes fired under comparably low temperatures in wood-burning kilns in Mexico, for example, have been shown to be highly leachable in the presence of hot or acidic foods and are believed to contribute substantially to lead exposures. Other glazes that contain lead but are fired at higher temperatures may leach less and thus contribute less to exposure. Additionally, ceramic products that contain lead, but which do not contact hot or acidic foods may leach less. The leachability of lead from various ceramic glazes produced and used under different conditions is an area that requires further research.

Summary of Ceramic Foodware Results by Country

| **Country name** | **# of Samples** | **Min Value (ppm)** | **Median Value (ppm)** | **Maximum Value (ppm)** | **% Above Reference** |
| --- | --- | --- | --- | --- | --- |
| Armenia | 11 | ND | 58 | 9280 | 36 |
| Azerbaijan | 13 | 312 | 774 | 11400 | 100 |
| Bangladesh | 9 | ND | 22 | 4636 | 44 |
| Bolivia | 10 | 35 | 131853 | 397100 | 60 |
| Colombia | 18 | ND | 237 | 29100 | 50 |
| Egypt | 10 | ND | 158 | 50600 | 50 |
| Georgia | 27 | ND | 76 | 13200 | 48 |
| Ghana | 11 | 30 | 50 | 6570 | 18 |
| India - Maharashtra | 17 | ND | 1910 | 80000 | 71 |
| India - Tamil Nadu | 8 | ND | 75 | 5230 | 50 |
| India - Uttar Pradesh | 6 | ND | 20 | 80 | 0 |
| Kenya | 21 | ND | 4210 | 91000 | 62 |
| Kyrgyzstan | 16 | ND | 73 | 240500 | 44 |
| Mexico | 6 | ND | 19215 | 65700 | 67 |
| Nepal | 11 | ND | ND | 9220 | 18 |
| Nigeria | 21 | 20 | 40 | 46000 | 29 |
| Pakistan | 5 | ND | 69 | 103 | 20 |
| Peru | 10 | ND | 124 | 18600 | 50 |
| Philippines | 15 | ND | 35 | 1159 | 13 |
| Tajikistan | 3 | 100700 | 133400 | 266000 | 100 |
| Tanzania | 3 | 20 | 7780 | 22300 | 67 |
| Tunisia | 16 | ND | 251 | 68600 | 56 |
| Turkey | 15 | ND | 119 | 14300 | 53 |
| Uganda | 12 | 17 | 23 | 6092 | 8 |
| Vietnam | 14 | ND | 59 | 19789 | 29 |

ND = “non-detect” (lead concentration was below the XRF’s lower detection limit)

Lead Concentration Results by Country
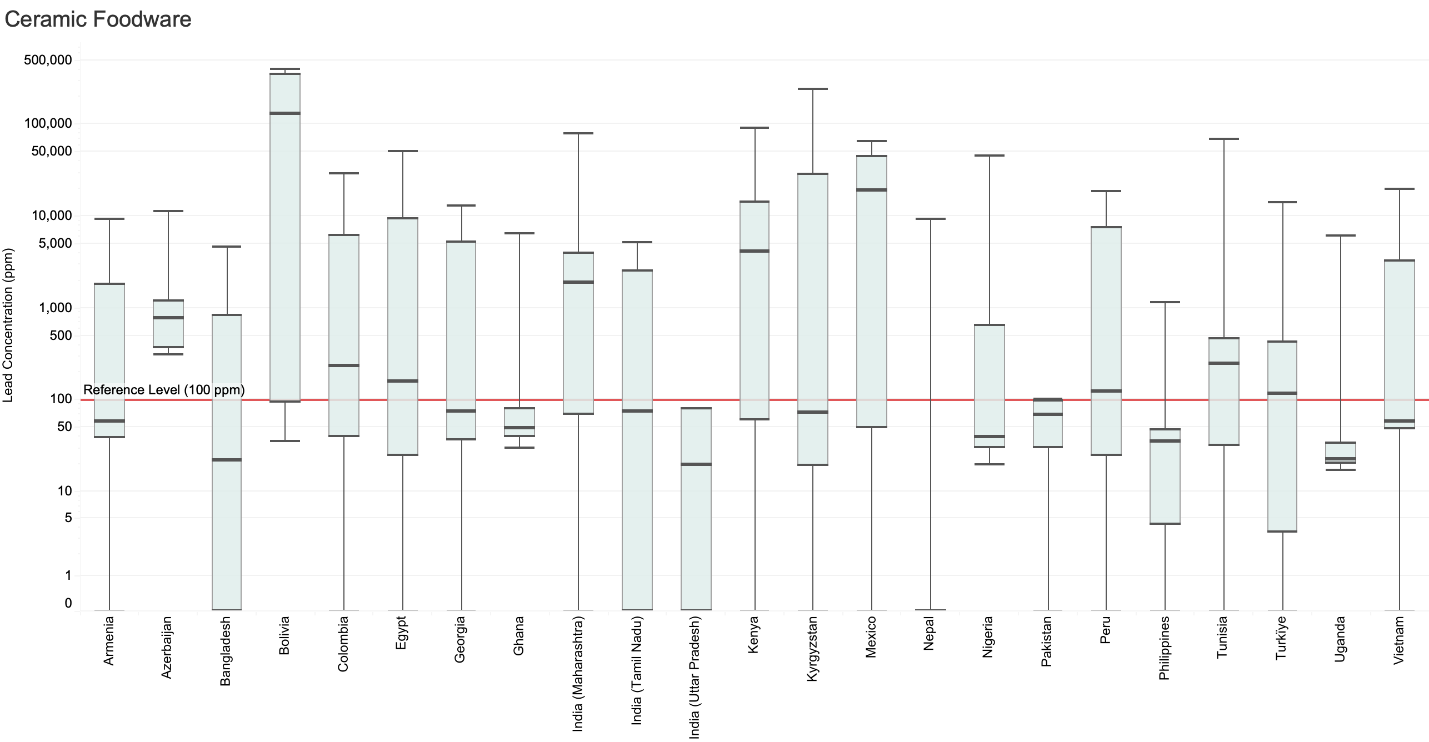


**Note**: Sample sizes below 5 are not displayed.

Metallic Foodware

Out of 520 samples of metallic foodware, 51% exceeded the reference level of 100 ppm. Previous studies have highlighted elevated lead levels in metallic foodware made in LMICs, particularly in inexpensive [aluminum foodware](https://www.sciencedirect.com/science/article/pii/S0048969716324548#ks0010). These pots are generally light, inexpensive, and have good conductivity, which helps conserve fuel usage. Some of this foodware is believed to be made from mixed recycled metals from engine parts, radiators, aluminum cans, and construction materials.^^[[1]](#footnote-1)^^ Among Pure Earth’s recommendations is the need to further identify lead sources contributing to contamination in recycled metals.

This study analyzed a wide variety of foodware items and materials. Samples included both small-batch, informally produced items, as well as large-scale, commercially produced items. Of the items in the metallic foodware category that were found to be above the reference level, 69% were pots and pans, 17% were vessels for food or water not intended to be exposed to direct heat, and 14% were cooking utensils.

Across all item types, 57% of the items found to be above the reference level were reported to be made of, or labeled as aluminum or aluminum alloys. For 35% of the items, we were not able to determine the metal composition based on the item description or label. None of the other metal types—including brass, copper, and iron alloys—exceeded 5% of the total number of items found to be above the reference level.

Readers should note that the total lead content in foodware samples does not reflect leachable lead. To improve our understanding of potential doses of lead per use, Pure Earth is conducting leachability testing of more than 100 aluminum foodware samples. A description of this program and the early findings is included in this section below the following tables and charts.

Summary of Metallic Foodware Results by Country

| **Country name** | **# of Samples** | **Min Value (ppm)** | **Median Value (ppm)** | **Max Value (ppm)** | **% Above Reference** |
| --- | --- | --- | --- | --- | --- |
| Armenia | 19 | ND | ND | 287 | 11 |
| Azerbaijan | 16 | ND | 178 | 2342 | 63 |
| Bangladesh | 27 | ND | 186 | 8186 | 59 |
| Bolivia | 13 | ND | 164 | 2049 | 54 |
| Colombia | 35 | ND | 51 | 2679 | 40 |
| Egypt | 11 | ND | 180 | 1086 | 55 |
| Georgia | 19 | ND | ND | 119500 | 16 |
| Ghana | 22 | ND | 181 | 24100 | 55 |
| India - Maharashtra | 19 | ND | 720 | 6590 | 63 |
| India - Tamil Nadu | 27 | ND | 870 | 13900 | 70 |
| India - Uttar Pradesh | 17 | ND | 850 | 74600 | 65 |
| Indonesia | 45 | ND | 410 | 18100 | 60 |
| Kenya | 15 | 21 | 130 | 3600 | 53 |
| Kyrgyzstan | 16 | ND | 8 | 494 | 19 |
| Mexico | 16 | ND | 30 | 900 | 25 |
| Nepal | 11 | 170 | 750 | 3960 | 100 |
| Nigeria | 35 | ND | 410 | 1000 | 66 |
| Pakistan | 28 | ND | 3238 | 7858 | 75 |
| Peru | 28 | ND | 176 | 90400 | 64 |
| Philippines | 17 | ND | 26 | 1253 | 24 |
| Tanzania | 17 | ND | 30 | 15100 | 35 |
| Tunisia | 25 | ND | ND | 26600 | 12 |
| Turkey | 9 | ND | 171 | 903 | 67 |
| Uganda | 15 | ND | 303 | 1564 | 73 |
| Vietnam | 18 | ND | 269 | 13080 | 56 |

ND = “non-detect” (lead concentration was below the XRF’s lower detection limit)

Lead Concentration Results by Country
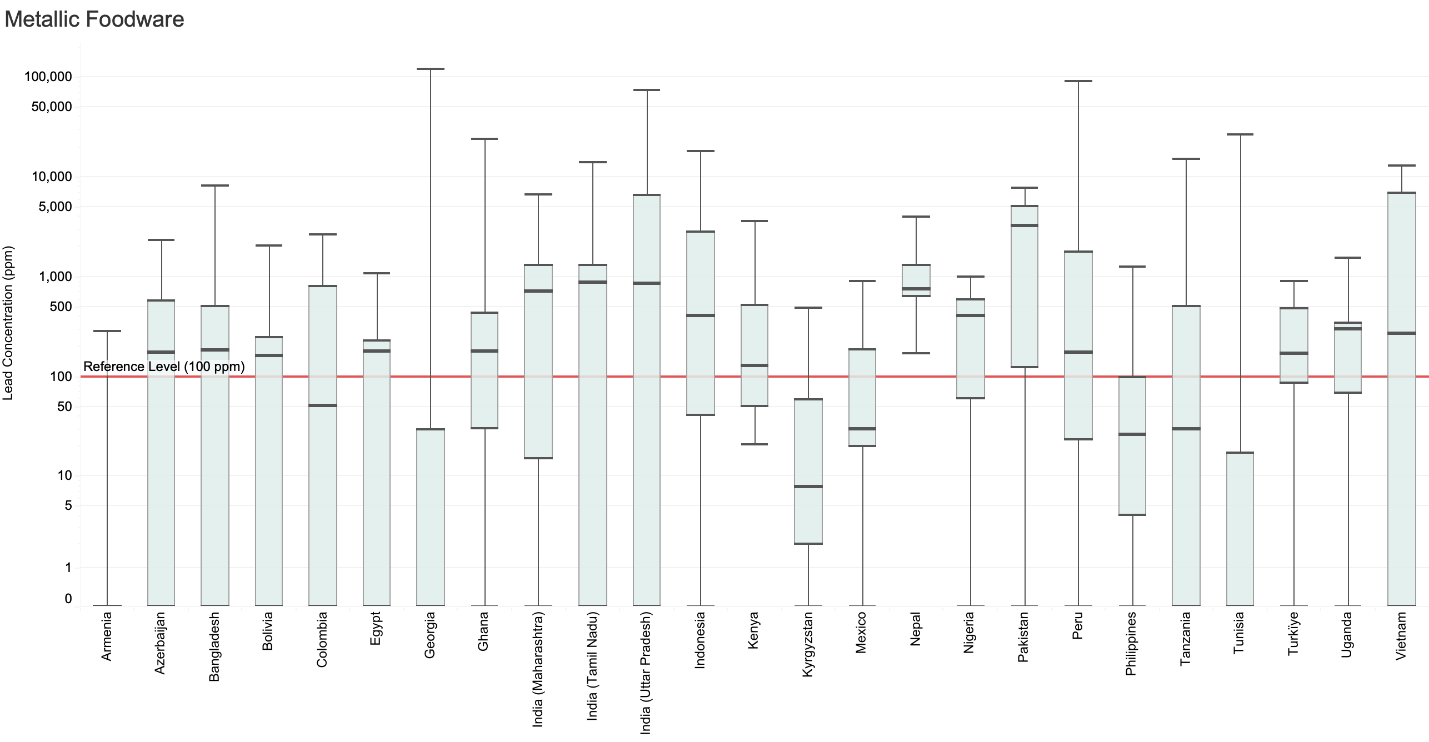


**Leachate Testing of Aluminum Cookware**

As noted above, Pure Earth has analyzed a subset of 92 aluminum cookware samples, collected through the RMS, in a commercial research laboratory to model conditions that may occur when using the pots for cooking. This research is ongoing and the full results will ultimately be shared in a subsequent publication. However, the preliminary findings are notable and help shed light on the possible contribution of contaminated aluminum cookware to elevated blood lead levels.

The objectives of this research are:

- Evaluate the potential for aluminum pots to be a source of lead exposure;
- Evaluate the relationship between lead levels measured by XRF to lead concentrations that may be released during cooking; and
- Refine a testing protocol that is practical and reproducible.

Pots were selected for leachate testing from 23 of the RMS countries. The pots varied in shape, size, and finish from country to country. Some examples are shown below:


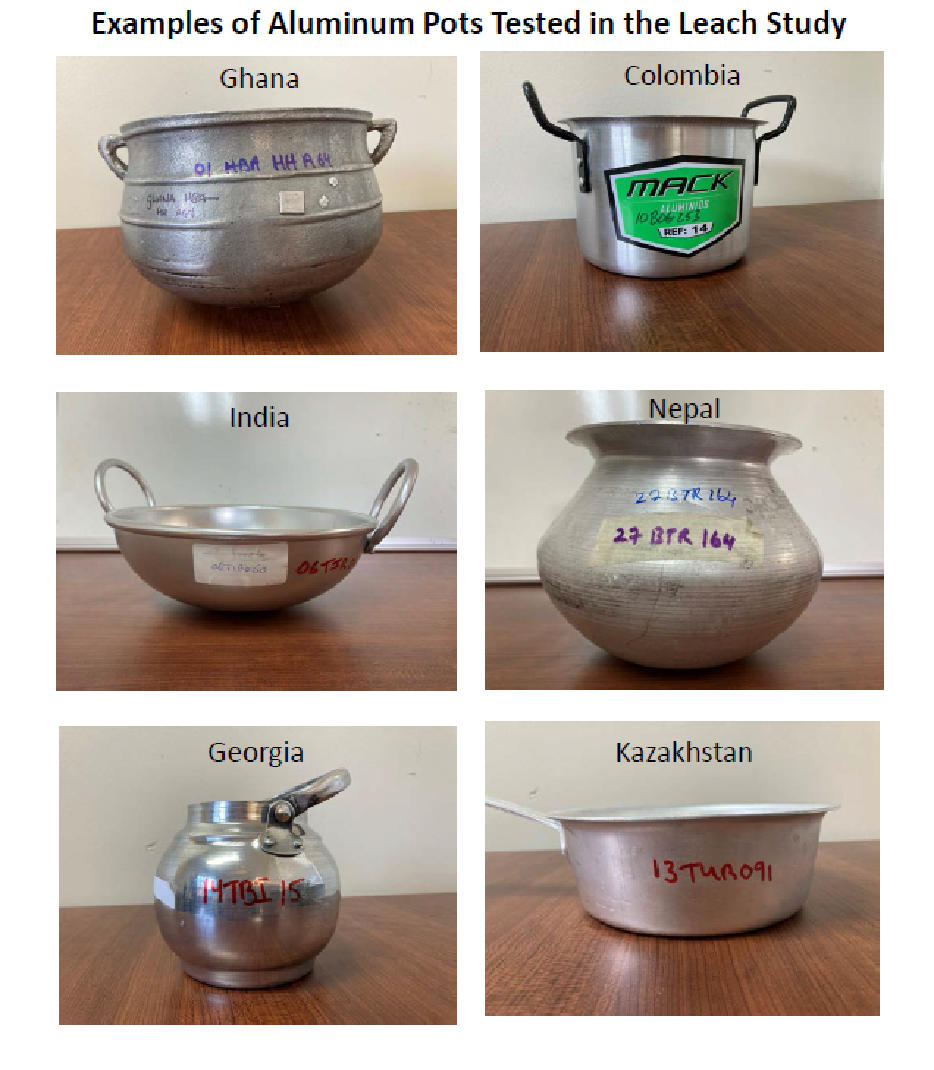


There is no standard method for testing aluminum pots for lead release during cooking. The method used by Pure Earth is similar to those used by other researchers. The method models cooking acidic food for an extended period to leach or solubilize lead from the interior surface of a pot. In these studies, acidic water (i.e., 4% solution of acetic acid), termed leachate, is used to model acidic food. The method involves:

- Filling the pot with a 4% solution of acetic acid
- Bringing the pot to a boil and boiling, covered, for 2 hours
- Collecting the sample while hot, then processing the sample to dissolve lead compounds that may have formed particles in the leachate
- Analyzing the leachate for lead and aluminum using standard US EPA methods^^[[2]](#footnote-2)^^

**Reference Level for Leachate**

Pure Earth used a preliminary reference level of 10 ug/L (10 parts per billion) lead in the leachate to evaluate the potential for a pot to be a source of lead exposure. The reference level considers the following existing criteria for water and food:

- The US EPA action level for drinking water is 15 μg/L;
- The World Health Organization provisional guideline is 10 μg/L for drinking water; and,
- The US FDA Interim Reference Limit (IRL) is 2.2 μg/day in food for children. A daily serving of 250 ml or grams is a standard assumption; therefore 2.2 μg/day x day/250 ml x 250 ml/L = 8.8 μg/L, which is close to the 10 μg/L reference level.
  - In application, a child aged 0 to 7 years old ingesting 250 ml or grams of food every day, with a lead concentration equal to the 10 μg/L reference level, would result in an average blood lead level of 0.47 μg/dL. These calculations are derived from the US EPA’s *Integrated Exposure Uptake Model for Lead in Children (IEUBK)^^[[3]](#footnote-3)^^.*

**Lead Concentrations in Leachate**

Of the 92 pots tested, 48 (52%) had lead concentrations in leachate exceeding the screening level of 10 ug/L. The average lead concentration in leachate was 98 ug/L, while the median concentration was 12 ug/L.

For those samples exceeding the 10 μg/L reference level, most were in the range of 10 to 99 ug/L and averaged 39 ug/L (35% of total pots), followed by pots with leachate concentrations ranging from 100 to 999 ug/L and averaging 248 ug/L (15% of total pots). A small number of pots leached lead at concentrations exceeding 1,000 ug/L, averaging 2,080 ug/L (2% of total pots). For pots where the 10 μg/L reference level was not exceeded, most were in the range of 1-9.9 ug/L, averaging 5.1 ug/L (37% of total pots). Lead was nondetectable in leachate for 11% of the pots.


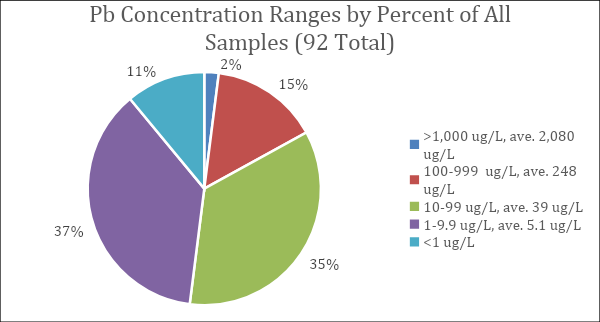


**Regional and Country Variations**

Lead concentrations in leachate and exceedances of the 10 μg/L reference level varied by geographic region and country. The numbers of samples collected and analyzed by country and region were not equal, which potentially skews the results, but the trends are worth noting. The majority of the pots analyzed in this study were collected in South Asia or Southeast Asia, including India, Indonesia, Bangladesh, the Philippines, Nepal, Pakistan, and Vietnam, and 84% of the pots from this region exceeded the screening level of 10 ug/L. A moderate number of samples were collected from Africa (Ghana, Nigeria, Tunisia, Kenya, Tanzania), and 43% of the samples exceeded the 10 μg/L reference level. The results are consistent with observations in these regions, where many of the aluminum pots found in markets are made locally from a variety of scrap sources that could contain lead. Pots from Eurasia and the Americas had fewer exceedances or none at all, and had the appearance of being factory-made.


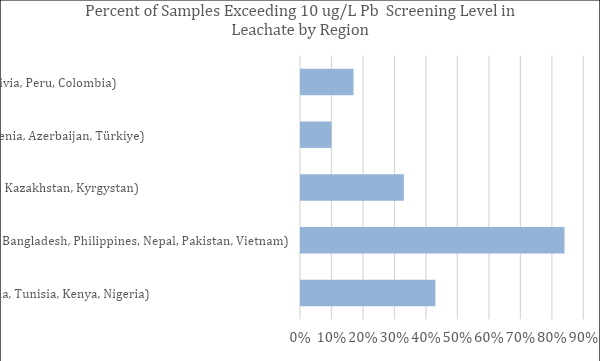


The graphs below show average and maximum lead concentrations in leachate by country, where maximum concentrations are (1) between 10 and 100 ug/L and (2) greater than 100 ug/L, respectively. Maximum and average leachate concentrations from pots from eight countries (Azerbaijan, Tanzania, Mexico, Kazakhstan, Colombia, Peru, Armenia, and Georgia) did not exceed the reference level of 10 ug/L. Average and maximum lead concentrations in leachate were in the range of 10 to 100 ug/L in pots from Kyrgyzstan, Nigeria, Turkey, Tajikistan, Bolivia, India, and the Philippines; the remaining countries of Tunisia, Pakistan, Kenya, Ghana, Nepal, Vietnam, Bangladesh, and Indonesia exceeded 100 ug/L on average. The most pots per country (eight) were analyzed from Indonesia and Pakistan. Indonesia had the highest maximum lead concentration (2,900 ug/L) and average lead concentration (617 ug/L). Six of the eight pots tested from Indonesia exceeded the 10 μg/L reference level. All pots from Pakistan exceeded the 10 μg/L reference level, although at lower concentrations than Indonesia, with a maximum of 132 ug/L and an average of 74.2 ug/L.


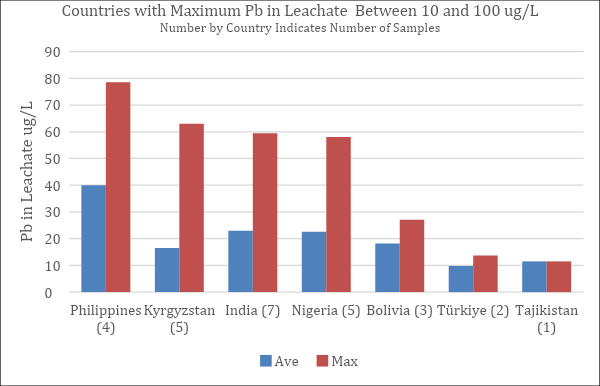


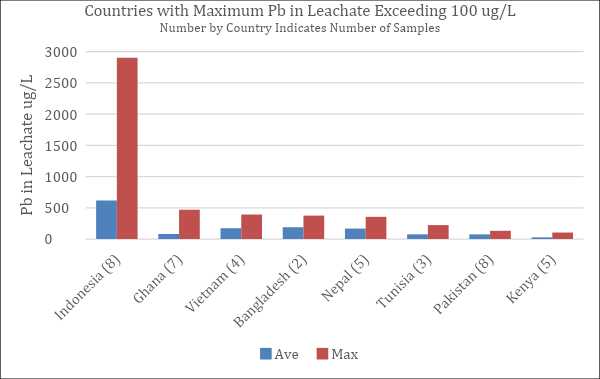


**Comparison of XRF Readings to Lead Concentrations in Leachate**

The XRF analyzer has been a useful screening tool for aluminum pots. XRF readings for lead in aluminum foodware ranged from nondetectable to greater than 10,000 ppm, with an average of 1,407 ppm and a median value of 402 ppm. When comparing lead in leachate to XRF readings for all pots, the correlation is low, with an R^2^ coefficient of 0.28. When data at the lower end of the scale is plotted, looking at data pairs where the XRF readings are less than 500 ppm, the correlation is even lower, with an R^2^ coefficient of 0.25.


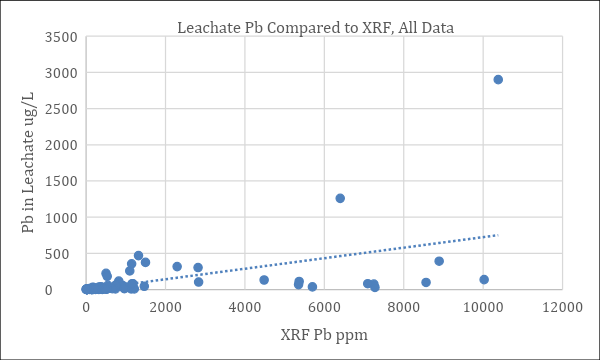


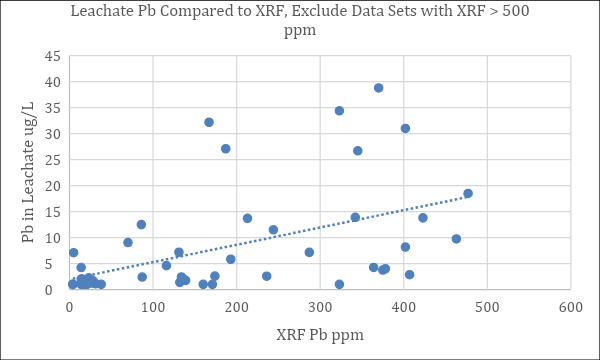


There is not a strong linear correlation between leachable lead and total lead in the pots tested. Notably, total XRF lead below 100 ppm is a very good indicator that the pot would leach less than the 10 ug/L reference level. Of the 18 pots tested with less than 100 ppm total lead, only one pot, with total lead of 86 ppm, indicated a leachate concentration of 12.5 ug/L, exceeding the 10 ug/L reference level. Conversely, XRF readings of total lead greater than 100 ppm provides a working screening level of the potential for leaching above the reference level of 10 ug/L about 70% of the time. That is, in about 30% of the samples with XRF readings above 100 ppm, lead concentrations in leachate did not exceed 10 ug/L. We consider 100 ppm total lead in aluminum pots and pans to be a working screening level for pots leaching above or below the 10 ug/L reference level.

It is important to note that the actual amount leaching from any pot must be determined using representative leaching tests. The use of a 100 ppm total lead screening level to evaluate the potential for leaching is an estimate, particularly considering leaching data in the literature that has shown the potential to leach at total lead values below the 100 ppm threshold. It is also important to note that there are many factors affecting the amount of lead leaching from any particular pot, including temperature, contact time, leaching medium, pot coatings, condition and age of the pots, among other factors.

**Potential for Lead Leached from Cooking Pots to Result in Elevated Blood Lead Levels**

To evaluate the potential for aluminum pots to be a source of lead exposure, computations were made using US EPA’s *Integrated Exposure Uptake Model for Lead in Children (IEUBK).* The assumptions in the model were that food consumed by the child would contain lead leached from the pot, and that a child would consume a daily 250-mL portion of food cooked in the pot.

The concentrations of lead in food in the model reflected the range of leachate concentrations observed during these tests, from 1 ug/L to 3,000 ug/L. The model assumed an uptake of 50% of the lead ingested. IEUBK generates data for different age ranges for children from 0 to 7 years (6-12 months, 12-24 months, etc.). For the purposes of this comparison, results across the age ranges from 0 to 7 years were averaged. The table of projected blood lead levels (BLLs) resulting from daily ingestion of food (leachate) containing lead in a range of concentrations is shown in the table and graph below. Importantly, a child aged 0 to 7 years old ingesting 250 ml or grams of food every day with a lead concentration equal to the 10 μg/L reference level would see an average increase in blood lead level of 0.47 μg/dL.

| Leachate Concentration ug/L | Average BLL ug/dL for Child 0-7 years |
| --- | --- |
| 1 | 0.03 |
| 5 | 0.23 |
| 10 | 0.47 |
| 50 | 2.24 |
| 80 | 3.45 |
| 100 | 4.27 |
| 500 | 16.04 |
| 1,000 | 25.47 |
| 3,000 | 48.39 |


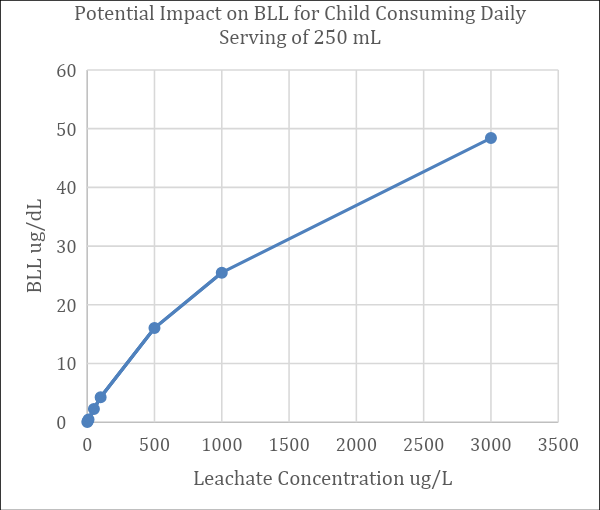


The US Centers for Disease Control use a blood lead reference level 3.5 ug/dL as a threshold for recommending intervention in a child’s environment to reduce lead exposure. Using data from the graph above, a food (or leachate in the lab setting) with a lead concentration of about 80 ug/L could result in a blood lead level above 3.5 ug/dL if food is consumed on a daily basis. In our studies, 21% of the pots tested produced a leachate of 80 ug/L or greater, indicating that there are circumstances under which aluminum pots could be a considerable contributor to elevated blood lead levels.

Plastic Foodware

Out of 364 plastic foodware samples, 12% showed lead levels exceeding the reference level of 100 ppm. RMS Investigators were asked to prioritize items used by children for food consumption and storage, particularly bowls and cups. Unlike ceramic and metallic foodware, for which many countries had samples with maximum concentrations above 10,000 ppm, all samples of plastic foodware were below 3,300 ppm.

Summary of Plastic Foodware Results by Country

| **Country name** | **# of Samples** | **Min Value (ppm)** | **Median Value (ppm)** | **Max Value (ppm)** | **% Above Reference** |
| --- | --- | --- | --- | --- | --- |
| Armenia | 18 | ND | ND | 478 | 6 |
| Azerbaijan | 5 | ND | 211 | 1196 | 60 |
| Bangladesh | 11 | ND | ND | 672 | 9 |
| Bolivia | 14 | ND | ND | 2073 | 14 |
| Colombia | 17 | ND | ND | 1687 | 24 |
| Egypt | 15 | ND | ND | 1121 | 13 |
| Georgia | 5 | ND | ND | ND | 0 |
| Ghana | 12 | ND | ND | 32 | 0 |
| India - Maharashtra | 16 | ND | ND | 437 | 19 |
| India - Tamil Nadu | 7 | ND | ND | 872 | 14 |
| India - Uttar Pradesh | 18 | ND | ND | 11 | 0 |
| Kenya | 12 | ND | ND | 2395 | 25 |
| Kyrgyzstan | 16 | ND | ND | 368 | 13 |
| Mexico | 13 | ND | ND | 853 | 8 |
| Nepal | 17 | ND | ND | 348 | 6 |
| Nigeria | 24 | ND | ND | 280 | 4 |
| Pakistan | 12 | ND | ND | 2419 | 8 |
| Peru | 18 | ND | ND | 1643 | 17 |
| Philippines | 14 | ND | ND | ND | 0 |
| Tanzania | 18 | ND | ND | 2791 | 17 |
| Tunisia | 23 | ND | ND | 3289 | 4 |
| Turkey | 16 | ND | ND | 1281 | 19 |
| Uganda | 30 | ND | ND | 1032 | 20 |
| Vietnam | 13 | ND | ND | 9 | 0 |

ND = “non-detect” (lead concentration was below the XRF’s lower detection limit)

Lead Concentration Results by Country
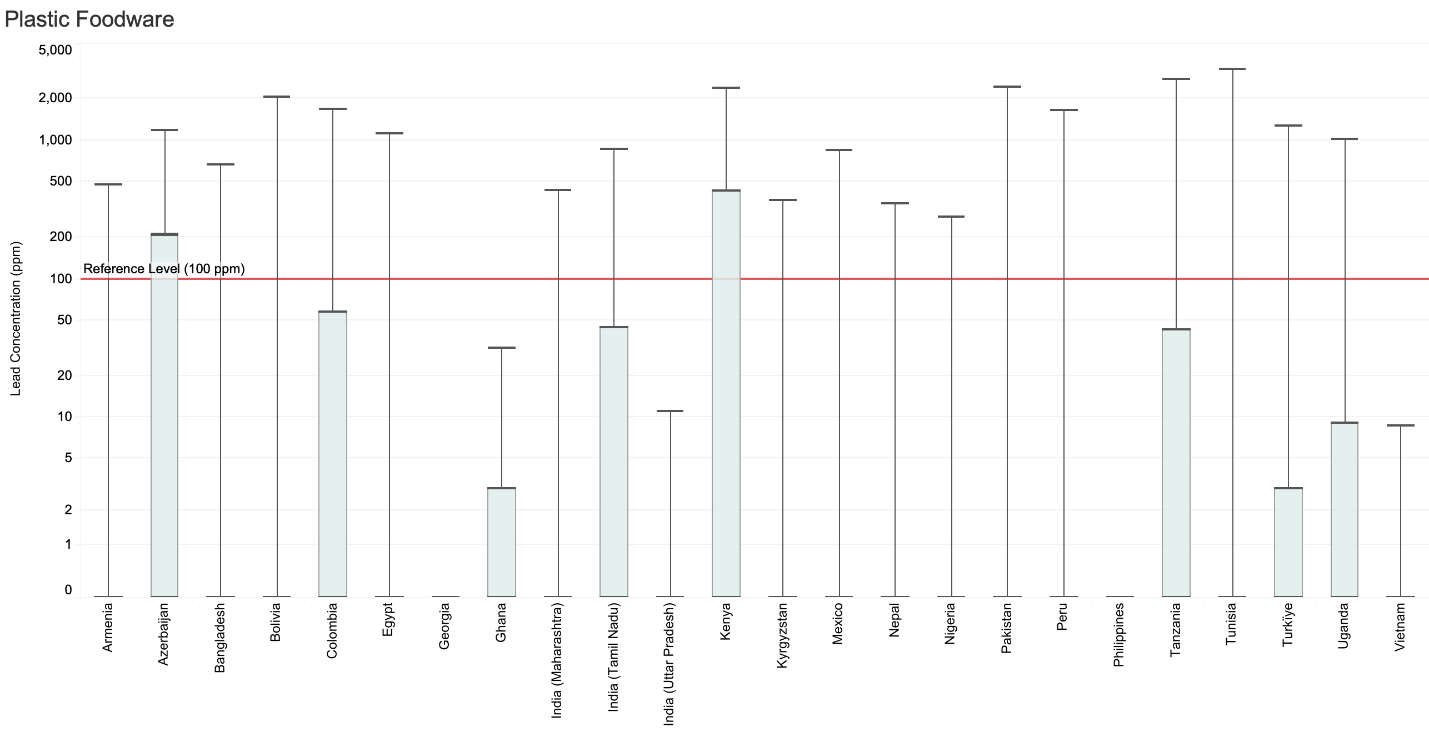


Cosmetics

Out of 812 cosmetics samples, 12% had lead levels exceeding the reference level of 2 ppm.

A wide array of cosmetic products were collected and divided into subcategories. Notably, elevated lead levels were identified across nearly all of the subcategories. Furthermore, cosmetics with elevated lead levels were found in 21 of the 25 countries.

The two items with the highest lead concentration were both eyeliners, known as kajal or kohl, from Pakistan. These samples had concentrations of 637,600 ppm (64%) and 1,000,000 ppm (100%) lead as assessed by XRF, with lower but still significant concentration (29% and 32%) reported by confirmatory laboratory testing. In some cultures, kajal/kohl is applied to infants and children. Unfortunately, the item with the third highest lead concentration of lead (128,400 ppm) was face paint intended specifically for children.

Among the samples with elevated lead levels, the most common item was nail polish (29 items), which had a maximum lead concentration of 6,751 ppm, followed by lipstick (15 items, maximum lead concentration of 42,350 ppm), and eyeshadow (13 items, maximum lead concentration of 974 ppm). As noted above and in the Quality Control section, some deviations were observed between the XRF and lab-based measurements of lead concentration at the highest concentrations among the cosmetics. Nevertheless, at such extreme concentrations, the risk is still significant even with a wide margin of error.

In addition to kajal/kohl, elevated lead levels were found in other traditional products, including henna and kumkum (a red powder made of turmeric and other ingredients and used for social and religious purposes in India).

Finally, it is notable that lead levels above the reference level were also found in a variety of conventional cosmetics, such as nail polish, lipstick, and eyeshadow as previously described, as well as face powder, mascara, eyeliner, liquid foundation/concealer, and hair products.

Readers should note that the reference level is near the XRF’s limit of detection for powders, and thus it is possible that some samples had a reading of “non-detect”, but actually exceeded 2 ppm.

Summary of Cosmetics Results by Country

| **Country Name** | **# of Samples** | **Min Value (ppm)** | **Median Value 9ppm)** | **Max Value (ppm)** | **% Above Reference** |
| --- | --- | --- | --- | --- | --- |
| Armenia | 29 | ND | ND | 174 | 7 |
| Azerbaijan | 10 | ND | ND | 8 | 10 |
| Bangladesh | 32 | ND | ND | 186 | 6 |
| Bolivia | 24 | ND | ND | 693 | 46 |
| Colombia | 39 | ND | ND | 6751 | 10 |
| Egypt | 19 | ND | ND | 13700 | 42 |
| Georgia | 27 | ND | ND | ND | 0 |
| Ghana | 28 | ND | ND | 220 | 7 |
| India - Maharashtra | 69 | ND | ND | 60 | 3 |
| India - Tamil Nadu | 32 | ND | ND | 231 | 9 |
| India - Uttar Pradesh | 44 | ND | ND | ND | 0 |
| Indonesia | 36 | ND | ND | 12 | 33 |
| Kazakhstan | 4 | ND | ND | ND | 0 |
| Kenya | 32 | ND | ND | 6 | 6 |
| Kyrgyzstan | 32 | ND | ND | 7 | 16 |
| Mexico | 29 | ND | ND | 50 | 7 |
| Nepal | 21 | ND | ND | ND | 0 |
| Nigeria | 50 | ND | ND | 1150 | 18 |
| Pakistan | 33 | ND | ND | 1000000 | 30 |
| Peru | 44 | ND | ND | 128400 | 9 |
| Philippines | 38 | ND | ND | 42350 | 13 |
| Tajikistan | 4 | ND | ND | ND | 0 |
| Tanzania | 30 | ND | ND | 52 | 3 |
| Tunisia | 27 | ND | ND | 712 | 11 |
| Turkey | 1 | 121 | 121 | 121 | 100 |
| Uganda | 48 | ND | ND | 3 | 2 |
| Vietnam | 30 | ND | ND | 68 | 23 |

ND = “non-detect” (lead concentration was below the XRF’s lower detection limit)

Lead Concentration Results by Country


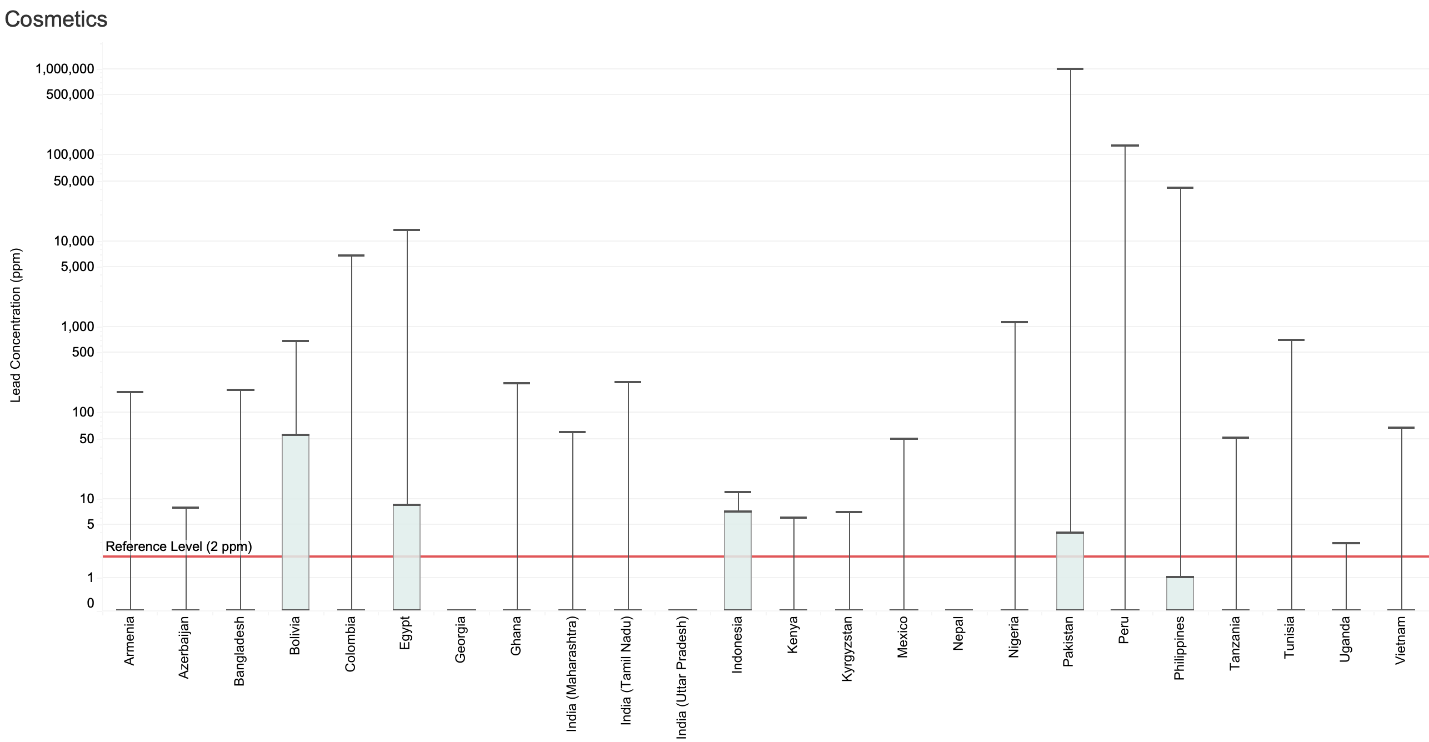


**Note**: Sample sizes below 5 are not displayed.

Toys

Out of 781 toy samples, 13% had lead levels exceeding the reference level of 100 ppm. Azerbaijan stands out with 69% of 26 samples exceeding the reference level.

This category encompasses a variety of hard toys, composed primarily of plastic items, but also including metal, wood or other materials. Some of these items were also found to have paint or coatings on them. In addition to the variety at the category level, many toys were heterogeneous, made from a combination of materials. We found many toys to contain internal electronic or metallic parts, which were responsible for some of the highest lead readings observed by XRF. Such readings may not necessarily best reflect the risk of that item, as the reference level relates to “accessible parts” to children. Also note that many of the toys collected and screened as part of the RMS were imported, which does not reduce the risk, but may influence interventions.

Summary of Toys Results by Country

| **Country Name** | **# of Samples** | **Min Value (ppm)** | **Median Value (ppm)** | **Max Value (ppm)** | **% Above Reference** |
| --- | --- | --- | --- | --- | --- |
| Armenia | 32 | ND | ND | 3125 | 3 |
| Azerbaijan | 26 | ND | 311 | 1175 | 69 |
| Bangladesh | 30 | ND | ND | 1814 | 13 |
| Bolivia | 18 | ND | ND | 1238 | 6 |
| Colombia | 34 | ND | ND | 455 | 12 |
| Egypt | 26 | ND | ND | 967 | 4 |
| Georgia | 38 | ND | ND | 376 | 3 |
| Ghana | 22 | ND | ND | 1533 | 14 |
| India - Maharashtra | 34 | ND | ND | 97300 | 21 |
| India - Tamil Nadu | 30 | ND | ND | 3250 | 23 |
| India - Uttar Pradesh | 38 | ND | ND | 4680 | 24 |
| Indonesia | 21 | ND | ND | 314 | 10 |
| Kazakhstan | 3 | ND | ND | 1138 | 33 |
| Kenya | 38 | ND | ND | 139 | 3 |
| Kyrgyzstan | 33 | ND | ND | 314 | 6 |
| Mexico | 27 | ND | ND | 1070 | 22 |
| Nepal | 10 | ND | ND | ND | 0 |
| Nigeria | 44 | ND | ND | 2292 | 16 |
| Pakistan | 30 | ND | ND | 1481 | 13 |
| Peru | 43 | ND | ND | 442 | 2 |
| Philippines | 36 | ND | ND | 2123 | 6 |
| Tajikistan | 5 | ND | ND | 34 | 0 |
| Tanzania | 30 | ND | ND | 698 | 10 |
| Tunisia | 25 | ND | ND | 176 | 4 |
| Turkey | 49 | ND | 22 | 4336 | 29 |
| Uganda | 29 | ND | ND | 81 | 0 |
| Vietnam | 30 | ND | ND | 298 | 7 |

ND = “non-detect” (lead concentration was below the XRF’s lower detection limit)

Lead Concentration Results by Country
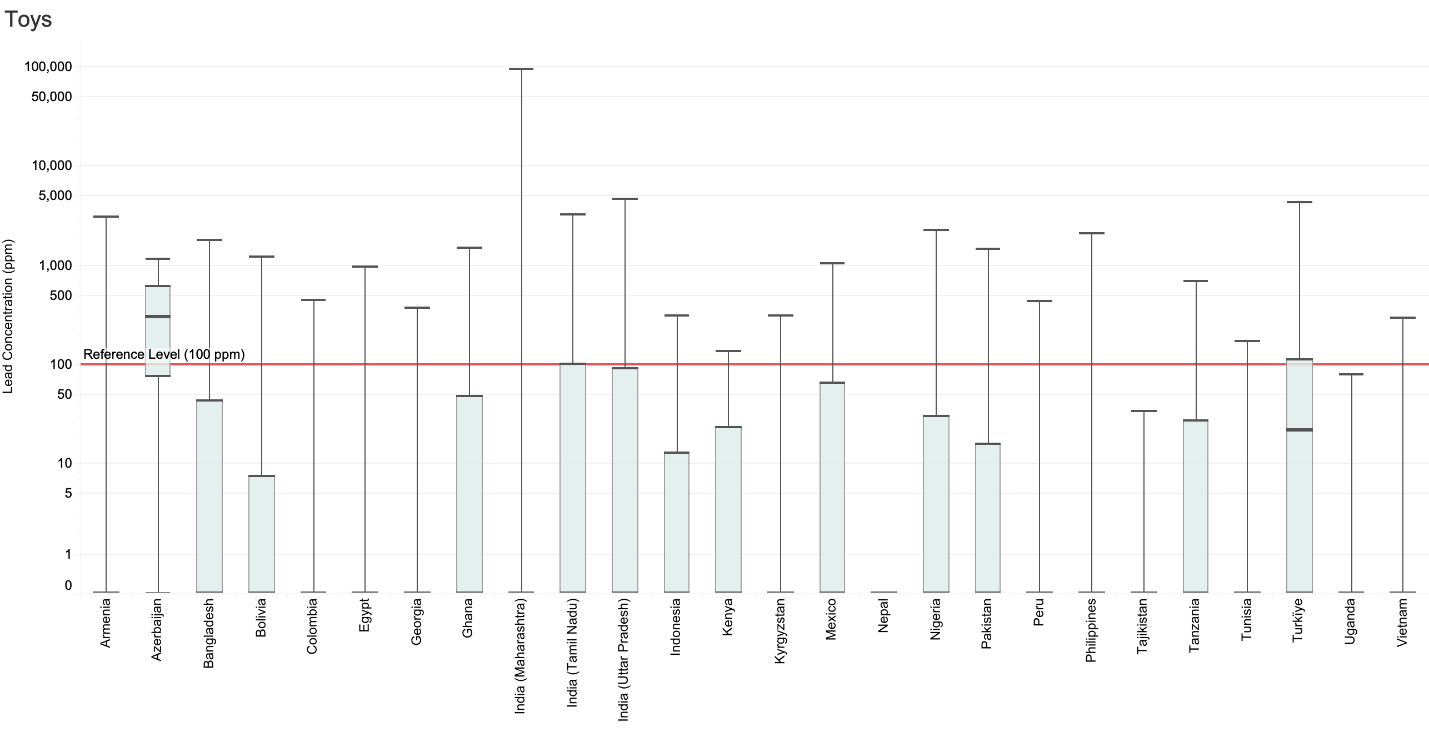


**Note**: Sample sizes below 5 are not displayed.

Paints Intended for Large Surfaces

Out of a total of 437 samples of paint intended for large surfaces, 41% showed lead levels exceeding the reference level of 90 ppm.

Pure Earth divided paint samples into two categories: paints intended for use on large surfaces, such as interior and exterior walls, and paints intended for crafts, art, and other specialty uses. This division was based on the recognition that exposure pathways may be different between wall paints, where exposure likely results from chipping paint that becomes dust, and specialty paints, where exposure may be more directly related to the application of the paint or use of the painted product (e.g., a toddler getting art paints in the mouth or mouthing a painted toy).

For all paints, we use a reference level of 90 ppm. The data below is for paints intended for large surfaces. Note that we were not able to classify all paint samples into these two categories, and thus not all paint samples are represented in the following two sections. Among the 102 unclassified paint samples, 47% exceeded the reference level. We also note that the protocol for testing paint was amended during the RMS to specify testing only dried paint samples as opposed to allowing analysis of wet samples. The following tables and charts include results of both wet and dry analyses.

In the table below, countries with binding regulations limiting lead concentrations in paints are highlighted with orange text.

Summary of Results for Paint Intended for Large Surfaces by Country

| **Country Name** | **# of Samples** | **Min Value (ppm)** | **Median Value (ppm)** | **Max Value (ppm)** | **% Above Reference** |
| --- | --- | --- | --- | --- | --- |
| Armenia | 7 | ND | ND | 6 | 0 |
| Azerbaijan | 18 | 600 | 2603 | 12400 | 100 |
| Bangladesh | 2 | ND | ND | ND | 0 |
| Bolivia | 5 | ND | ND | 27 | 0 |
| Colombia | 16 | ND | ND | 66200 | 31 |
| Egypt | 3 | ND | ND | ND | 0 |
| Georgia | 4 | ND | 376 | 22600 | 50 |
| Ghana | 1 | ND | ND | ND | 0 |
| India - Maharashtra | 27 | ND | ND | 164000 | 19 |
| India - Tamil Nadu | 7 | ND | 2356 | 13400 | 57 |
| India - Uttar Pradesh | 31 | ND | ND | 33200 | 42 |
| Indonesia | 31 | 1 | 3142 | 51400 | 97 |
| Kenya | 25 | ND | 7 | 7788 | 36 |
| Kyrgyzstan | 33 | ND | 10 | 890 | 33 |
| Mexico | 15 | 3 | 53700 | 807309* | 93 |
| Nepal | 20 | ND | ND | ND | 0 |
| Nigeria | 29 | ND | 494 | 20700 | 76 |
| Pakistan | 20 | ND | ND | 7370 | 35 |
| Peru | 10 | ND | ND | 2822 | 10 |
| Philippines | 32 | ND | ND | 41801 | 16 |
| Tanzania | 28 | ND | ND | 866 | 7 |
| Tunisia | 14 | ND | 286 | 72000 | 50 |
| Turkey | 10 | ND | 3937 | 11200 | 70 |
| Uganda | 32 | ND | ND | 12600 | 16 |
| Vietnam | 17 | ND | 777 | 25505 | 59 |

Orange text indicates countries with legally binding regulations on lead concentrations in paint according to information submitted to the [WHO Global Health Observatory](https://www.who.int/data/gho/data/themes/topics/indicator-groups/legally-binding-controls-on-lead-paint). Note: information regarding relevant regulations was not available from this source for Bolivia or Indonesia.

ND = “non-detect” (lead concentration was below the XRF’s lower detection limit)

* This value is unusually high. We recorded four XRF readings in this range from four different paint samples from the same brand of paint purchased from a market in Mexico. We have confirmed that these are paint samples, not pigments, but the samples have not been subjected to confirmatory lab analysis.

Lead Concentration Results by Country


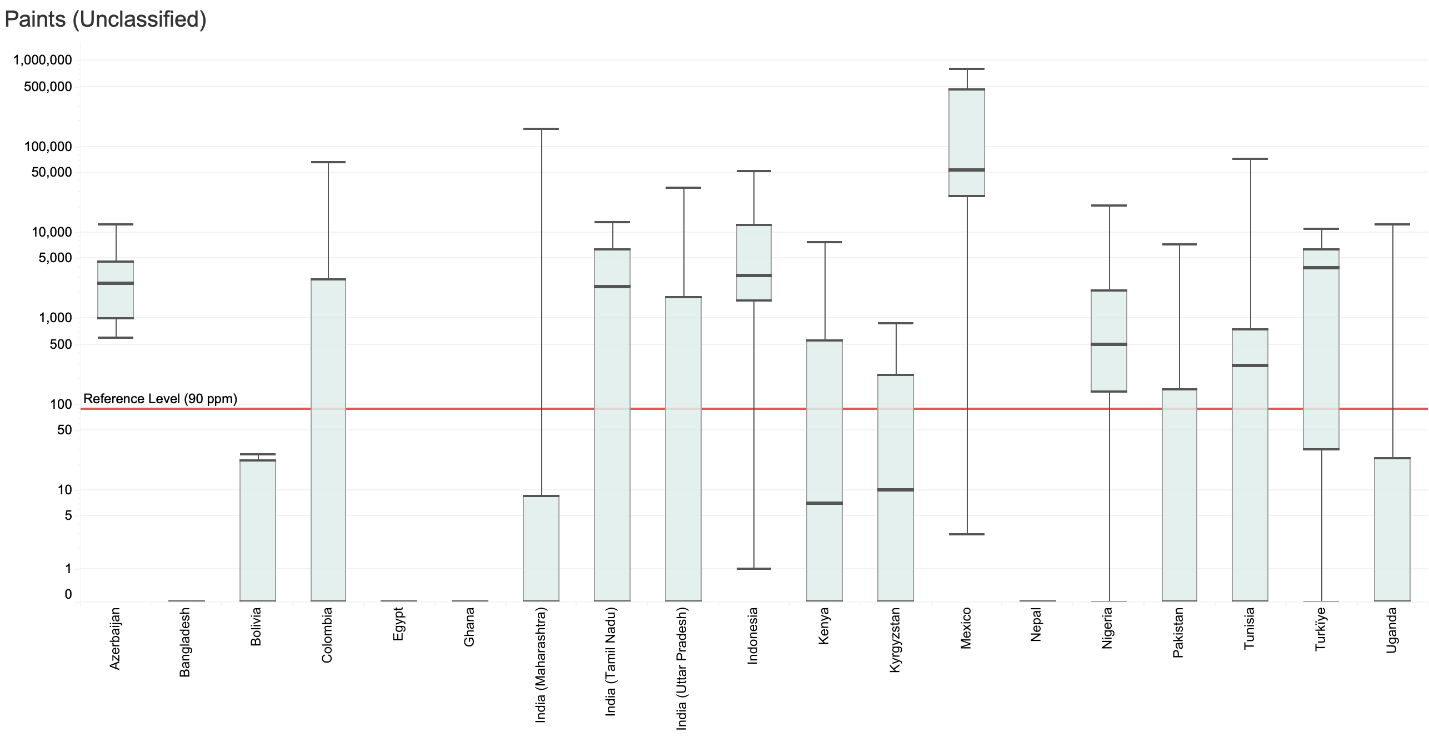


**Note**: Sample sizes below 5 are not displayed.

Paint Intended for Crafts, Art, and Specialty Uses

Out of a total of 70 samples of paint intended for crafts, art, and other specialty uses, 11% showed lead levels exceeding the reference level of 90 ppm. In the table below, countries with binding regulations limiting lead concentrations in paints are highlighted with orange text.

Summary of Results for Paint Intended for Crafts, Arts and Specialty Uses by Country

| **Country Name** | **# of Samples** | **Min Value (ppm)** | **Median Value (ppm)** | **Max Value (ppm)** | **% Above Reference** |
| --- | --- | --- | --- | --- | --- |
| Armenia | 4 | ND | 3 | 19 | 0 |
| Bangladesh | 2 | ND | 1903 | 3805 | 50 |
| Colombia | 9 | ND | ND | 93500 | 11 |
| Georgia | 14 | ND | ND | 4449 | 7 |
| Ghana | 3 | ND | ND | ND | 0 |
| India - Maharashtra | 12 | ND | ND | 1616 | 17 |
| Peru | 17 | ND | ND | 32 | 0 |
| Philippines | 3 | ND | ND | ND | 0 |
| Vietnam | 6 | ND | 612 | 7296 | 50 |

Orange text indicates countries with legally binding regulations on lead concentrations in paint according to information submitted to the [WHO Global Health Observatory](https://www.who.int/data/gho/data/themes/topics/indicator-groups/legally-binding-controls-on-lead-paint).

ND = “non-detect” (lead concentration was below the XRF’s lower detection limit)

Lead Concentration Results by Country
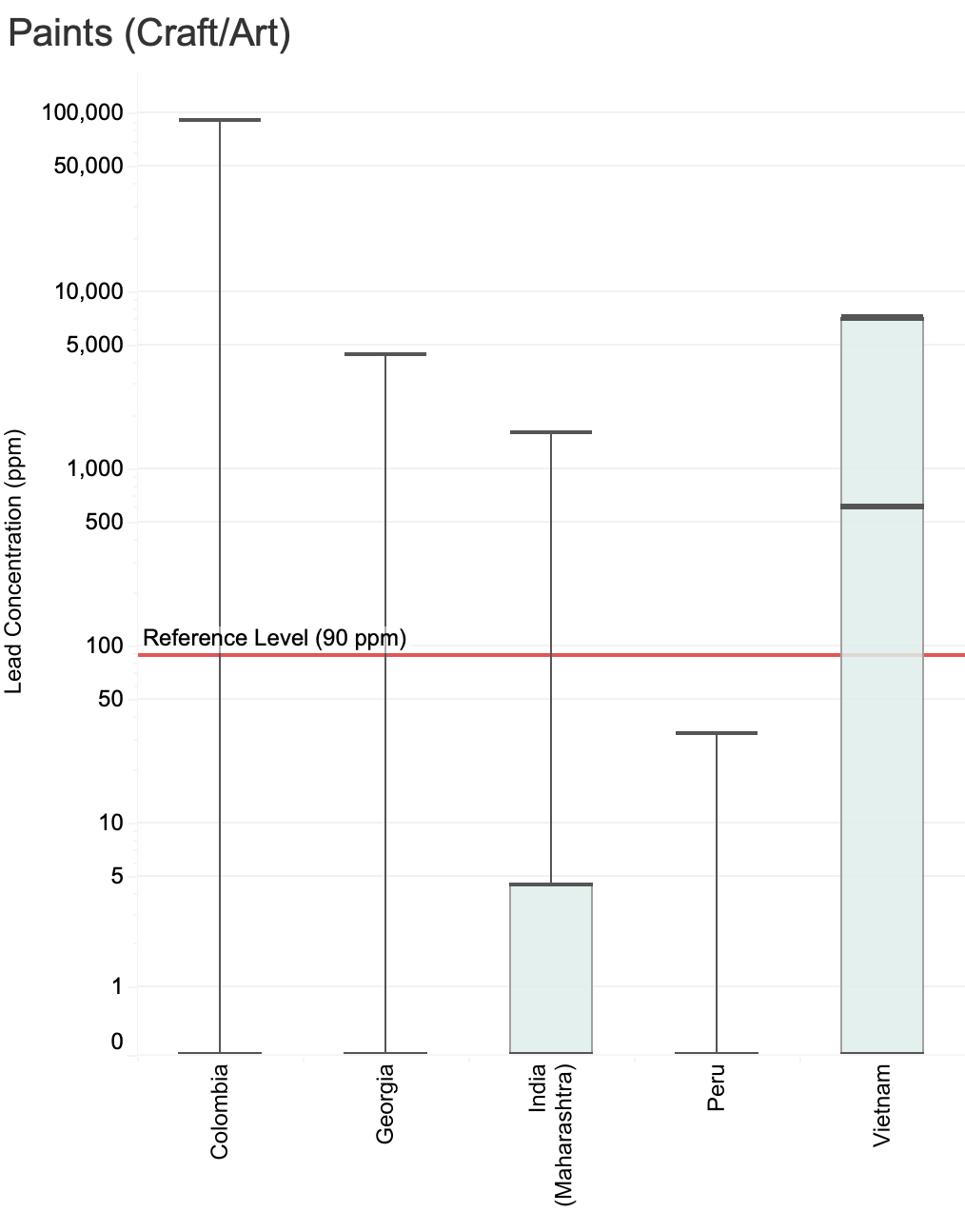


**Note**: Sample sizes below 5 are not displayed.

Spices

Out of 1,084 spice samples, 2% showed lead levels exceeding the reference level of 2 ppm.

Previous [studies](https://journals.lww.com/jphmp/fulltext/2019/01001/a_spoonful_of_lead__a_10_year_look_at_spices_as_a.11.aspx) have identified elevated lead levels in certain spices from countries around the Mediterranean, the Caucasus, and South Asia, among other regions.^^[[4]](#footnote-4)^^ In several countries, prior programs have confirmed that elevated lead concentrations were the result of producers adding lead-based pigments to spices to make their colors brighter.^^[[5]](#footnote-5)^,^[[6]](#footnote-6)^^ This has been a well-publicized issue for [turmeric](https://www.sciencedirect.com/science/article/pii/S0013935119305195) in particular, but has also been documented in other yellow, orange, and red spices.^^[[7]](#footnote-7)^^

The RMS was not designed to focus specifically on countries known to have contaminated spices, nor to focus solely on the types of spices that have been identified as more often contaminated. Rather, the RMS includes a broad range of spice types from countries that were selected based on product-agnostic criteria. As a result, the findings generally show low levels of lead in spices.

However, the RMS results should not cause readers to conclude that spices are not an important lead exposure source. There are, in fact, several reasons why it is both important and a good use of resources to focus on spices in certain regions. First, other recent assessments that have focused on commonly contaminated spices in “hotspot” regions have found a high prevalence of elevated lead levels among certain spices, particularly in North and Northeast India. Second, lead levels in spices have been successfully reduced through efforts to improve consumer and producer awareness, monitoring, supply chain tracking, and regulatory enforcement. Recent interventions in Bangladesh and Georgia, in particular, have resulted in dramatic declines in lead levels among certain targeted spices. Third, the intentional introduction of lead into spices is completely unnecessary, and eliminating the practice does not require significant changes to farming or production practices. Finally, spices represent an exposure source that can impact incredibly large populations, and thus programs to reduce lead levels in spices can have considerable impacts and returns on investments.

Readers should note that the minimum detection level for the XRF is often between 2-4 ppm for spices, and thus it is possible that some samples had a reading of “non-detect” but actually exceeded the reference level of 2 ppm. Readers should also note the very small sample size in Tajikistan. Field XRF results of spice samples from Tajikistan and Kazakhstan were expunged by the Quality Control Team, and thus the results below represent a small number of samples that were tested in accredited labs. Lastly, there is some uncertainty in the levels of lead in spices from Pakistan, as laboratory results were lower than those from the XRF. This is discussed in the Quality Control section.

Summary of Spices Results by Country

| **Country Name** | **# of Samples** | **Min Value (ppm)** | **Median Value (ppm)** | **Max Value (ppm)** | **% Above Reference** |
| --- | --- | --- | --- | --- | --- |
| Armenia | 48 | ND | ND | 12 | 4 |
| Azerbaijan | 4 | ND | ND | ND | 0 |
| Bangladesh | 46 | ND | ND | 4 | 7 |
| Bolivia | 61 | ND | ND | ND | 0 |
| Colombia | 54 | ND | ND | 19 | 2 |
| Egypt | 59 | ND | ND | 3 | 2 |
| Georgia | 45 | ND | ND | ND | 0 |
| Ghana | 47 | ND | ND | ND | 0 |
| India - Maharashtra | 50 | ND | ND | ND | 0 |
| India - Tamil Nadu | 44 | ND | ND | ND | 0 |
| India - Uttar Pradesh | 41 | ND | ND | 622 | 12 |
| Indonesia | 34 | ND | ND | ND | 0 |
| Kazakhstan | 8 | ND | ND | ND | 0 |
| Kenya | 41 | ND | ND | ND | 0 |
| Kyrgyzstan | 48 | ND | ND | ND | 0 |
| Mexico | 29 | ND | ND | 10 | 3 |
| Nepal | 53 | ND | ND | ND | 0 |
| Nigeria | 67 | ND | ND | ND | 0 |
| Pakistan | 46 | ND | ND | 160 | 9 |
| Peru | 43 | ND | ND | 7 | 2 |
| Philippines | 55 | ND | ND | 2 | 0 |
| Tajikistan | 5 | ND | ND | 381 | 60 |
| Tanzania | 45 | ND | ND | 21 | 2 |
| Tunisia | 36 | ND | ND | ND | 0 |
| Turkey | 4 | ND | ND | 4 | 25 |
| Uganda | 40 | ND | ND | ND | 0 |
| Vietnam | 31 | ND | ND | 9 | 3 |

ND = “non-detect” (lead concentration was below the XRF’s lower detection limit)

Lead Concentration Results by Country**
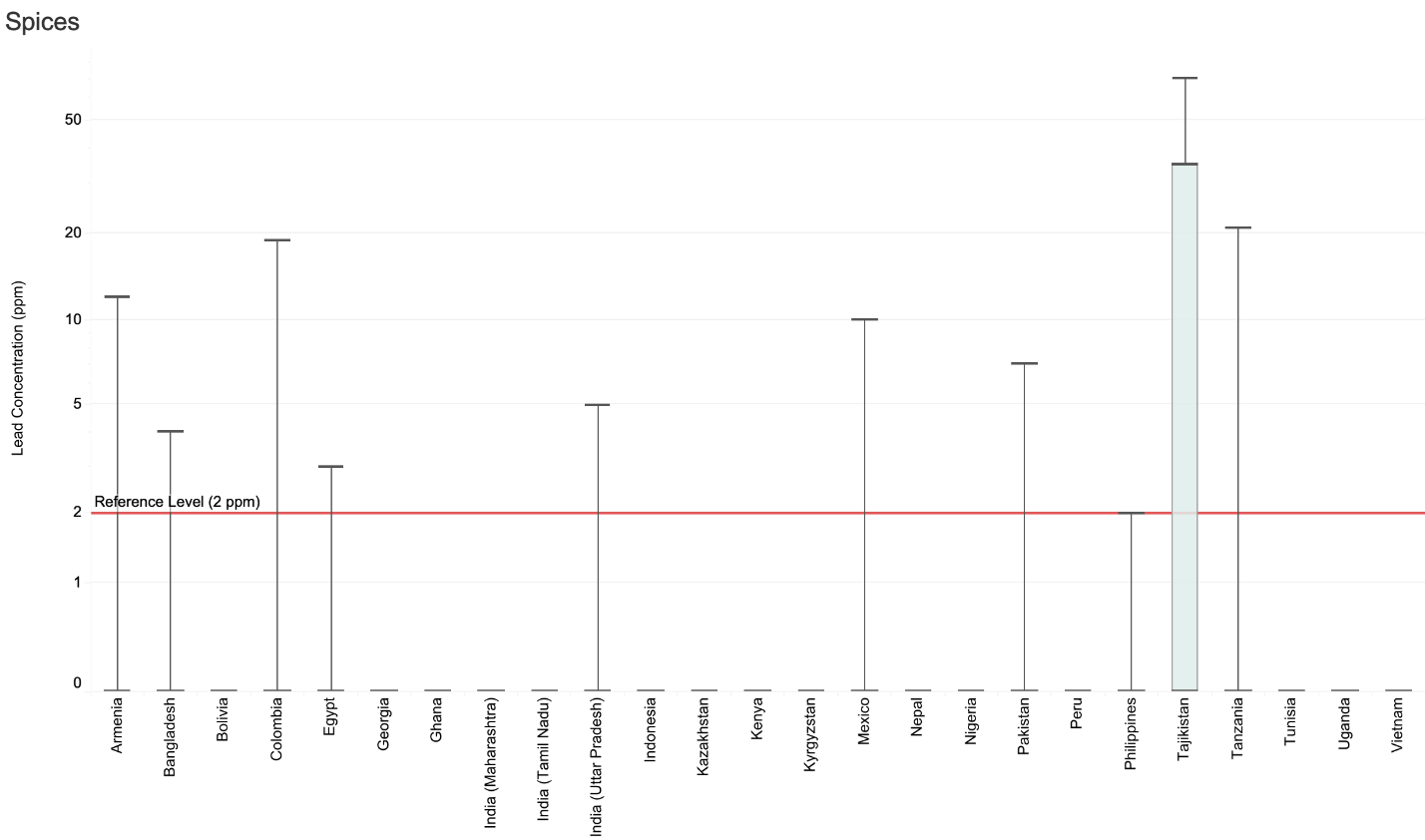
**

**Note**: Sample sizes below 5 are not displayed.

The 24 spice samples found to exceed the reference level were from 12 countries, with the highest number from the state of Uttar Pradesh in India (5), followed by Pakistan (4), Bangladesh (3), Tajikistan (3), Armenia (2), Turkey (1), Egypt (1), Tanzania (1), Vietnam (1), Mexico (1), Colombia (1) and Peru (1), representing a high level of geographic diversity.

Turmeric was the most common spice among those with elevated lead levels, representing 9 of the 24 samples. Furthermore, out of the 7 samples with the highest lead concentrations, 6 were turmeric samples. The maximum concentration of lead in turmeric was found to be 622 ppm, more than 300 times the reference level. Levels this high point to likely adulteration with a lead-based pigment.

Other spices identified as having elevated lead levels were certain spice mixes such as garam masala, curry powder, and mole, as well as cardamom, achiote (annatto), coriander, caraway, ginger, salt, chili, paprika, cinnamon, and pepper.

Sweets

Out of 111 samples of sweets, 3% showed lead levels exceeding the reference level of 0.1 ppm. Readers should note that the reference level for sweets is below the XRF’s limit of detection. Therefore, it is possible that samples had a reading of “non-detect” but actually exceeded the reference level.

Summary of Sweets Results by Country

| **Country Name** | **# of Samples** | **Min Value (ppm)** | **Median Value (ppm)** | **Max Value (ppm)** | **% Above Reference** |
| --- | --- | --- | --- | --- | --- |
| Tanzania | 30 | ND | ND | 4 | 3 |
| Bolivia | 2 | ND | ND | ND | 0 |
| Colombia | 11 | ND | ND | ND | 0 |
| Mexico | 48 | ND | ND | 5 | 4 |
| Pakistan | 20 | ND | ND | ND | 0 |

ND = “non-detect” (lead concentration was below the XRF’s lower detection limit)

Lead Concentration Results by Country


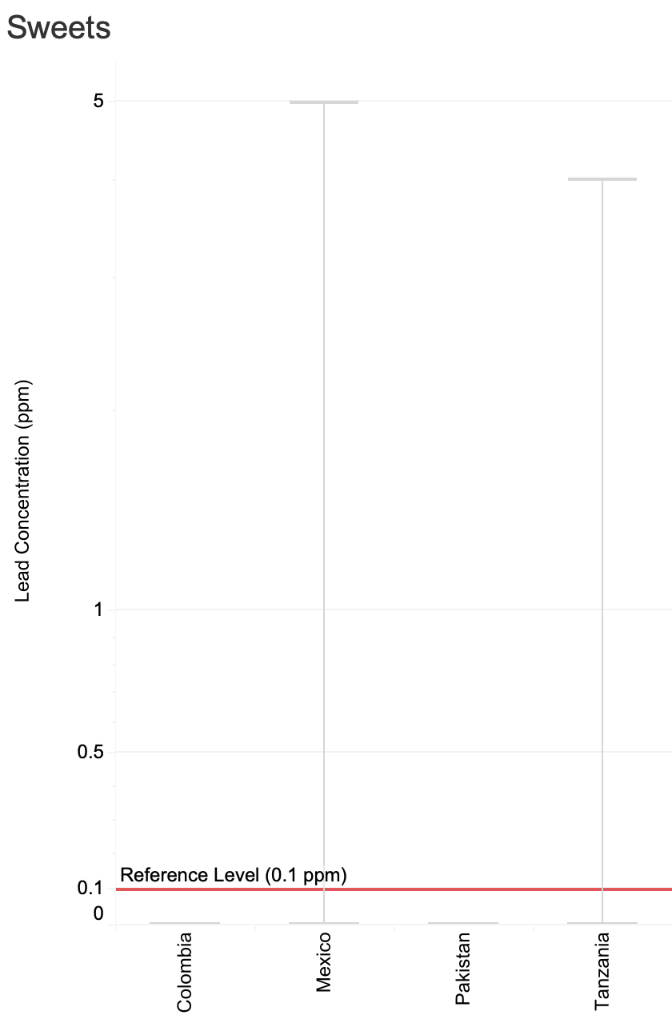


**Note**: Sample sizes below 5 are not displayed.

Staple Dry Foods

Out of 364 samples of dry foods that local RMS Investigators felt were common enough to consider as “staples,” 1% showed lead levels exceeding the reference level of 0.2 ppm.

Readers should note that the reference level for staple dry foods is below the XRF’s limit of detection. Therefore, it is possible that samples had a reading of “non-detect,” but actually exceeded the reference level.

Summary of Staple Dry Foods Results by Country

| **Country Name** | **# of Samples** | **Min Value (ppm)** | **Median Value (ppm)** | **Max Value (ppm)** | **% Above Reference** |
| --- | --- | --- | --- | --- | --- |
| Armenia | 9 | ND | ND | 3 | 11 |
| Georgia | 5 | ND | ND | ND | 0 |
| Kazakhstan | 3 | ND | ND | ND | 0 |
| Kyrgyzstan | 14 | ND | ND | ND | 0 |
| Tajikistan | 3 | ND | ND | ND | 0 |
| Kenya | 8 | ND | ND | ND | 0 |
| Tanzania | 9 | ND | ND | ND | 0 |
| Uganda | 17 | ND | ND | 3 | 6 |
| Bolivia | 6 | ND | ND | ND | 0 |
| Colombia | 9 | ND | ND | ND | 0 |
| Mexico | 11 | ND | ND | ND | 0 |
| Peru | 10 | ND | ND | ND | 0 |
| Egypt | 4 | ND | ND | ND | 0 |
| Tunisia | 18 | ND | ND | ND | 0 |
| Bangladesh | 12 | ND | ND | 8 | 17 |
| India - Maharashtra | 10 | ND | ND | ND | 0 |
| India - Tamil Nadu | 9 | ND | ND | ND | 0 |
| India - Uttar Pradesh | 3 | ND | ND | ND | 0 |
| Nepal | 16 | ND | ND | ND | 0 |
| Pakistan | 9 | ND | ND | ND | 0 |
| Indonesia | 9 | ND | ND | ND | 0 |
| Philippines | 49 | ND | ND | 17 | 2 |
| Vietnam | 9 | ND | ND | ND | 0 |
| Ghana | 29 | ND | ND | ND | 0 |
| Nigeria | 83 | ND | ND | ND | 0 |

ND = “non-detect” (lead concentration was below the XRF’s lower detection limit)

Lead Concentration Results by Country
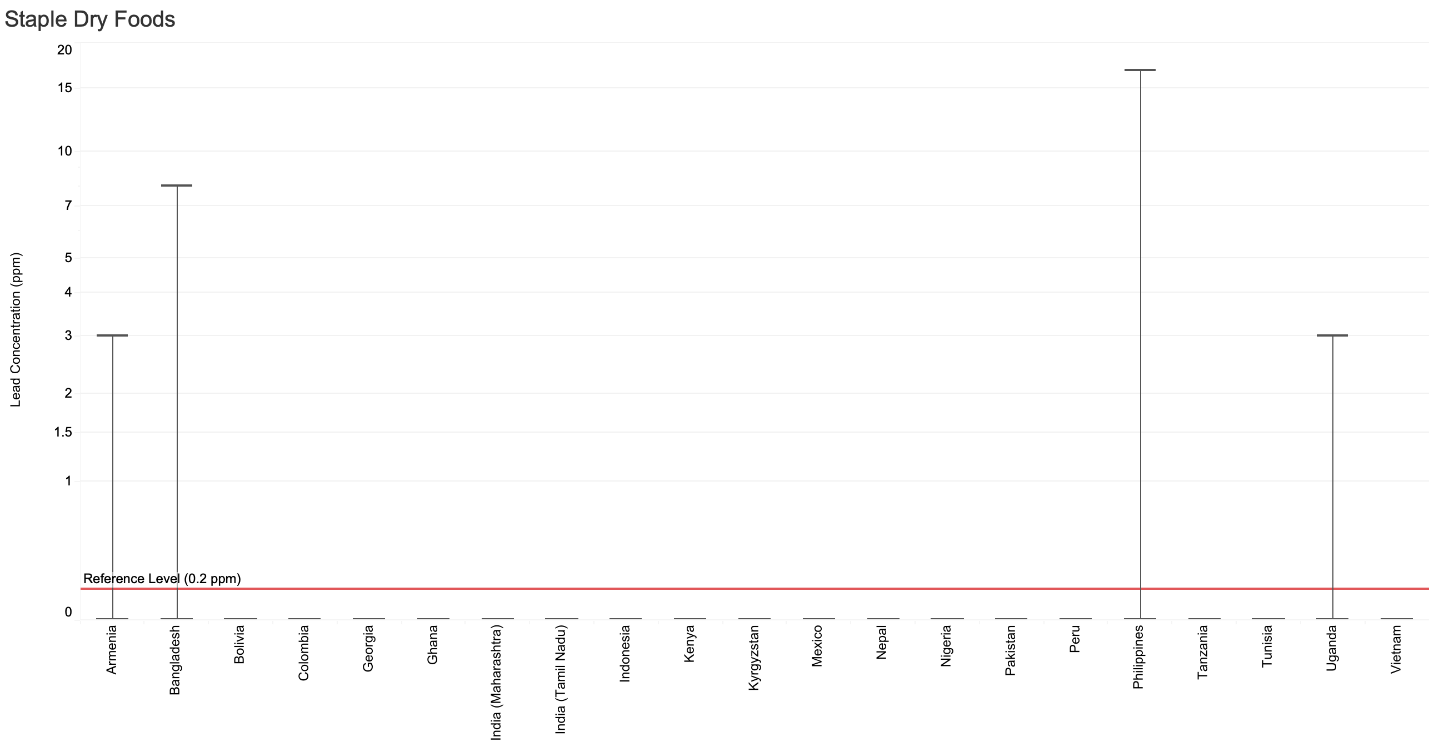


**Note**: Sample sizes below 5 are not displayed.

Herbal/Traditional Medicines

Out of 54 samples of herbal and traditional medicines, 4% showed lead levels exceeding the reference level of 10 ppm.

Summary of Herbal/Traditional Medicines Results by Country

| **Country Name** | **# of Samples** | **Min Value (ppm)** | **Median Value (ppm)** | **Max Value (ppm)** | **% Above Reference** |
| --- | --- | --- | --- | --- | --- |
| Uganda | 1 | 31 | 31 | 31 | 100 |
| Colombia | 8 | ND | ND | ND | 0 |
| Mexico | 4 | ND | ND | ND | 0 |
| Peru | 2 | ND | ND | ND | 0 |
| Egypt | 10 | ND | ND | ND | 0 |
| Tunisia | 6 | ND | ND | 19 | 17 |
| India - Maharashtra | 3 | ND | ND | ND | 0 |
| India - Uttar Pradesh | 4 | ND | ND | ND | 0 |
| Nepal | 9 | ND | ND | ND | 0 |
| Philippines | 2 | ND | ND | ND | 0 |
| Vietnam | 5 | ND | ND | ND | 0 |

ND = “non-detect” (lead concentration was below the XRF’s lower detection limit)

Lead Concentration Results by Country


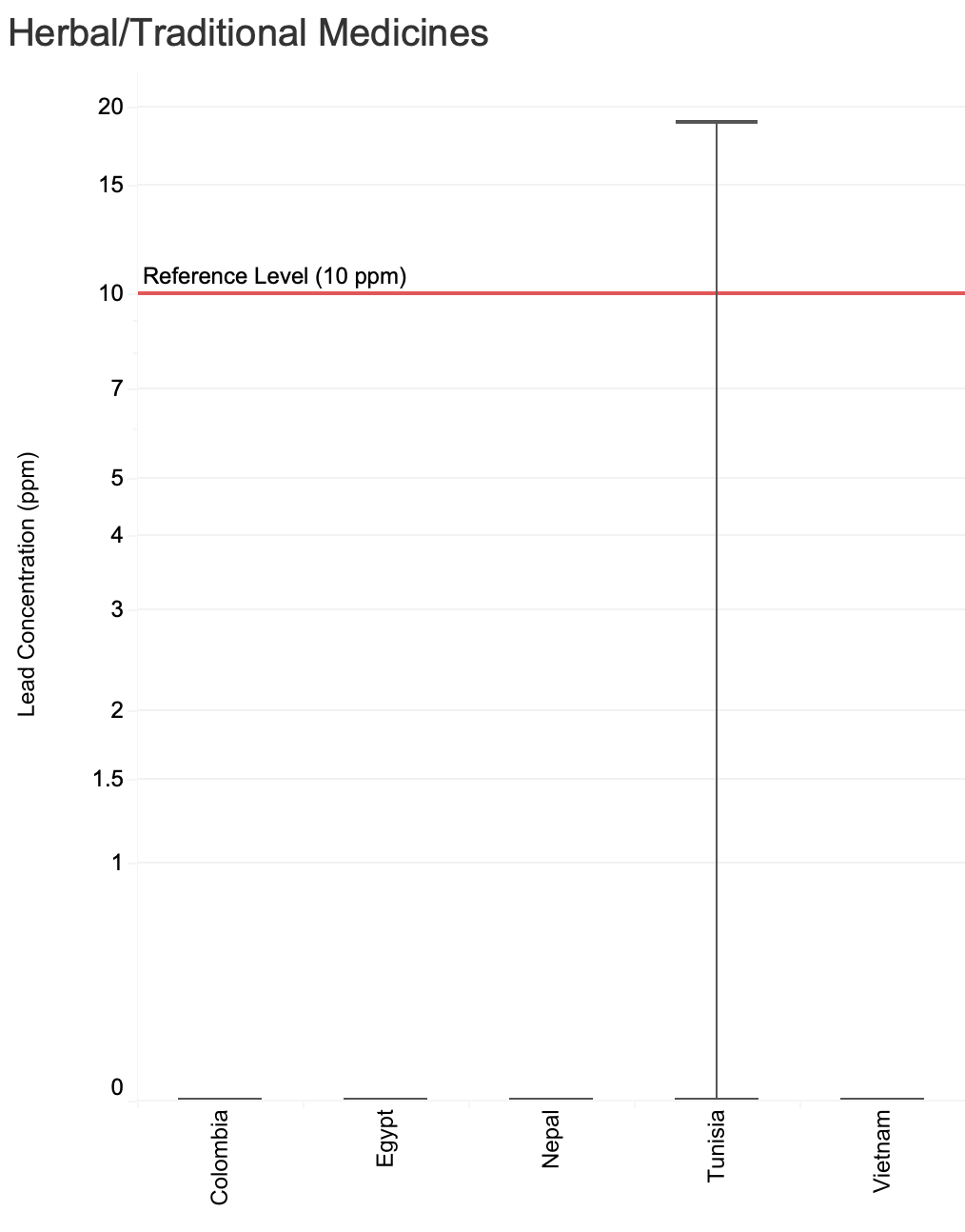


**Note**: Sample sizes below 5 are not displayed.

**Supplement D: Lead in Consumer Goods by Country**

Findings by Country

## Armenia

Pure Earth analyzed a total of 180 samples from Armenia, and of these, 7% exceeded the relevant reference levels. As with other countries, a high percentage of ceramic foodware exceeded the reference level (36%). Overall, samples from Armenia had comparatively lower lead levels than many other countries.

Summary of Results from Armenia in Order of % Exceeding Reference Levels

| **Item Category** | **# of Samples** | **Min Value (ppm)** | **Median (ppm)** | **Max Value (ppm)** | **% above reference** |
| --- | --- | --- | --- | --- | --- |
| Ceramic foodware | 11 | ND | 58 | 9280 | 36 |
| Staple dry food | 9 | ND | ND | 3 | 11 |
| Metallic foodware | 19 | ND | ND | 287 | 11 |
| Cosmetics | 29 | ND | ND | 174 | 7 |
| Plastic foodware | 18 | ND | ND | 478 | 6 |
| Spices | 48 | ND | ND | 12 | 4 |
| Toys | 32 | ND | ND | 3125 | 3 |
| Paint - unclassified | 3 | ND | ND | ND | 0 |
| Paint - craft/art | 4 | ND | 3 | 19 | 0 |
| Paint - large surfaces | 7 | ND | ND | 6 | 0 |

ND = “non-detect” (lead concentration was below the XRF’s lower detection limit)

Percentage of Samples from Armenia Below and Above the Reference Level


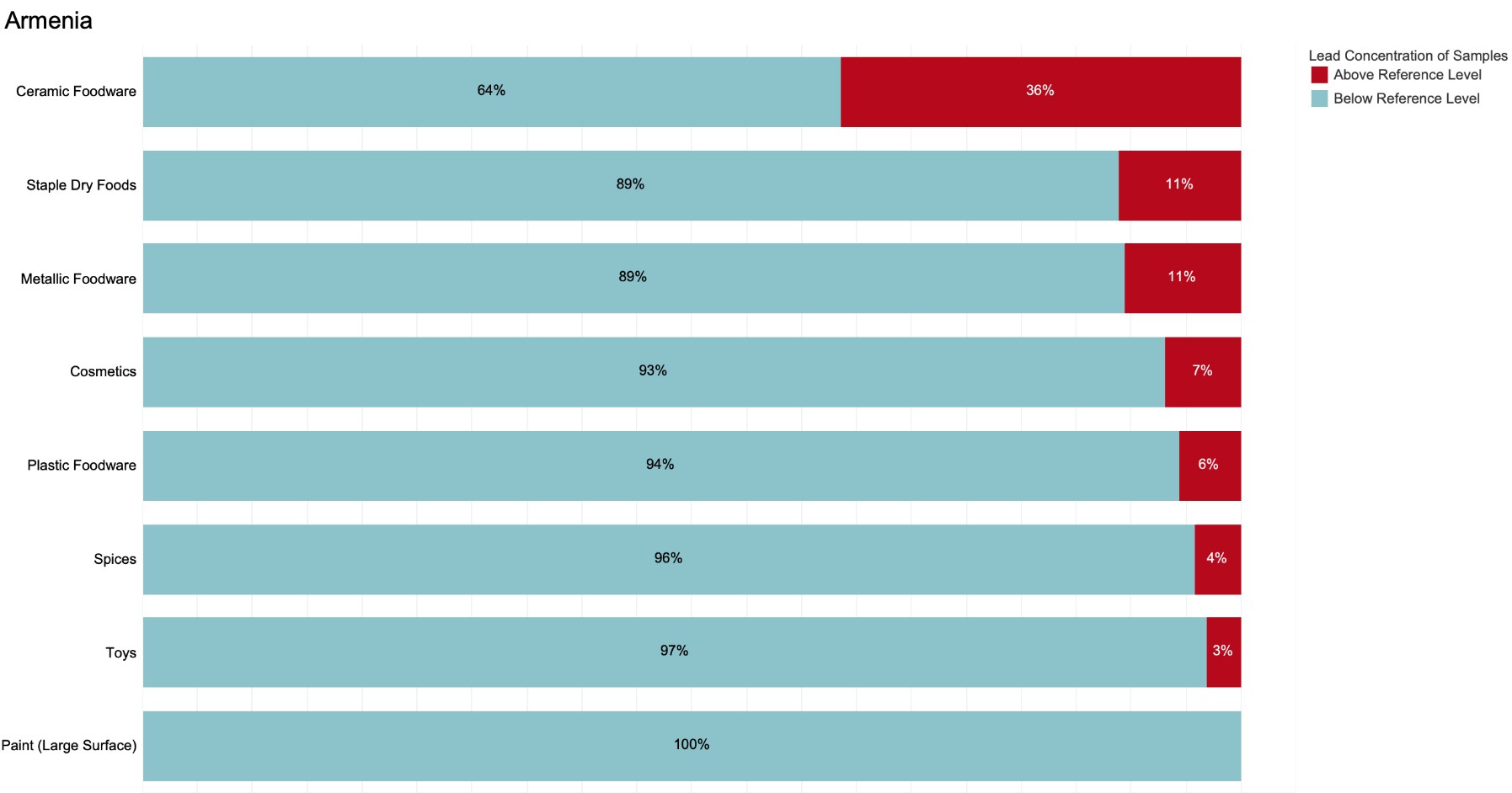


**Key:** Blue = percentage of samples below reference level. Red = percentage above reference level.

**Note**: Sample sizes below 5 are not displayed.

## Azerbaijan

Pure Earth analyzed a total of 92 samples from Azerbaijan, and of these, 68% exceeded the relevant reference levels. A comparatively high percentage of samples of foodware, toys, and paints exceeded the reference levels, but almost all categories had samples with elevated lead levels.

Summary of Results from Azerbaijan in Order of % Exceeding Reference Levels

| **Item category** | **# of Samples** | **Min Value (ppm)** | **Median** | **Max Value (ppm)** | **% above reference** |
| --- | --- | --- | --- | --- | --- |
| Ceramic foodware | 13 | 312 | 774 | 11400 | 100 |
| Paints | 18 | 600 | 2603 | 12400 | 100 |
| Toys | 26 | ND | 311 | 1175 | 69 |
| Metallic foodware | 16 | ND | 178 | 2342 | 63 |
| Plastic foodware | 5 | ND | 211 | 1196 | 60 |
| Cosmetics | 10 | ND | ND | 8 | 10 |
| Spices | 4 | ND | ND | ND | 0 |

ND = “non-detect” (lead concentration was below the XRF’s lower detection limit)

Percentage of Samples from Azerbaijan Below and Above the Reference Level


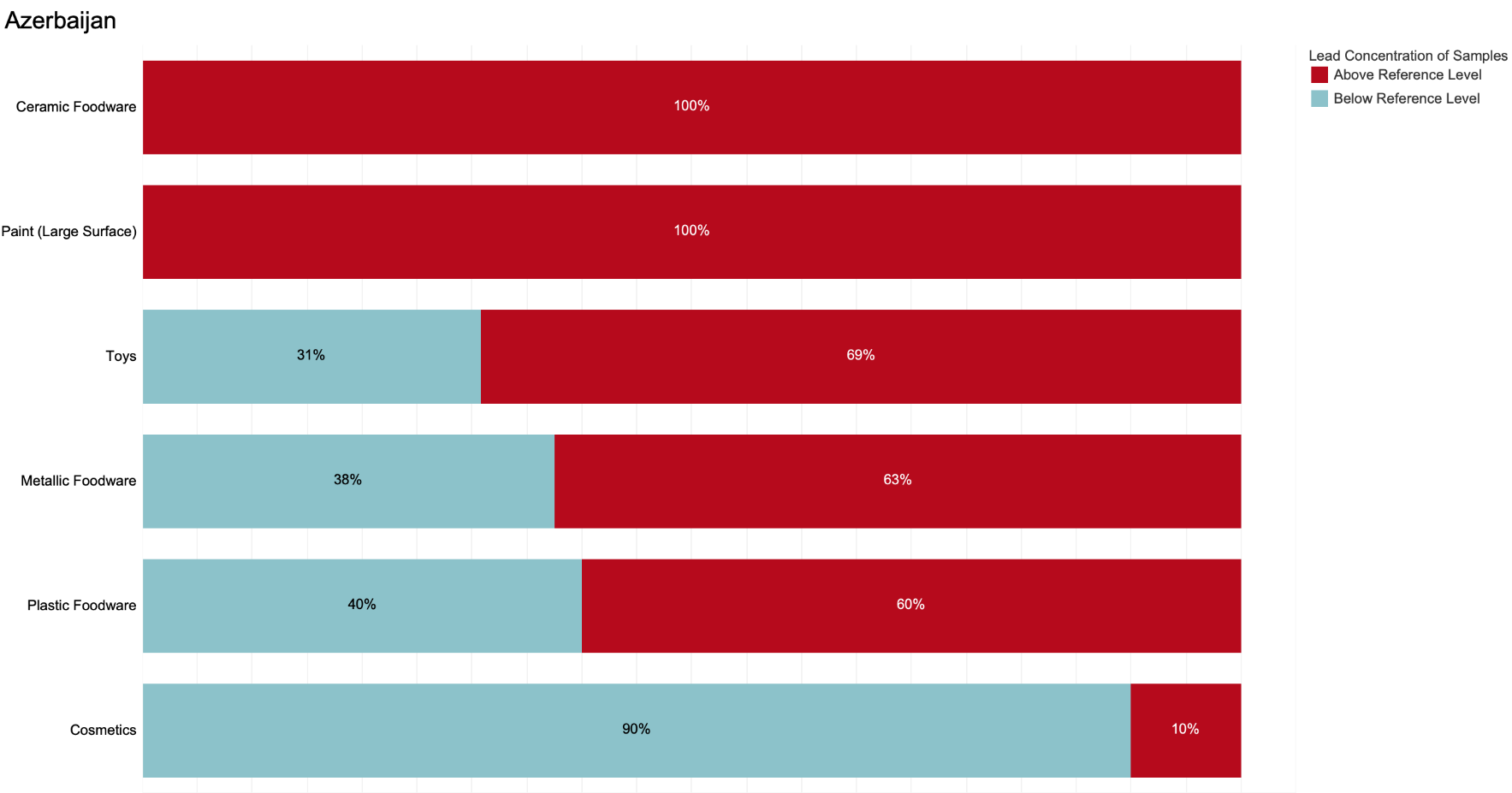


**Key:** Blue = percentage of samples below reference level. Red = percentage above reference level.

**Note**: Sample sizes below 5 are not displayed.

## Bangladesh

Pure Earth analyzed 197 samples from Bangladesh, and of these, 24% exceeded the relevant reference levels. Metal foodware, ceramic foodware, and paint stand out as product types that had a particularly high percentage of samples exceeding the reference levels.

Summary of Results from Bangladesh in Order of % Exceeding Reference Levels

| **Item category** | **# of Samples** | **Min Value (ppm)** | **Median** | **Max Value (ppm)** | **% Above Reference** |
| --- | --- | --- | --- | --- | --- |
| Metallic foodware | 27 | ND | 186 | 8186 | 59 |
| Paint - unclassified | 26 | ND | 345 | 31360 | 54 |
| Paint craft/art | 2 | ND | 1903 | 3805 | 50 |
| Ceramic foodware | 9 | ND | 22 | 4636 | 44 |
| Staple dry food | 12 | ND | ND | 8 | 17 |
| Toys | 30 | ND | ND | 1814 | 13 |
| Plastic foodware | 11 | ND | ND | 672 | 9 |
| Spices | 46 | ND | ND | 4 | 7 |
| Cosmetics | 32 | ND | ND | 186 | 6 |
| Paint - large surfaces | 2 | ND | ND | ND | 0 |

ND = “non-detect” (lead concentration was below the XRF’s lower detection limit)

Percentage of Samples from Bangladesh Below and Above the Reference Level


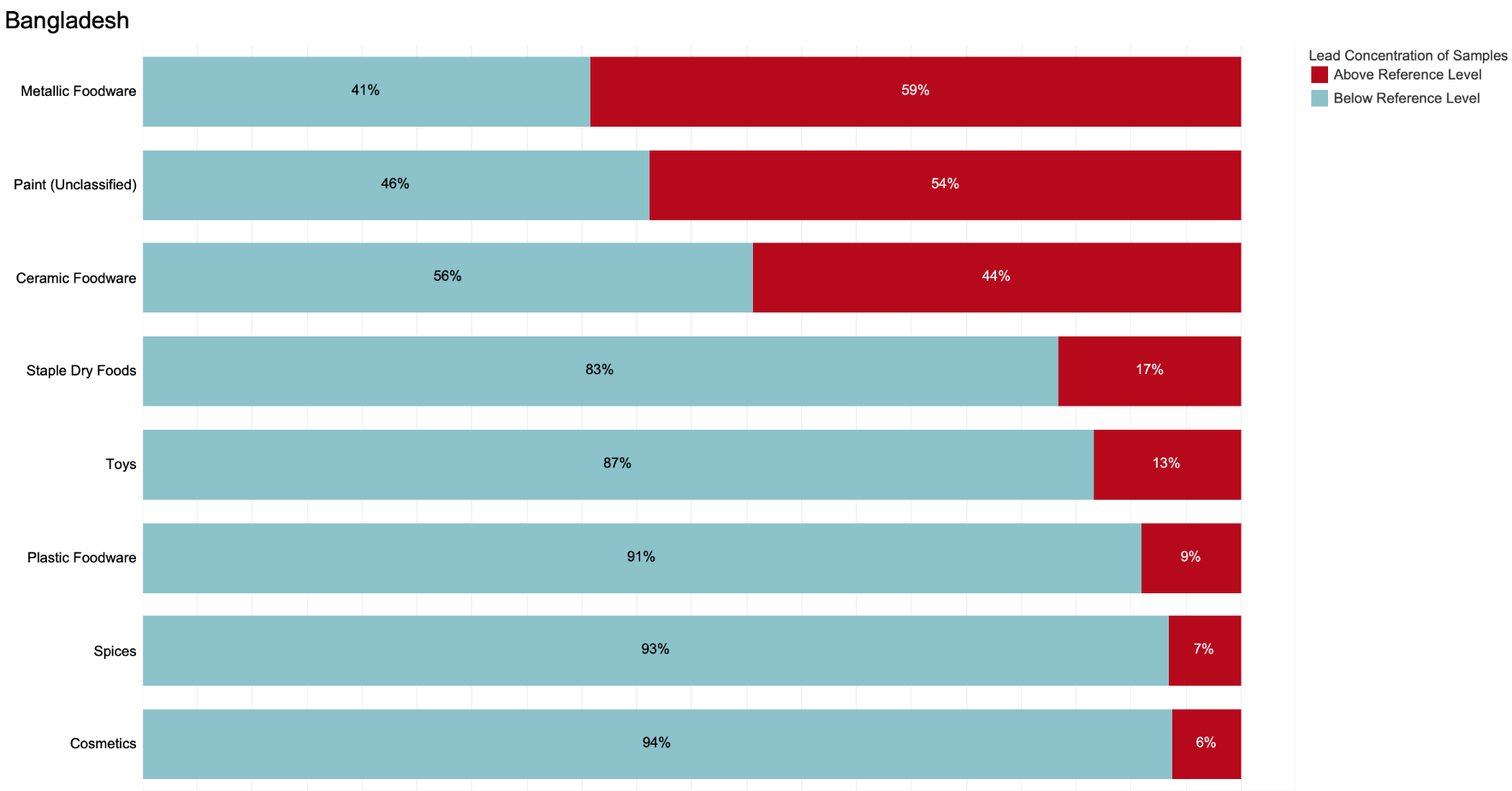


**Key:** Blue = percentage of samples below reference level. Red = percentage above reference level.

**Note**: Sample sizes below 5 are not displayed.

## Bolivia

Pure Earth analyzed a total of 153 samples from Bolivia, and of these, 18% exceeded the relevant reference levels. As in many countries, a high percentage of metal and ceramic foodware samples exceeded the relevant reference levels, but unlike many countries, nearly 50% of the 24 cosmetics samples exceeded the relevant reference level.

Summary of Results from Bolivia in Order of % Exceeding Reference Levels

| **Item Category** | **# of Samples** | **Min Value (ppm)** | **Median (ppm)** | **Max Value (ppm)** | **% Above Reference** |
| --- | --- | --- | --- | --- | --- |
| Ceramic foodware | 10 | 35 | 131853 | 397100 | 60 |
| Metallic foodware | 13 | ND | 164 | 2049 | 54 |
| Cosmetics | 24 | ND | ND | 693 | 46 |
| Plastic foodware | 14 | ND | ND | 2073 | 14 |
| Toys | 18 | ND | ND | 1238 | 6 |
| Paints | 5 | ND | ND | 27 | 0 |
| Spices | 61 | ND | ND | ND | 0 |
| Staple dry food | 6 | ND | ND | ND | 0 |
| Sweets | 2 | ND | ND | ND | 0 |

ND = “non-detect” (lead concentration was below the XRF’s lower detection limit)

Percentage of Samples from Bolivia Below and Above the Reference Level


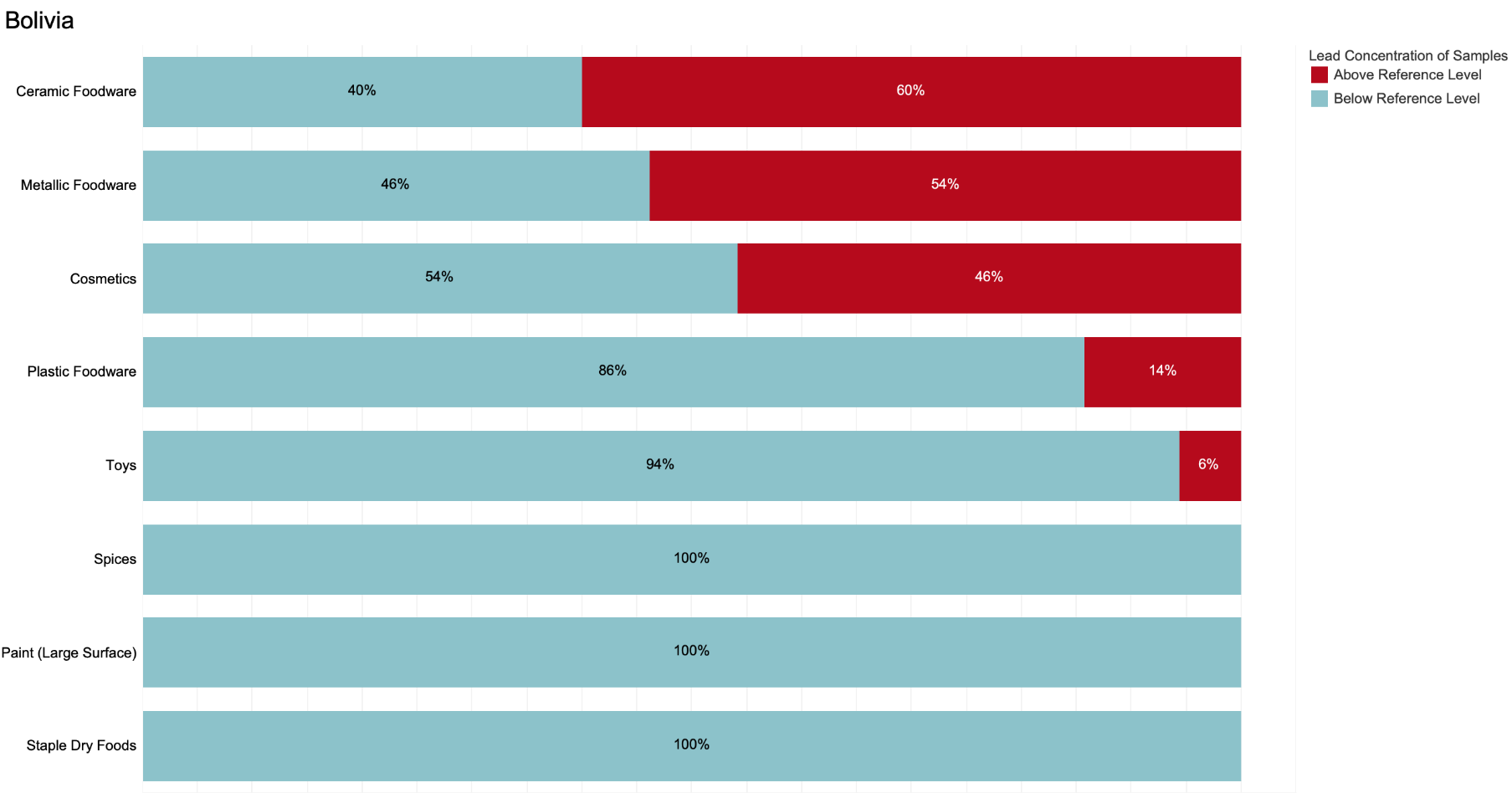


**Key:** Blue = percentage of samples below reference level. Red = percentage above reference level.

**Note**: Sample sizes below 5 are not displayed.

## Colombia

Pure Earth analyzed a total of 260 samples from Colombia, and of these, 18% exceeded the relevant reference levels. Metal foodware, ceramic foodware, and paint stand out as product types that had a particularly high percentage of samples exceeding the reference levels, but samples of cosmetics and toys also showed elevated lead levels.

Summary of Results from Colombia in Order of % Exceeding Reference Levels

| **Item Category** | **# of Samples** | **Min Value (ppm)** | **Median (ppm)** | **Max Value (ppm)** | **% Above Reference** |
| --- | --- | --- | --- | --- | --- |
| Paint - unclassified | 10 | ND | 3268 | 58700 | 60 |
| Ceramic foodware | 18 | ND | 237 | 29100 | 50 |
| Metallic foodware | 35 | ND | 51 | 2679 | 40 |
| Paints - large surfaces | 16 | ND | ND | 66200 | 31 |
| Plastic foodware | 17 | ND | ND | 1687 | 24 |
| Toys | 34 | ND | ND | 455 | 12 |
| Paint - craft/art | 9 | ND | ND | 93500 | 11 |
| Cosmetics | 39 | ND | ND | 6751 | 10 |
| Spices | 54 | ND | ND | 19 | 2 |
| Herbal/Trad Medicines | 8 | ND | ND | ND | 0 |
| Staple dry food | 9 | ND | ND | ND | 0 |
| Sweets | 11 | ND | ND | ND | 0 |

ND = “non-detect” (lead concentration was below the XRF’s lower detection limit)

Percentage of Samples from Colombia Below and Above the Reference Level


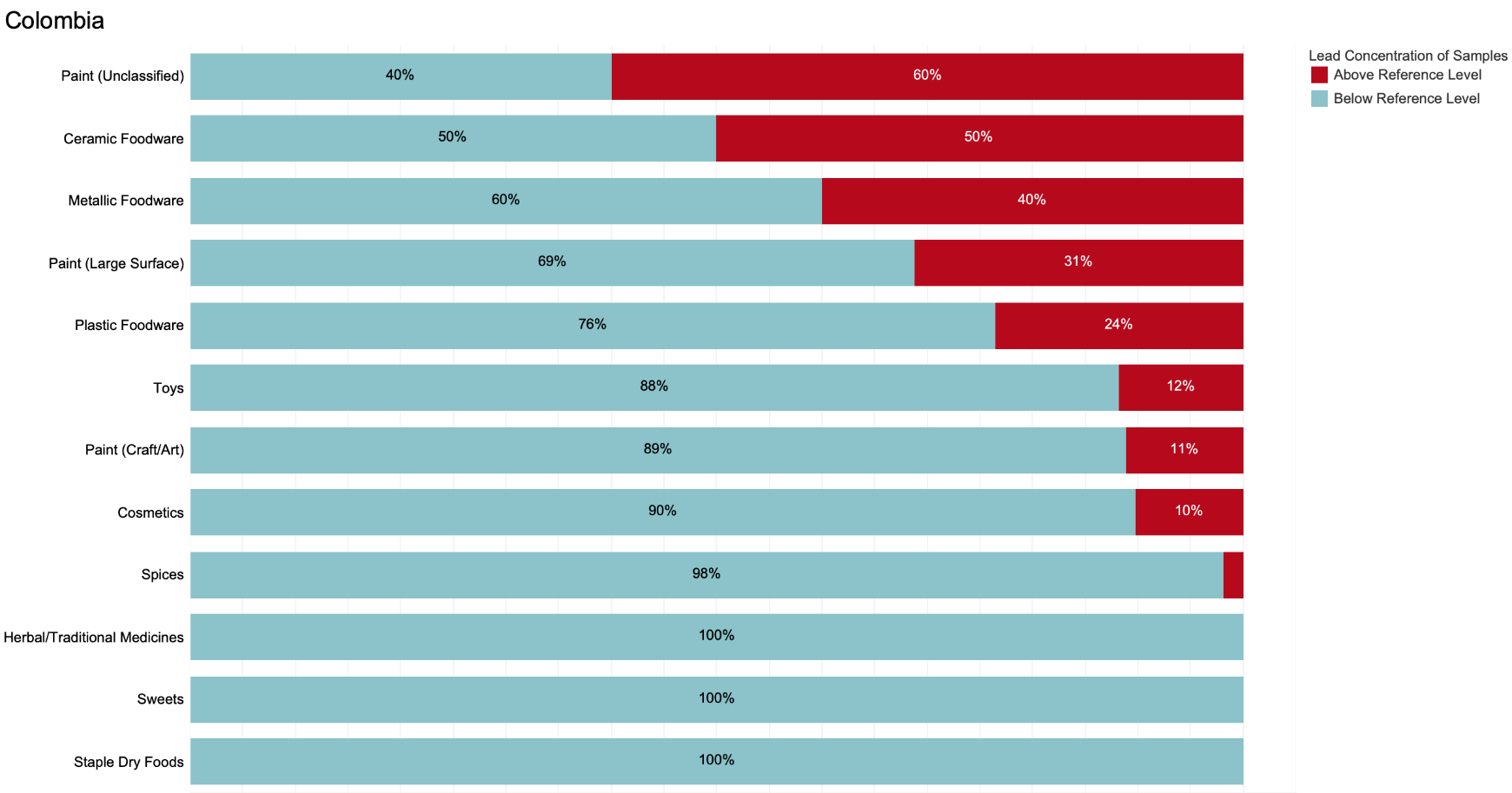


**Key:** Blue = percentage of samples below reference level. Red = percentage above reference level.

**Note**: Sample sizes below 5 are not displayed.

## Egypt

Pure Earth analyzed a total of 157 samples from Egypt, and of these, 15% exceeded the relevant reference levels. Like many countries, metal and ceramic foodware had a particularly high percentage of samples exceeding the reference levels, but cosmetics also showed unusually elevated lead levels among the 19 samples.

Summary of Results from Egypt in Order of % Exceeding Reference Levels

| **Item Category** | **# of Samples** | **Min Value (ppm)** | **Median (ppm)** | **Max Value (ppm)** | **% Above Reference** |
| --- | --- | --- | --- | --- | --- |
| Metallic foodware | 11 | ND | 180 | 1086 | 55 |
| Ceramic foodware | 10 | ND | 158 | 50600 | 50 |
| Cosmetics | 19 | ND | ND | 13700 | 42 |
| Plastic foodware | 15 | ND | ND | 1121 | 13 |
| Toys | 26 | ND | ND | 967 | 4 |
| Spices | 59 | ND | ND | 3 | 2 |
| Herbal/Trad Medicines | 10 | ND | ND | ND | 0 |
| Paints - large surfaces | 3 | ND | ND | ND | 0 |
| Staple dry food | 4 | ND | ND | ND | 0 |

ND = “non-detect” (lead concentration was below the XRF’s lower detection limit)

Percentage of Samples from Egypt Below and Above the Reference Level


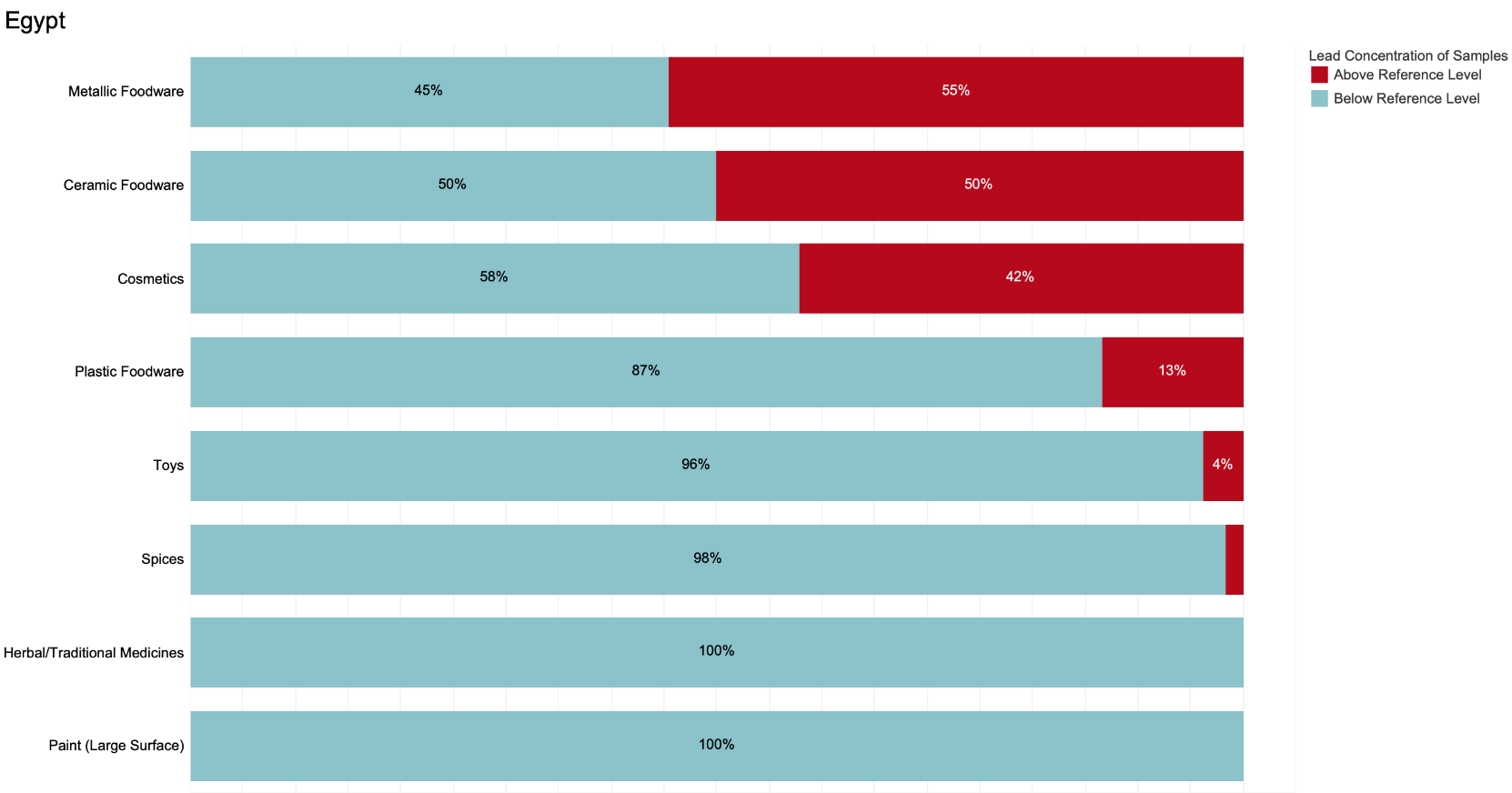


**Key:** Blue = percentage of samples below reference level. Red = percentage above reference level.

**Note**: Sample sizes below 5 are not displayed.

##

## Georgia

Pure Earth analyzed 186 samples from Georgia, and of these, 12% exceeded the relevant reference levels. Like many countries, foodware and paints had a particularly high percentage of samples exceeding the reference levels. The absence of any spice samples with elevated lead levels is particularly notable and is discussed in greater detail below.

Summary of Results from Georgia in Order of % Exceeding Reference Levels

| **Item Category** | **# of Samples** | **Min Value (ppm)** | **Median (ppm)** | **Max Value 9ppm)** | **% Above Reference** |
| --- | --- | --- | --- | --- | --- |
| Paint - unclassified | 2 | 685 | 881 | 1077 | 100 |
| Paints - large surfaces | 4 | ND | 376 | 22600 | 50 |
| Ceramic foodware | 27 | ND | 76 | 13200 | 48 |
| Metallic foodware | 19 | ND | ND | 119500 | 16 |
| Paint - craft/art | 14 | ND | ND | 4449 | 7 |
| Toys | 38 | ND | ND | 376 | 3 |
| Cosmetics | 27 | ND | ND | ND | 0 |
| Plastic foodware | 5 | ND | ND | ND | 0 |
| Spices | 45 | ND | ND | ND | 0 |
| Staple dry food | 5 | ND | ND | ND | 0 |

ND = “non-detect” (lead concentration was below the XRF’s lower detection limit)

Percentage of Samples from Georgia Below and Above the Reference Level


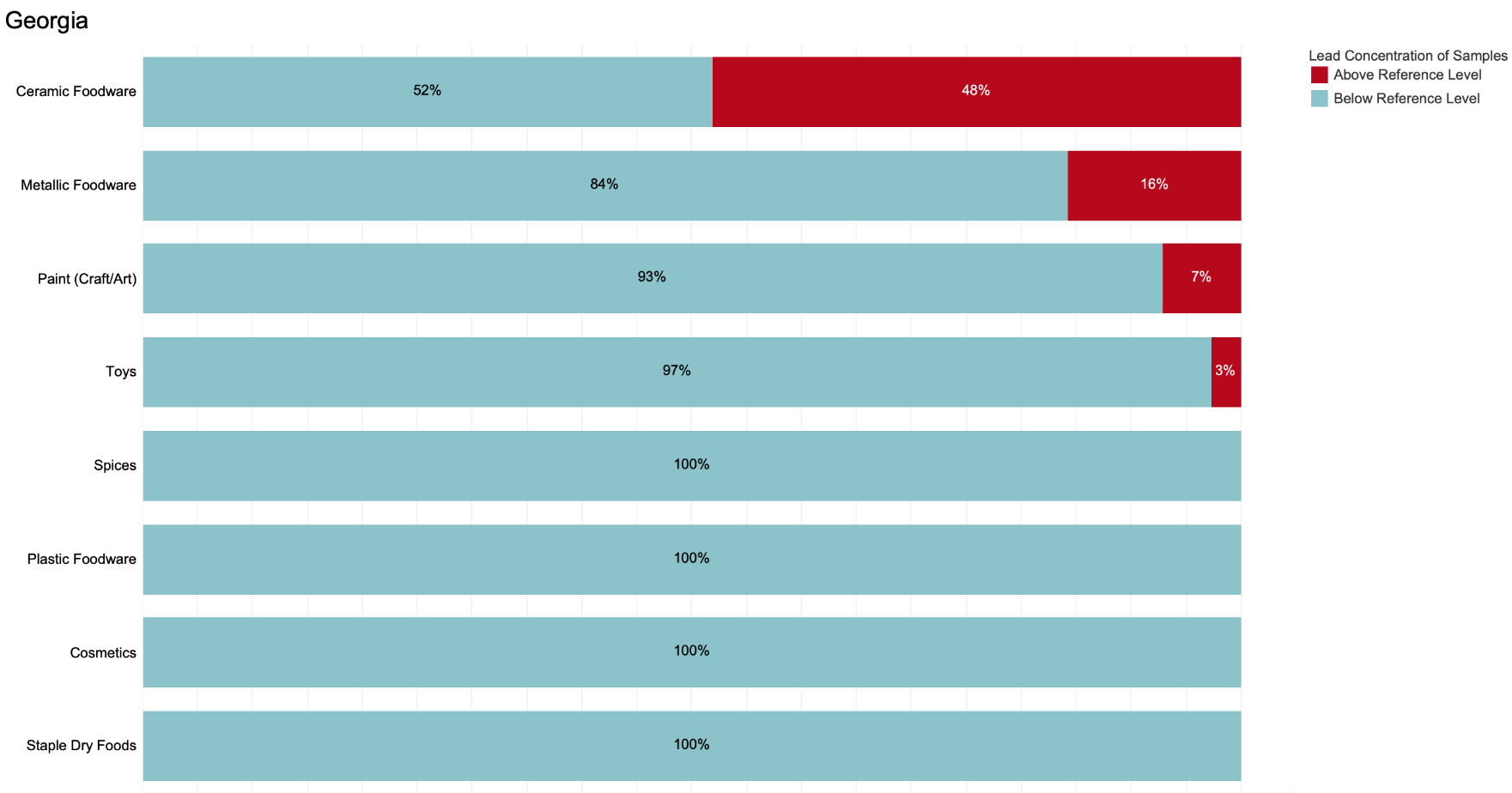


**Key:** Blue = percentage of samples below reference level. Red = percentage above reference level.

**Note**: Sample sizes below 5 are not displayed.

Of particular interest in the RMS results was the absence of any Georgian spice samples with detectable lead levels. Since 2018, Georgia has been the subject of much research and activity by the Government of Georgia, Pure Earth, UNICEF, and others aimed at assessing and reducing childhood lead poisoning following findings from a 2018 survey that suggested 41% of Georgian children had blood lead levels exceeding 5 μg/dL. Since then, particular emphasis has been placed on eliminating the practice of adulterating spices with lead-based pigments, particularly lead chromate, which had been used by some spice producers to enhance color. Under a separate program unrelated to the RMS, Pure Earth assessed lead levels in hundreds of spice sample between 2020 and 2023. The sampling results from those prior assessments show a considerable decline in lead levels since 2020. The RMS results are aligned with the trends seen in these other assessments and suggest that efforts to eliminate the practice of adulteration have been successful.

**Results from Prior Assessments of Lead in Georgian Spices by Pure Earth**

| **Region in Georgia** | **1^st^ round of testing (2020)** | | | **2^nd^ round of testing (2022)** | | |  |
| --- | --- | --- | --- | --- | --- | --- | --- |
|  | **Number of samples tested** | **Maximum lead concentration (ppm)** | **Average lead concentration (ppm)** | **Number of samples tested** | **Maximum lead concentration (ppm)** | **Average lead concentration (ppm)** |  |
|  |  |  |  |  |  |  |  |
|  |  |  |  |  |  |  |  |
| Adjara | 93 | 14800 | 359 | 93 | 46 | 4 |  |
| Tbilisi | 23 | 156 | 7 | 23 | 6 | 0.8 |  |
| Imereti | 38 | 47 | 2 | 31 | 11 | 1.3 |  |
| Shida Kartli | 21 | 2 | 0 | 21 | 3 | 0.5 |  |
| Kvemo Kartli | 12 | 5 | 1 | 12 | 7 | 1.5 |  |
| Guria | 10 | 98 | 10 | 10 | 12 | 2.8 |  |
| Samegrelo | 23 | 4 | 0.5 | 23 | 4 | 1.1 |  |
| Samtskhe | 13 | 4 | 0.4 | 13 | 10 | 2.7 |  |
| Kakheti | 19 | 3 | 0.5 | 19 | 4441* | 234* |  |
| Mtskheta-Mtianeti | 10 | 2 | 0.7 | 10 | 5 | 1.4 |  |

*We believe the results in red are influenced by a single spice sample from an old batch of spices that the producer manufactured years earlier, prior to interventions aimed at preventing adulteration.

##

## Ghana

Pure Earth analyzed a total of 193 samples from Ghana, and of these, 10% exceeded the relevant reference levels. As in many countries, metallic foodware had a particularly high percentage of samples exceeding the reference levels.

Summary of Results from Ghana in Order of % Exceeding Reference Levels

| **Item Category** | **# of Samples** | **Min Value (ppm)** | **Median (ppm)** | **Max Value (ppm)** | **% Above reference** |
| --- | --- | --- | --- | --- | --- |
| Metallic foodware | 22 | ND | 181 | 24100 | 55 |
| Ceramic foodware | 11 | 30 | 50 | 6570 | 18 |
| Toys | 22 | ND | ND | 1533 | 14 |
| Cosmetics | 28 | ND | ND | 220 | 7 |
| Paint - unclassified | 18 | ND | ND | 4 | 0 |
| Paint - craft/art | 3 | ND | ND | ND | 0 |
| Paints - large surfaces | 1 | ND | ND | ND | 0 |
| Plastic foodware | 12 | ND | ND | 32 | 0 |
| Spices | 47 | ND | ND | ND | 0 |
| Staple dry food | 29 | ND | ND | ND | 0 |

ND = “non-detect” (lead concentration was below the XRF’s lower detection limit)

Percentage of Samples from Ghana Below and Above the Reference Level


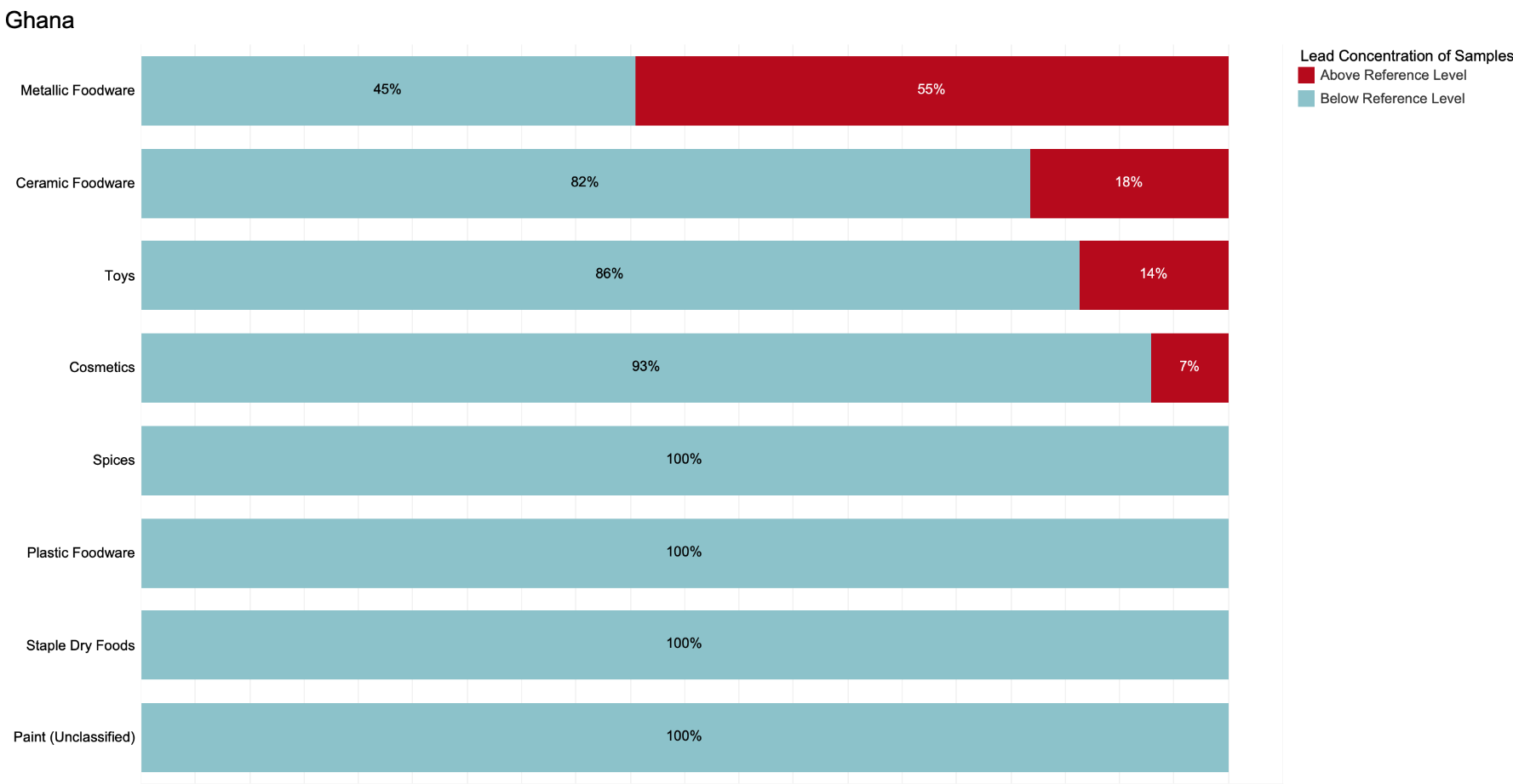


**Key:** Blue = percentage of samples below reference level. Red = percentage above reference level.

**Note**: Sample sizes below 5 are not displayed.

## India - Maharashtra State

Pure Earth analyzed a total of 257 samples from Maharashtra State, India, and of these, 17% exceeded the relevant reference levels. Foodware, paints, and toys showed particularly high lead levels compared with other sample categories. Interestingly, contaminated spices, which other research projects have found to be prevalent in North and Northeast India, did not show up in the spice samples from Maharashtra, which spans from Central India to the West Coast.

Summary of Results from Maharashtra in Order of % Exceeding Reference Levels

| **Item Category** | **# of Samples** | **Min Value (ppm)** | **Median (ppm)** | **Max Value (ppm)** | **% Above reference** |
| --- | --- | --- | --- | --- | --- |
| Ceramic foodware | 17 | ND | 1910 | 80000 | 71 |
| Metallic foodware | 19 | ND | 720 | 6590 | 63 |
| Toys | 34 | ND | ND | 97300 | 21 |
| Plastic foodware | 16 | ND | ND | 437 | 19 |
| Paints - large surfaces | 27 | ND | ND | 164000 | 19 |
| Paint - craft/art | 12 | ND | ND | 1616 | 17 |
| Cosmetics | 69 | ND | ND | 60 | 3 |
| Herbal/Trad Medicines | 3 | ND | ND | ND | 0 |
| Spices | 50 | ND | ND | ND | 0 |
| Staple dry food | 10 | ND | ND | ND | 0 |

ND = “non-detect” (lead concentration was below the XRF’s lower detection limit)

Percentage of Samples from Maharashtra Below and Above the Reference Level


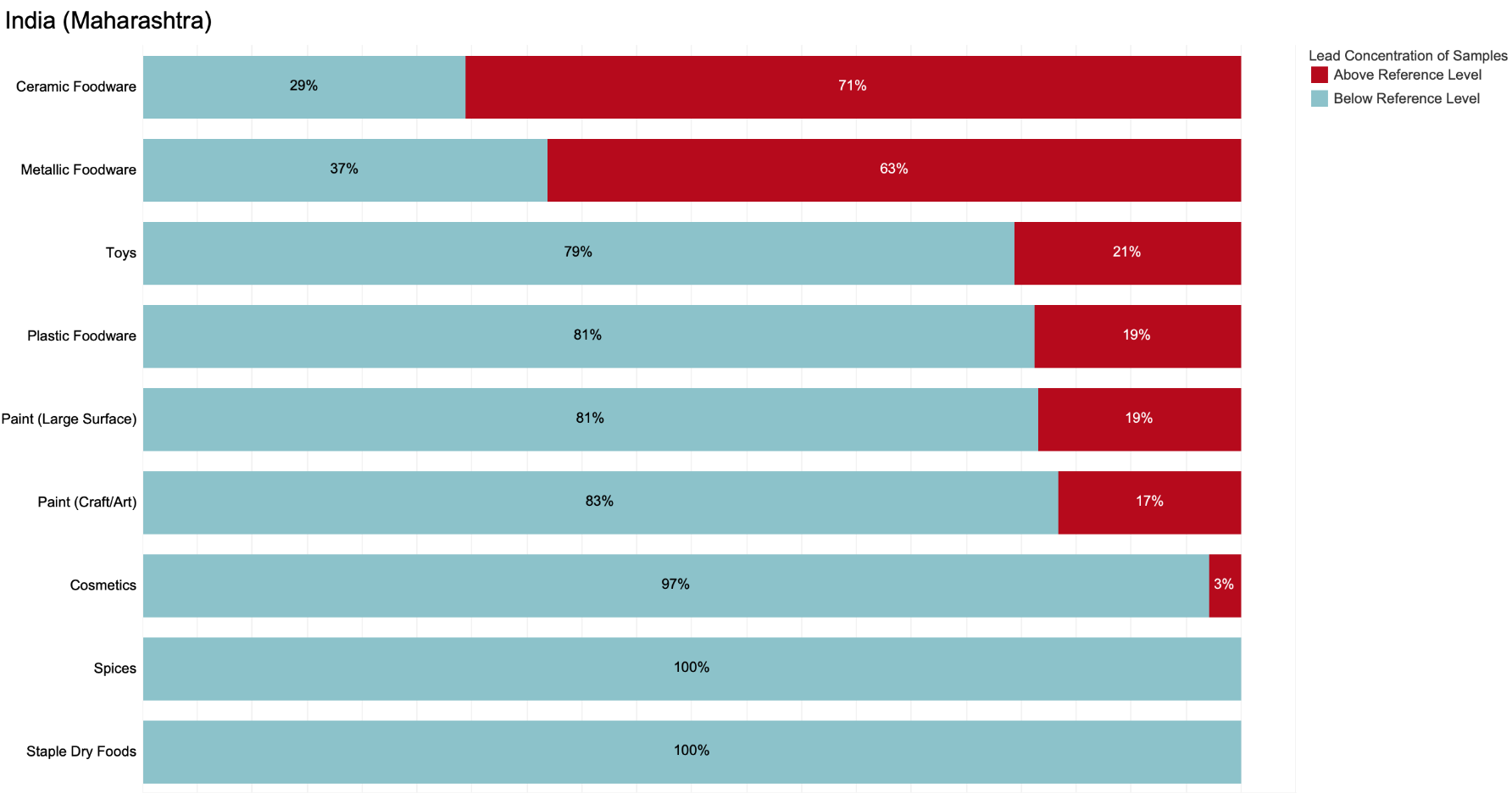


**Key:** Blue = percentage of samples below reference level. Red = percentage above reference level.

**Note**: Sample sizes below 5 are not displayed.

## India - Tamil Nadu State

Pure Earth analyzed 188 samples from Tamil Nadu State, India, and of these, 30% exceeded the relevant reference levels. As with many locations, samples of foodware and paints often exceeded reference levels, with samples of toys and cosmetics also showing elevated levels among some samples. As with Maharashtra State, spice samples from Tamil Nadu, which sits at the Southeastern tip of India, did not show the type of elevated lead levels that have been found in India’s Northern and Northeastern States.

Summary of Results from Tamil Nadu in Order of % Exceeding Reference Levels

| **Item Category** | **# of Samples** | **Min Value (ppm)** | **Median (ppm)** | **Max Value (ppm)** | **% Above Reference** |
| --- | --- | --- | --- | --- | --- |
| Paint - unclassified | 24 | ND | 2915 | 40700 | 79 |
| Metallic foodware | 27 | ND | 870 | 13900 | 70 |
| Paints - large surfaces | 7 | ND | 2356 | 13400 | 57 |
| Ceramic foodware | 8 | ND | 75 | 5230 | 50 |
| Toys | 30 | ND | ND | 3250 | 23 |
| Plastic foodware | 7 | ND | ND | 872 | 14 |
| Cosmetics | 32 | ND | ND | 231 | 9 |
| Spices | 44 | ND | ND | ND | 0 |
| Staple dry food | 9 | ND | ND | ND | 0 |

ND = “non-detect” (lead concentration was below the XRF’s lower detection limit)

Percentage of Samples from Tamil Nadu Below and Above the Reference Level


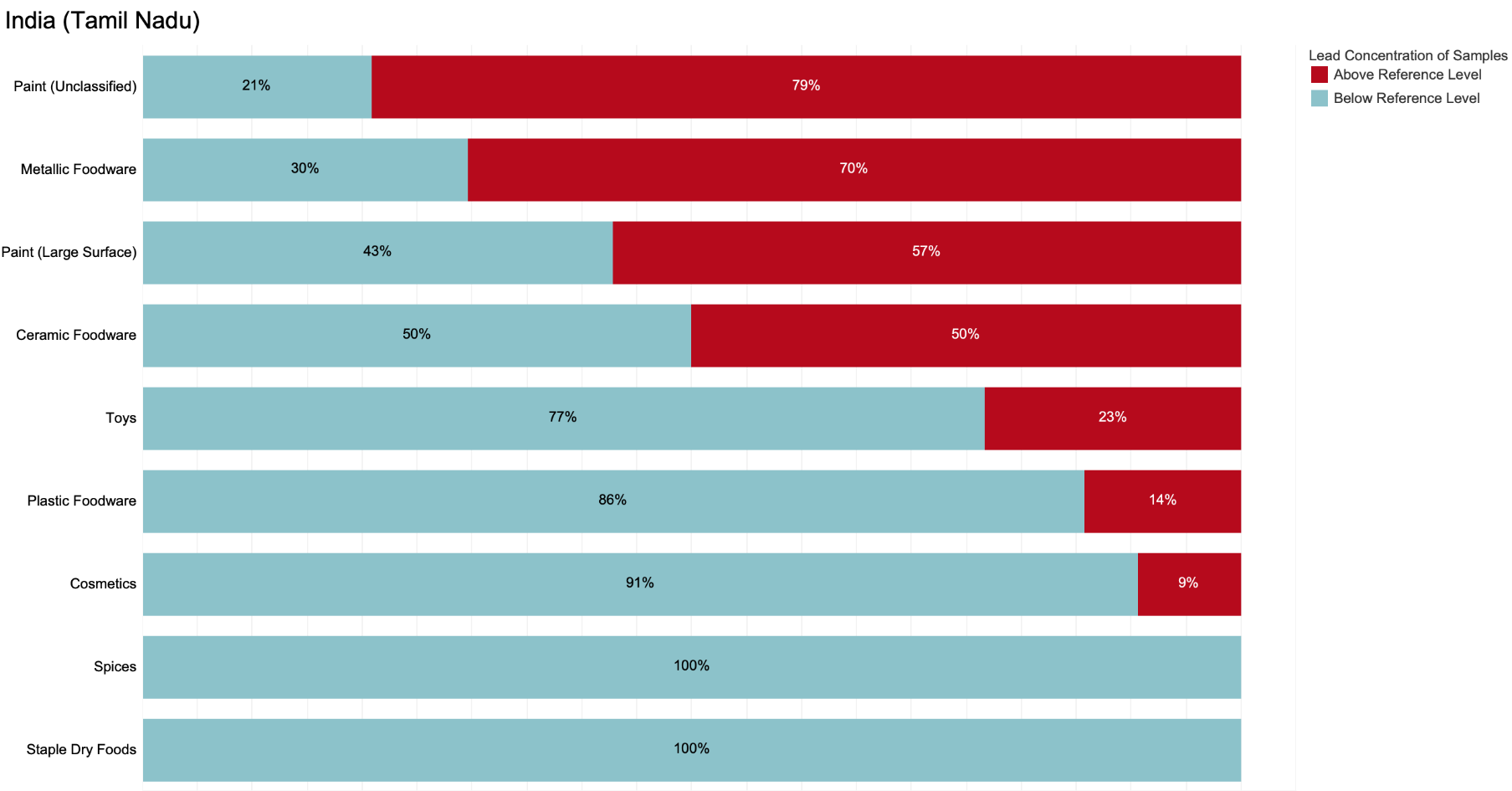


**Key:** Blue = percentage of samples below reference level. Red = percentage above reference level.

**Note**: Sample sizes below 5 are not displayed.

## India - Uttar Pradesh State

Pure Earth analyzed a total of 202 samples from Uttar Pradesh State, India, and of these, 19% exceeded the relevant reference levels. Samples of metallic foodware and paints often exceeded the relevant reference levels. Unlike Maharashtra and Tamil Nadu States, spice samples from Uttar Pradesh did exhibit elevated lead levels, which is consistent with findings from other recent lead exposure source assessment programs that have identified lead-contaminated spices in India’s north and northeast.

Summary of Results from Uttar Pradesh in Order of % Exceeding Reference Levels

| **Item Category** | **# of Samples** | **Min Value (ppm)** | **Median** | **Max Value (ppm)** | **% Above Reference** |
| --- | --- | --- | --- | --- | --- |
| Metallic foodware | 17 | ND | 850 | 74600 | 65 |
| Paints - large surfaces | 31 | ND | ND | 33200 | 42 |
| Toys | 38 | ND | ND | 4680 | 24 |
| Spices | 41 | ND | ND | 622 | 12 |
| Cosmetics | 44 | ND | ND | ND | 0 |
| Ceramic foodware | 6 | ND | 20 | 80 | 0 |
| Herbal/Trad Medicines | 4 | ND | ND | ND | 0 |
| Plastic foodware | 18 | ND | ND | 11 | 0 |
| Staple dry food | 3 | ND | ND | ND | 0 |

ND = “non-detect” (lead concentration was below the XRF’s lower detection limit)

Percentage of Samples from Uttar Pradesh Below and Above the Reference Level


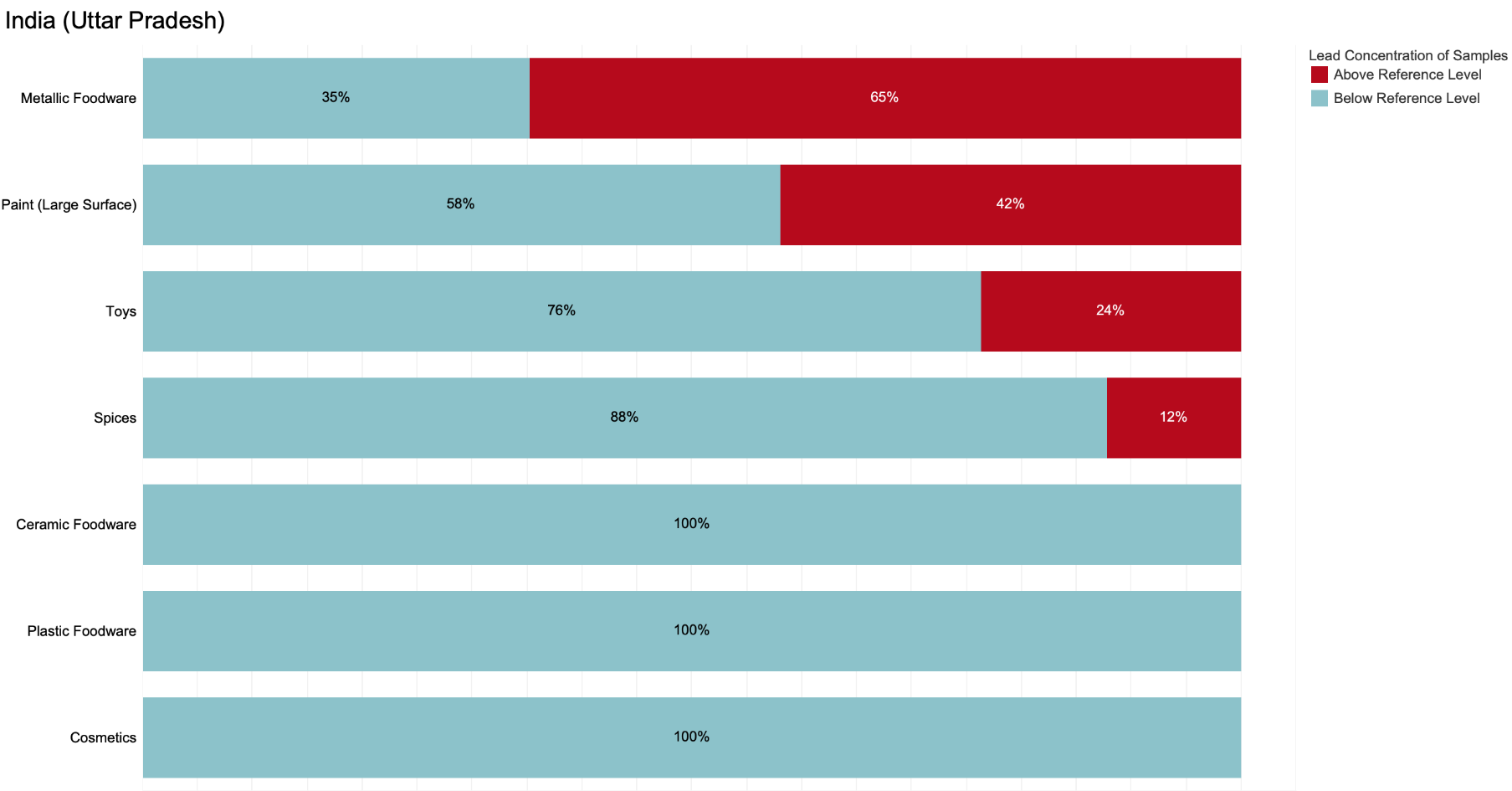


**Key:** Blue = percentage of samples below reference level. Red = percentage above reference level.

**Note**: Sample sizes below 5 are not displayed.

## Indonesia

Pure Earth analyzed a total of 176 samples from Indonesia, and of these, 40% exceeded the relevant reference levels. Metallic foodware, paints, and cosmetics emerged as the products with the highest percentage of samples exceeding the relevant reference levels. Of particular note is that among the 31 paint samples, 97% exceeded the reference level of 90 ppm.

Summary of Results from Indonesia in Order of % Exceeding Reference Levels

| **Item Category** | **# of Samples** | **Min Value (ppm)** | **Median (ppm)** | **Max Value (ppm)** | **% Above Reference** |
| --- | --- | --- | --- | --- | --- |
| Paints - large surfaces | 31 | 1 | 3142 | 51400 | 97 |
| Metallic foodware | 45 | ND | 410 | 18100 | 60 |
| Cosmetics | 36 | ND | ND | 12 | 33 |
| Toys | 21 | ND | ND | 314 | 10 |
| Spices | 34 | ND | ND | ND | 0 |
| Staple dry food | 9 | ND | ND | ND | 0 |

ND = “non-detect” (lead concentration was below the XRF’s lower detection limit)

Percentage of Samples from Indonesia Below and Above the Reference Level


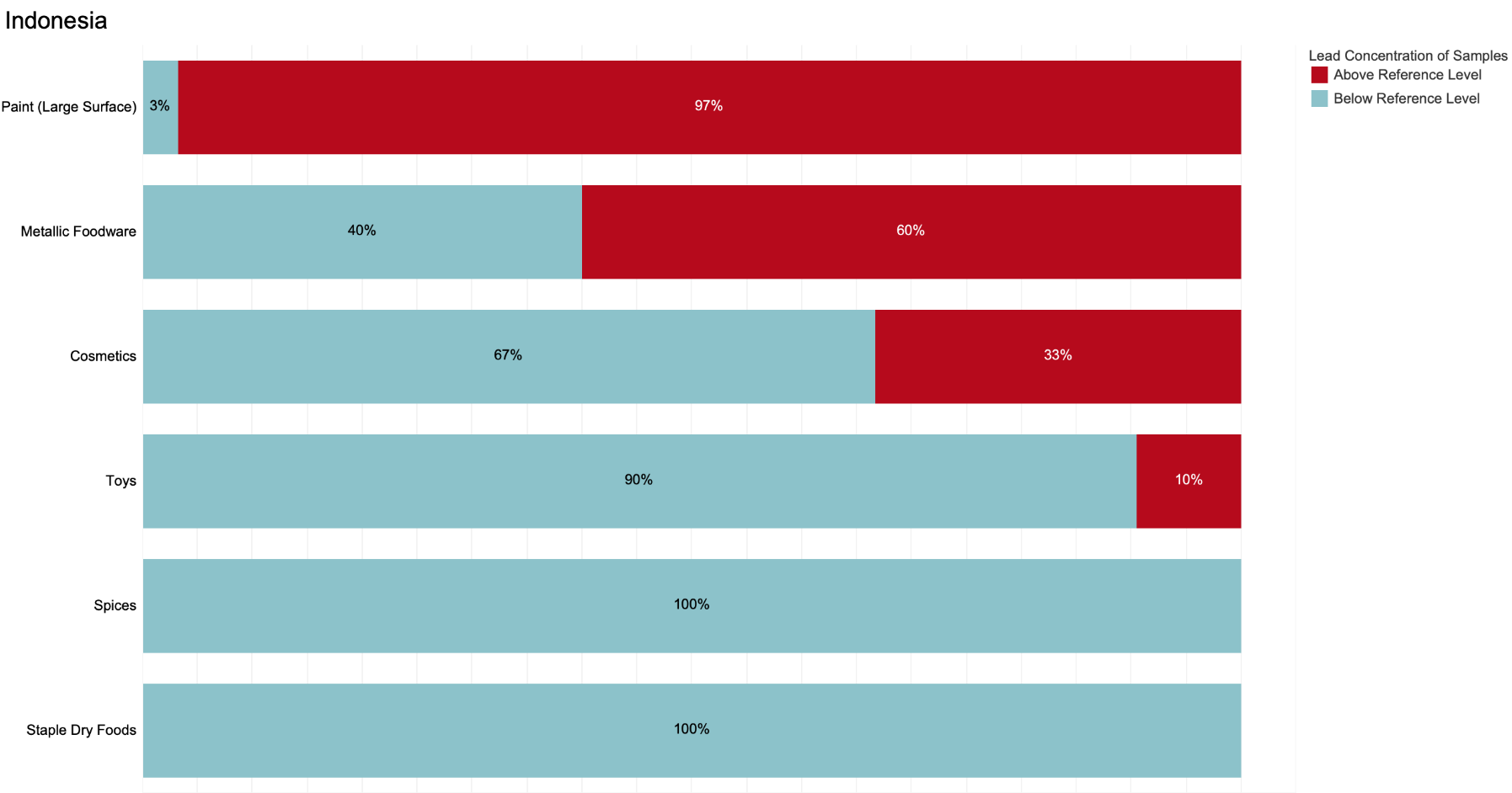


**Key:** Blue = percentage of samples below reference level. Red = percentage above reference level.

**Note**: Sample sizes below 5 are not displayed.

## Kazakhstan

This report includes lead concentrations found in 18 samples from Kazakhstan, and of these, 6% exceeded the relevant reference levels. Readers should note that Pure Earth collected and conducted field XRF analyses on 163 samples from Kazakhstan, however, the field XRF results were expunged after confirmatory testing of samples sent to New York suggested that the field XRF used in Kazakhstan was not providing sufficiently accurate readings. This issue is discussed more fully in the Quality Control section. The results below are from a subset of samples sent to New York for confirmatory analysis.

Summary of Results from Kazakhstan in Order of % Exceeding Reference Levels

| **Item Category** | **# of Samples** | **Min Value (ppm)** | **Median (ppm)** | **Max Value (ppm)** | **% Above Reference** |
| --- | --- | --- | --- | --- | --- |
| Toys | 3 | ND | ND | 1138 | 33 |
| Cosmetics | 4 | ND | ND | ND | 0 |
| Spices | 8 | ND | ND | ND | 0 |
| Staple food | 3 | ND | ND | ND | 0 |

ND = “non-detect” (lead concentration was below the XRF’s lower detection limit)

Percentage of Samples from Kazakhstan Below and Above the Reference Level


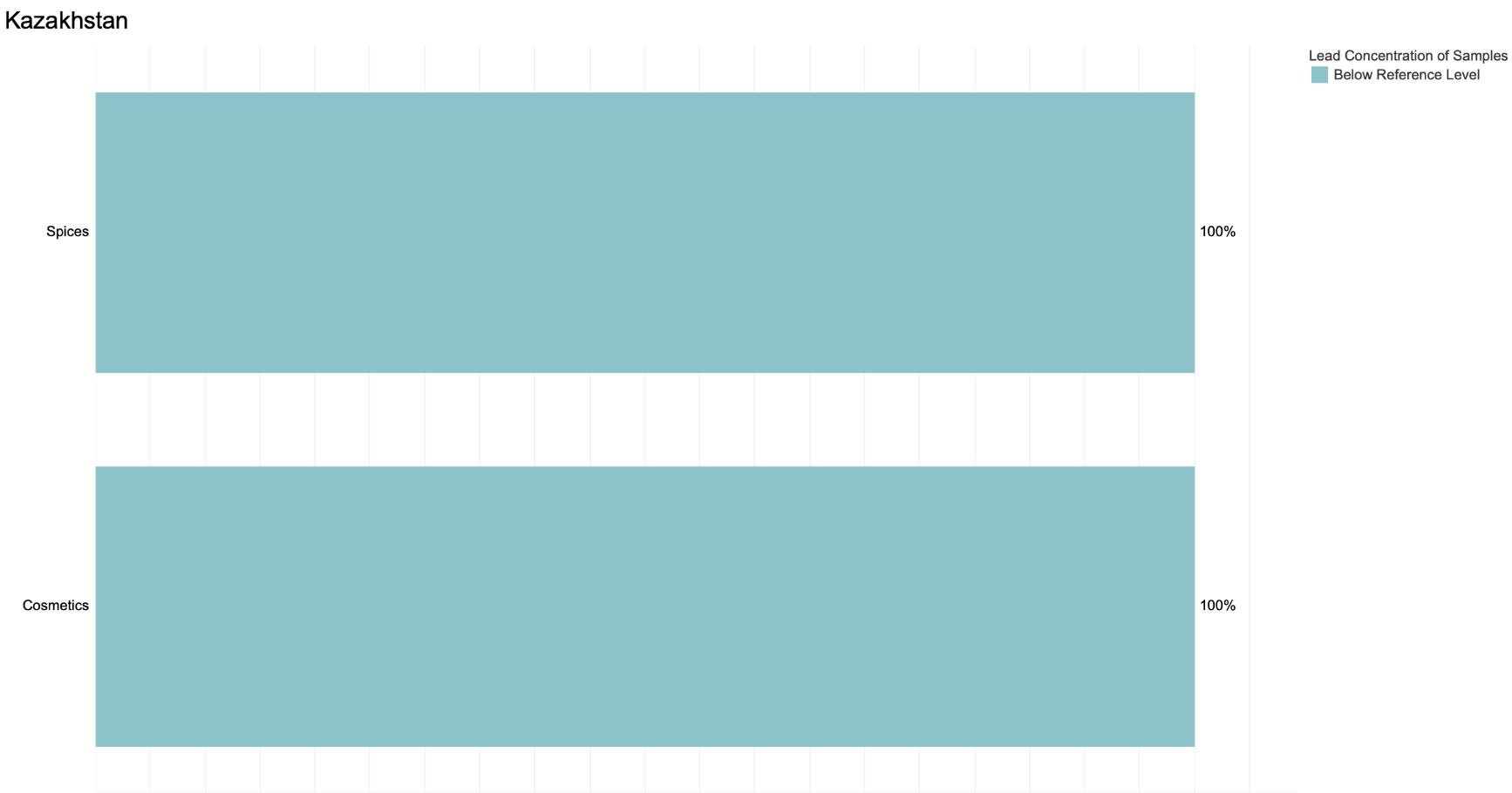


**Key:** Blue = percentage of samples below reference level. Red = percentage above reference level.

**Note**: Sample sizes below 5 are not displayed.

## Kenya

Pure Earth analyzed a total of 192 samples from Kenya, and of these, 19% exceeded the relevant reference levels. Metallic foodware, ceramic foodware, and paint samples most commonly exceeded reference levels.

Summary of Results from Kenya in Order of % Exceeding Reference Levels

| **Item Category** | **# of Samples** | **Min Value (ppm)** | **Median (ppm)** | **Max Value (ppm)** | **% Above Reference** |
| --- | --- | --- | --- | --- | --- |
| Ceramic foodware | 21 | ND | 4210 | 91000 | 62 |
| Metallic foodware | 15 | 21 | 130 | 3600 | 53 |
| Paints - large surfaces | 25 | ND | 7 | 7788 | 36 |
| Plastic foodware | 12 | ND | ND | 2395 | 25 |
| Cosmetics | 32 | ND | ND | 6 | 6 |
| Toys | 38 | ND | ND | 139 | 3 |
| Spices | 41 | ND | ND | ND | 0 |
| Staple dry food | 8 | ND | ND | ND | 0 |

ND = “non-detect” (lead concentration was below the XRF’s lower detection limit)

Percentage of Samples from Kenya Below and Above the Reference Level


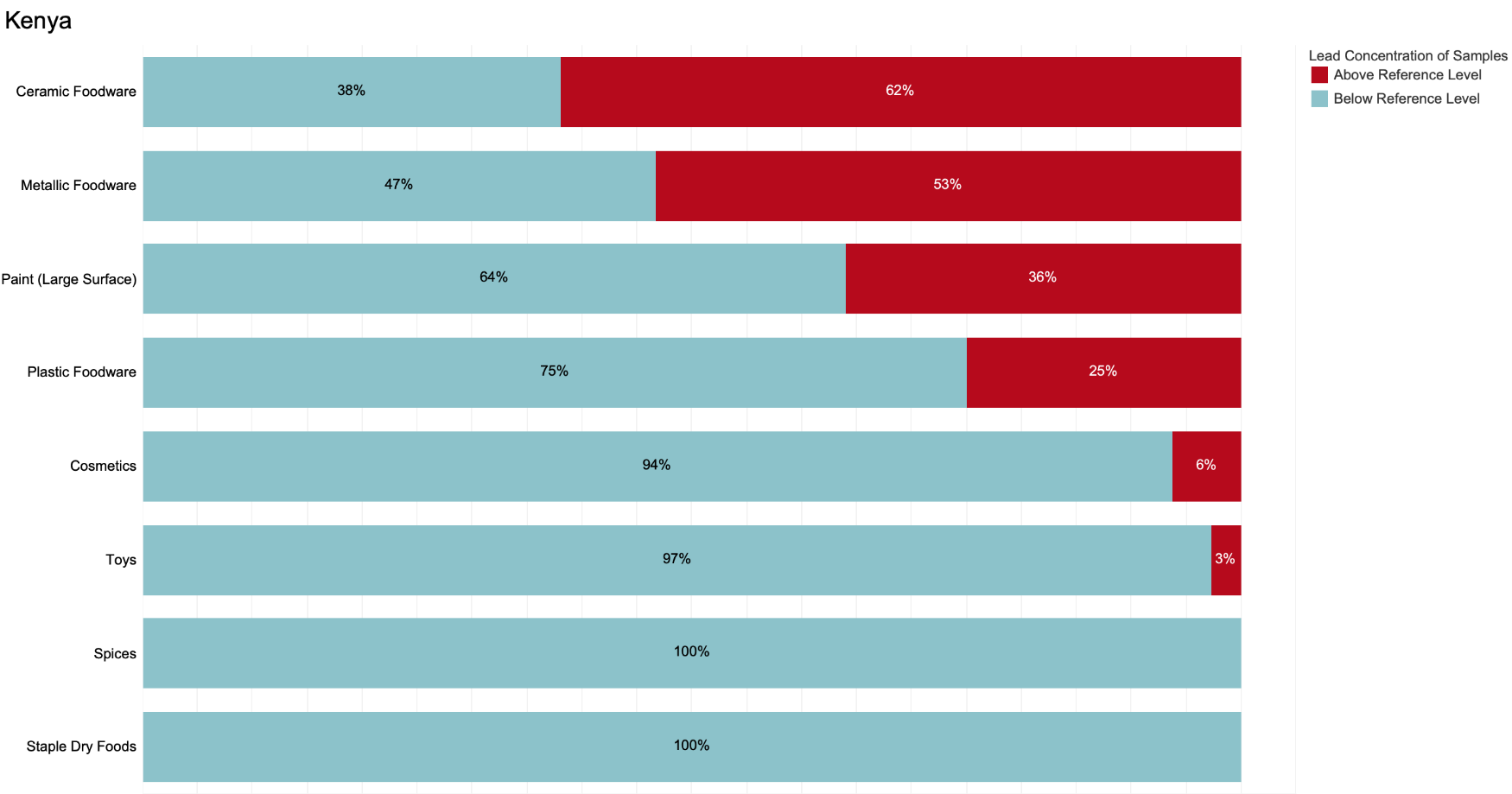


**Key:** Blue = percentage of samples below reference level. Red = percentage above reference level.

**Note**: Sample sizes below 5 are not displayed.

## Kyrgyzstan

Pure Earth analyzed a total of 208 samples from Kyrgyzstan, and of these, 14% exceeded the relevant reference levels. Foodware, paints, and cosmetics stand out as possible issues of concern.

Summary of Results from Kyrgyzstan in Order of % Exceeding Reference Levels

| **Item Category** | **# of Samples** | **Min Value (ppm)** | **Median (ppm)** | **Max Value (ppm)** | **% Above Reference** |
| --- | --- | --- | --- | --- | --- |
| Ceramic foodware | 16 | ND | 73 | 240500 | 44 |
| Paints - large surfaces | 33 | ND | 10 | 890 | 33 |
| Metallic foodware | 16 | ND | 8 | 494 | 19 |
| Cosmetics | 32 | ND | ND | 7 | 16 |
| Plastic foodware | 16 | ND | ND | 368 | 13 |
| Toys | 33 | ND | ND | 314 | 6 |
| Spices | 48 | ND | ND | ND | 0 |
| Staple dry food | 14 | ND | ND | ND | 0 |

ND = “non-detect” (lead concentration was below the XRF’s lower detection limit)

Percentage of Samples from Kyrgyzstan Below and Above the Reference Level


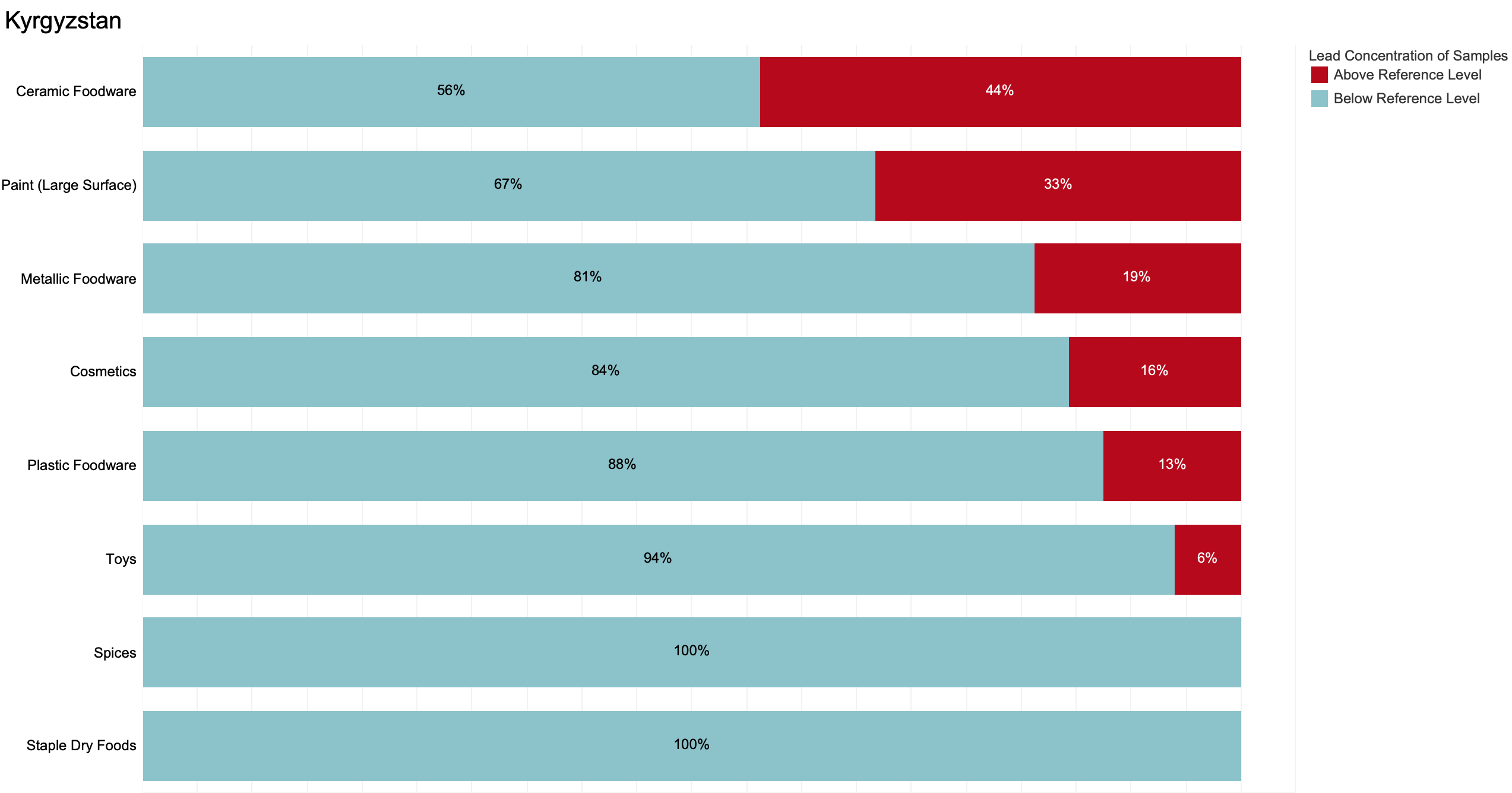


**Key:** Blue = percentage of samples below reference level. Red = percentage above reference level.

**Note**: Sample sizes below 5 are not displayed.

## Mexico

Pure Earth analyzed a total of 206 samples from Mexico, and of these, 17% exceeded the relevant reference levels. Ceramic foodware, metallic foodware, paints, and toys emerged as the products with the highest percentage of samples exceeding the relevant reference levels. Contaminated ceramic foodware is a well-known challenge in Mexico, and the high prevalence of samples exceeding the reference level is in line with findings from other assessments. Of particular note is the fact that out of the 15 samples of paint intended for large surfaces, 93% exceeded the reference level of 90 ppm.

Summary of Results from Mexico in Order of % Exceeding Reference Levels

| **Item Category** | **# of Samples** | **Min Value (ppm)** | **Median (ppm)** | **Max Value (ppm)** | **% Above Reference** |
| --- | --- | --- | --- | --- | --- |
| Paints - large surfaces | 15 | 3 | 53700 | 807309 | 93 |
| Ceramic foodware | 6 | ND | 19215 | 65700 | 67 |
| Metallic foodware | 16 | ND | 30 | 900 | 25 |
| Paint - unclassified | 8 | ND | 2 | 79000 | 25 |
| Toys | 27 | ND | ND | 1070 | 22 |
| Plastic foodware | 13 | ND | ND | 853 | 8 |
| Cosmetics | 29 | ND | ND | 50 | 7 |
| Sweets | 48 | ND | ND | 5 | 4 |
| Spices | 29 | ND | ND | 10 | 3 |
| Herbal/Trad Medicines | 4 | ND | ND | ND | 0 |
| Staple dry food | 11 | ND | ND | ND | 0 |

ND = “non-detect” (lead concentration was below the XRF’s lower detection limit)

Percentage of Samples from Mexico Below and Above the Reference Level


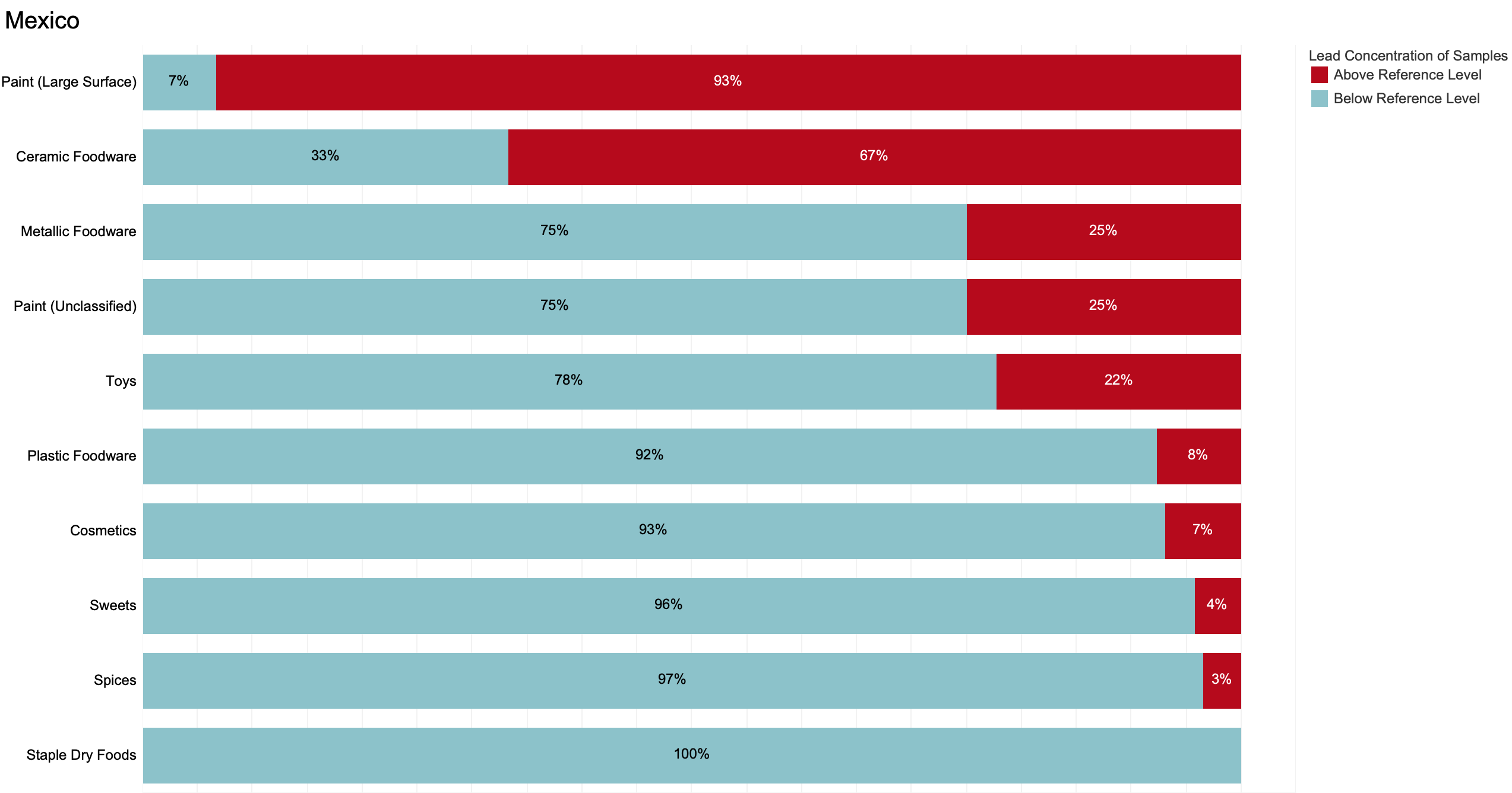


**Key:** Blue = percentage of samples below reference level. Red = percentage above reference level.

**Note**: Sample sizes below 5 are not displayed.

## Nepal

Pure Earth analyzed a total of 168 samples from Nepal, and of these, 8% exceeded the relevant reference levels. In Nepal, lead was only detected in ceramic foodware (18% of samples), metallic foodware (100%), and plastic foodware (6%).

Summary of Results from Nepal in Order of % Exceeding Reference Levels

| **Item Category** | **# of Samples** | **Min Value (ppm)** | **Median (ppm)** | **Max Value (ppm)** | **% Above Reference** |
| --- | --- | --- | --- | --- | --- |
| Metallic foodware | 11 | 170 | 750 | 3960 | 100 |
| Ceramic foodware | 11 | ND | ND | 9220 | 18 |
| Plastic foodware | 17 | ND | ND | 3448 | 6 |
| Cosmetics | 21 | ND | ND | ND | 0 |
| Herbal/Trad Medicines | 9 | ND | ND | ND | 0 |
| Paints - large surfaces | 20 | ND | ND | ND | 0 |
| Spices | 53 | ND | ND | ND | 0 |
| Staple dry food | 16 | ND | ND | ND | 0 |
| Toys | 10 | ND | ND | ND | 0 |

ND = “non-detect” (lead concentration was below the XRF’s lower detection limit)

Percentage of Samples from Nepal Below and Above the Reference Level


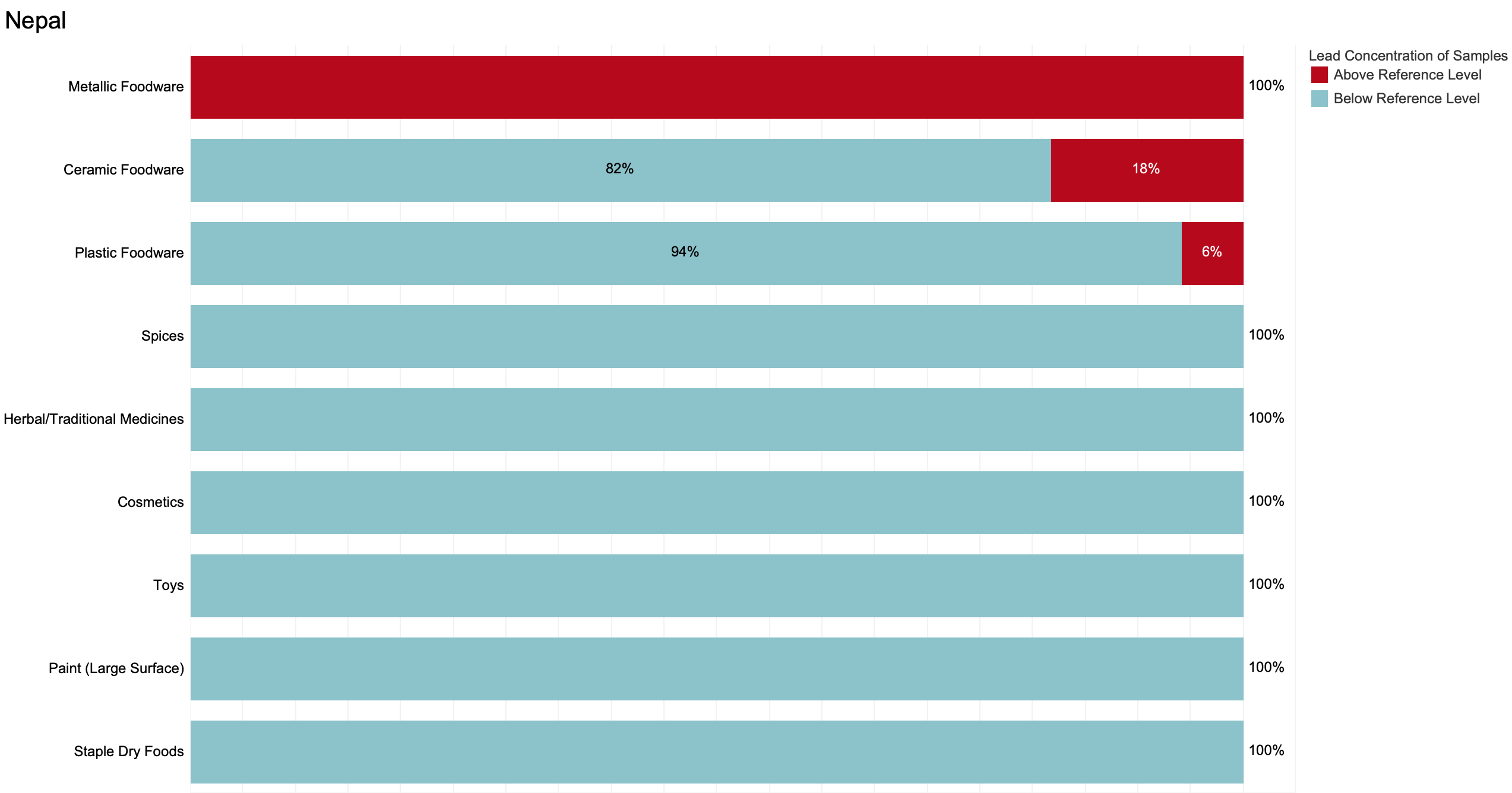


**Key:** Blue = percentage of samples below reference level. Red = percentage above reference level.

**Note**: Sample sizes below 5 are not displayed.

## Nigeria

Pure Earth analyzed a total of 353 samples from Nigeria, and of these, 19% exceeded the relevant reference levels. As with many other countries, metallic foodware, ceramic foodware, and paints emerged as the products with the highest percentage of samples exceeding the relevant reference levels. Samples of cosmetics and toys also showed elevated lead levels.

Summary of Results from Nigeria in Order of % Exceeding Reference Levels

| **Item Category** | **# of Samples** | **Min Value (ppm)** | **Median (ppm)** | **Max Value (ppm)** | **% Above Reference** |
| --- | --- | --- | --- | --- | --- |
| Paints - large surfaces | 29 | ND | 494 | 20700 | 76 |
| Metallic foodware | 35 | ND | 410 | 1000 | 66 |
| Ceramic foodware | 21 | 20 | 40 | 46000 | 29 |
| Cosmetics | 50 | ND | ND | 1150 | 18 |
| Toys | 44 | ND | ND | 2292 | 16 |
| Plastic foodware | 24 | ND | ND | 280 | 4 |
| Spices | 67 | ND | ND | ND | 0 |
| Staple dry food | 83 | ND | ND | ND | 0 |

ND = “non-detect” (lead concentration was below the XRF’s lower detection limit)

Percentage of Samples from Nigeria Below and Above the Reference Level


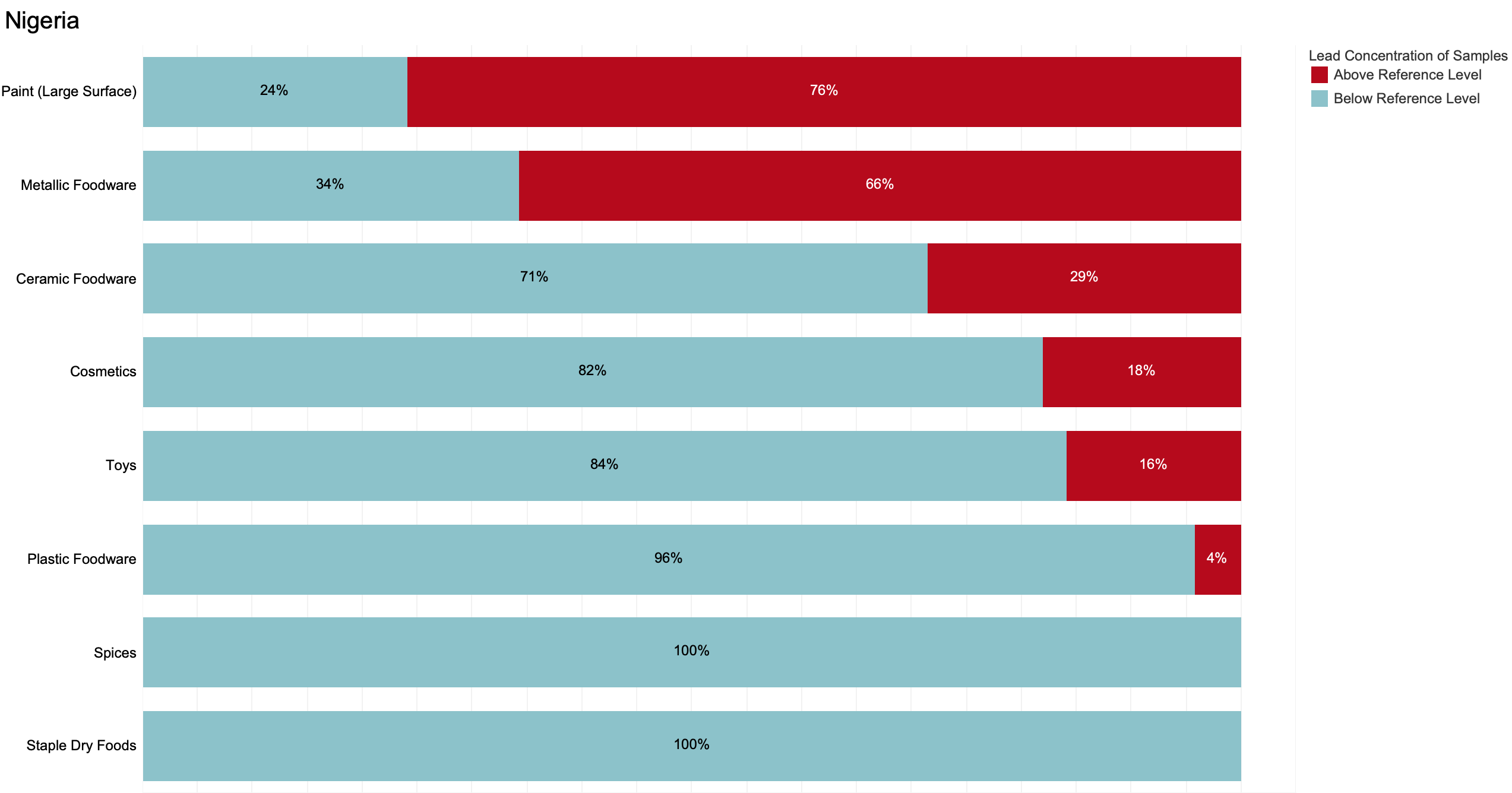


**Key:** Blue = percentage of samples below reference level. Red = percentage above reference level.

**Note**: Sample sizes below 5 are not displayed.

## Pakistan

Pure Earth analyzed 203 samples from Pakistan, and of these, 24% exceeded the relevant reference levels. As with many other countries, a high percentage of metallic foodware exceeded the relevant reference levels, with >20% of paints, cosmetics, and ceramics also showing elevated levels. Pakistan produces certain types of eyeliners that have been found to contain high concentrations of lead.

Note that there is some uncertainty with the levels of lead in spices from Pakistan as laboratory testing indicated lower levels than the XRF. Unlike field XRF results from Kazakhstan and Tajikistan, it was not clear to the Quality Control Team that the field XRF results for spice were inaccurate, or if other factors had contributed to discrepancies between field and lab results. Ultimately, field results were retained with a note regarding the uncertainty.

Summary of Results from Pakistan in Order of % Exceeding Reference Levels

| **Item Category** | **# of Samples** | **Min Value (ppm)** | **Median (ppm)** | **Max Value (ppm)** | **% Above Reference** |
| --- | --- | --- | --- | --- | --- |
| Metallic foodware | 28 | ND | 3238 | 7858 | 75 |
| Paints - large surfaces | 20 | ND | ND | 7370 | 35 |
| Cosmetics | 33 | ND | ND | 1000000 | 30 |
| Ceramic foodware | 5 | ND | 69 | 103 | 20 |
| Toys | 30 | ND | ND | 1481 | 13 |
| Spices | 46 | ND | ND | 160 | 9 |
| Plastic foodware | 12 | ND | ND | 2419 | 8 |
| Staple dry food | 9 | ND | ND | ND | 0 |
| Sweets | 20 | ND | ND | ND | 0 |

ND = “non-detect” (lead concentration was below the XRF’s lower detection limit)

Percentage of Samples from Pakistan Below and Above the Reference Level


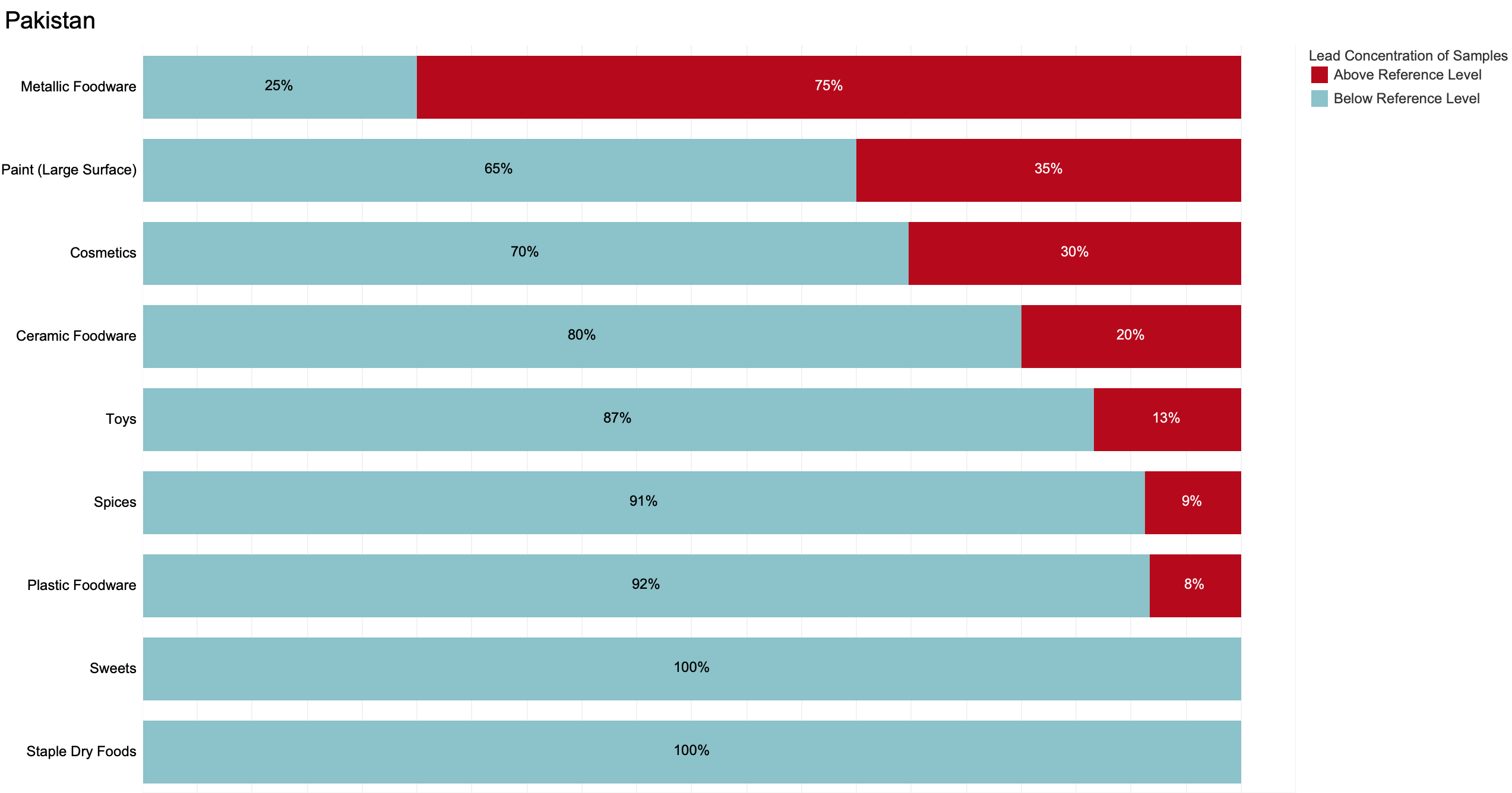


**Key:** Blue = percentage of samples below reference level. Red = percentage above reference level.

**Note**: Sample sizes below 5 are not displayed.

##

## Peru

Pure Earth analyzed a total of 228 samples from Peru, and of these, 15% exceeded the relevant reference levels. As with many other countries, foodware and paint most commonly exceeded the relevant reference levels.

Summary of Results from Peru in Order of % Exceeding Reference Levels

| **Item Category** | **# of Samples** | **Min Value (ppm)** | **Median (ppm)** | **Max Value (ppm)** | **% Above Reference** |
| --- | --- | --- | --- | --- | --- |
| Metallic foodware | 26 | ND | 217 | 90400 | 69 |
| Ceramic foodware | 12 | ND | 65 | 18600 | 42 |
| Paint - unclassified | 3 | ND | ND | 846 | 33 |
| Plastic foodware | 18 | ND | ND | 1643 | 17 |
| Paints - large surfaces | 10 | ND | ND | 2822 | 10 |
| Cosmetics | 44 | ND | ND | 128400 | 9 |
| Spices | 43 | ND | ND | 7 | 2 |
| Toys | 43 | ND | ND | 442 | 2 |
| Herbal/Trad Medicines | 2 | ND | ND | ND | 0 |
| Paint - craft/art | 17 | ND | ND | 32 | 0 |
| Staple dry food | 10 | ND | ND | ND | 0 |

ND = “non-detect” (lead concentration was below the XRF’s lower detection limit)

Percentage of Samples from Peru Below and Above the Reference Level


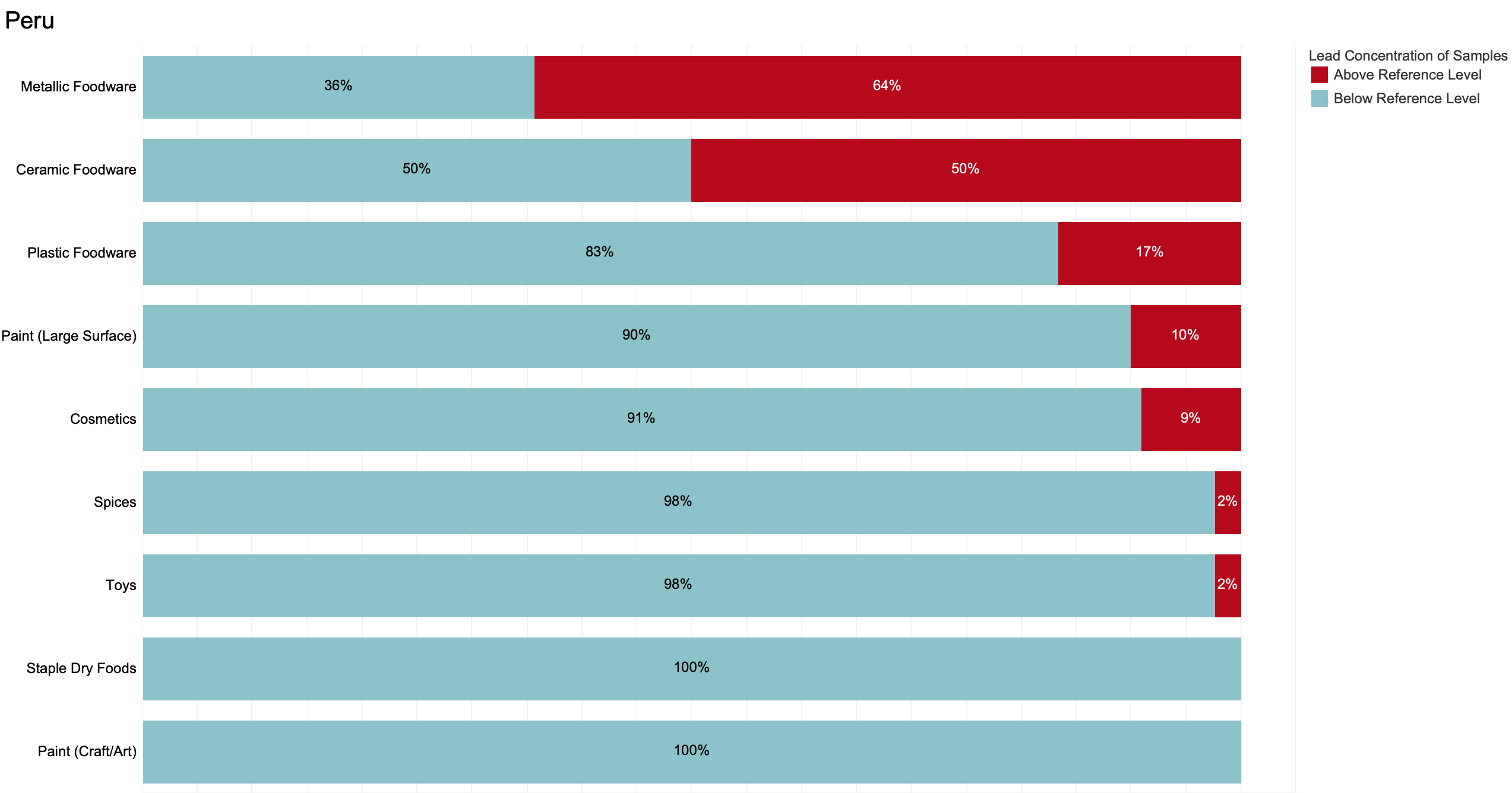


**Key:** Blue = percentage of samples below reference level. Red = percentage above reference level.

**Note**: Sample sizes below 5 are not displayed.

## The Philippines

Pure Earth analyzed a total of 265 samples from the Philippines, and of these, 8% exceeded the relevant reference levels. With one of the largest national datasets, the Philippines showed comparably low percentages of samples exceeding reference levels.

Summary of Results from the Philippines in Order of % Exceeding Reference Levels

| **Item Category** | **# of Samples** | **Min Value (ppm)** | **Median (ppm)** | **Max Value (ppm)** | **% Above Reference** |
| --- | --- | --- | --- | --- | --- |
| Paint - unclassified | 4 | ND | 10 | 25281 | 25 |
| Metallic foodware | 17 | ND | 26 | 1253 | 24 |
| Paints | 32 | ND | ND | 41801 | 16 |
| Ceramic foodware | 15 | ND | 35 | 1159 | 13 |
| Cosmetics | 38 | ND | ND | 42350 | 13 |
| Toys | 36 | ND | ND | 2123 | 6 |
| Staple food | 49 | ND | ND | 17 | 2 |
| Herbal/Trad Medicines | 2 | ND | ND | ND | 0 |
| Paint - craft/art | 3 | ND | ND | ND | 0 |
| Plastic foodware | 14 | ND | ND | ND | 0 |
| Spices | 55 | ND | ND | 2 | 0 |

ND = “non-detect” (lead concentration was below the XRF’s lower detection limit)

Percentage of Samples from the Philippines Below and Above the Reference Level


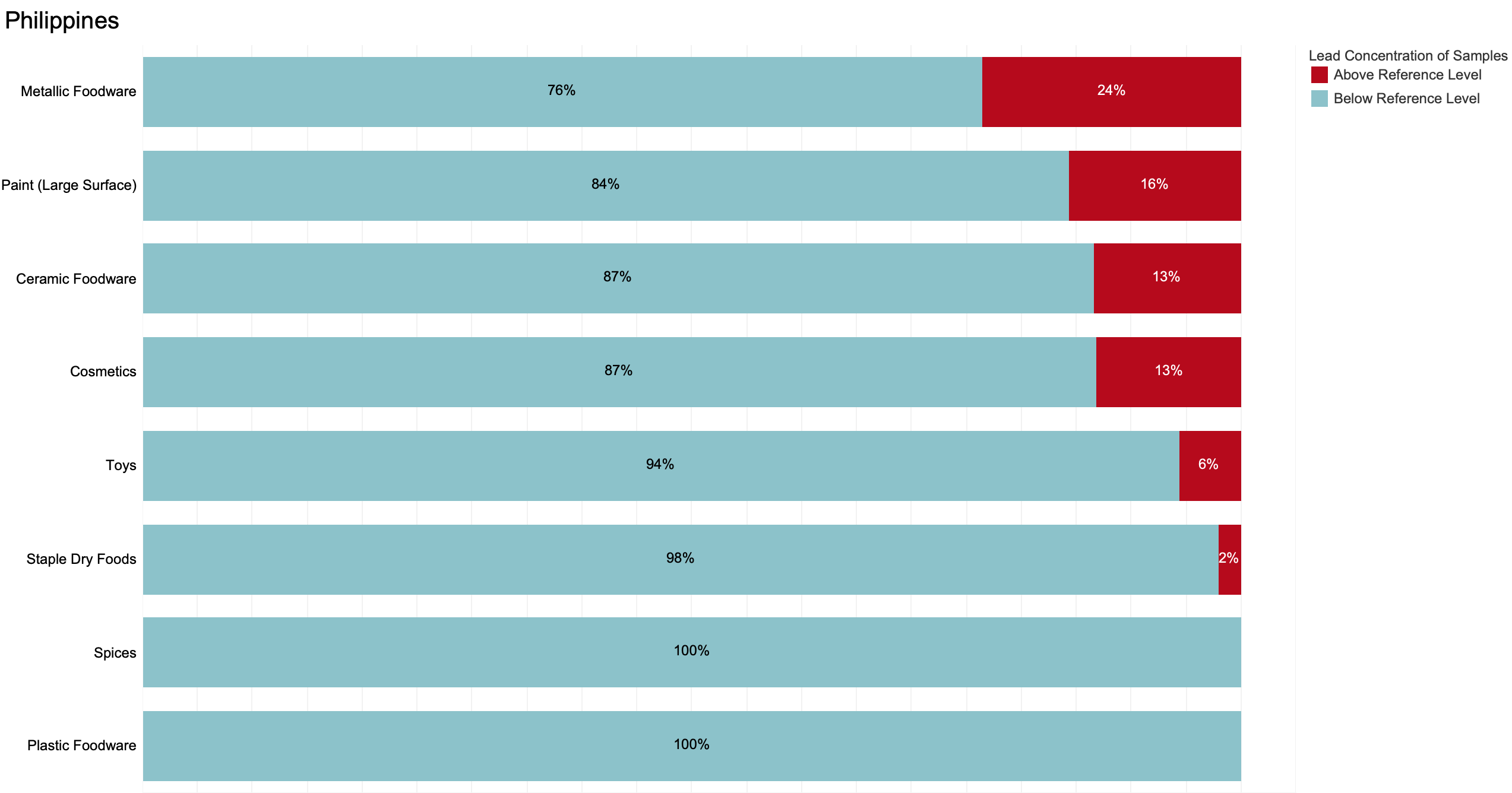


**Key:** Blue = percentage of samples below reference level. Red = percentage above reference level.

**Note**: Sample sizes below 5 are not displayed.

## Tajikistan

This report includes lead concentrations found in 20 samples from Tajikistan and of these, 30% exceeded the relevant reference levels. Readers should note that Pure Earth collected and conducted field XRF analyses of 191 samples from Tajikistan in total, however, the field XRF results were expunged after confirmatory testing of a subset of samples sent to New York suggested that the field XRF analyzer used in Tajikistan did not provide sufficiently accurate readings. This is the same XRF that was used in Kazakhstan, where field results were also expunged. This issue is discussed more fully in the Quality Control section. The results presented below represent only those results from the subset of samples sent to New York that were subjected to confirmatory testing by the New York-based XRF and an accredited lab.

Summary of Results from Tajikistan in Order of % Exceeding Reference Levels

| **Item Category** | **# of Samples** | **Min Value (ppm)** | **Median (ppm)** | **Max Value (ppm)** | **% Above Reference** |
| --- | --- | --- | --- | --- | --- |
| Ceramic foodware | 3 | 100700 | 133400 | 266000 | 100 |
| Spices | 5 | ND | 9 | 381 | 60 |
| Cosmetics | 4 | ND | ND | ND | 0 |
| Staple dry food | 3 | ND | ND | ND | 0 |
| Toys | 5 | ND | ND | 34 | 0 |

ND = “non-detect” (lead concentration was below the XRF’s lower detection limit)

Percentage of Samples from Tajikistan Below and Above the Reference Level


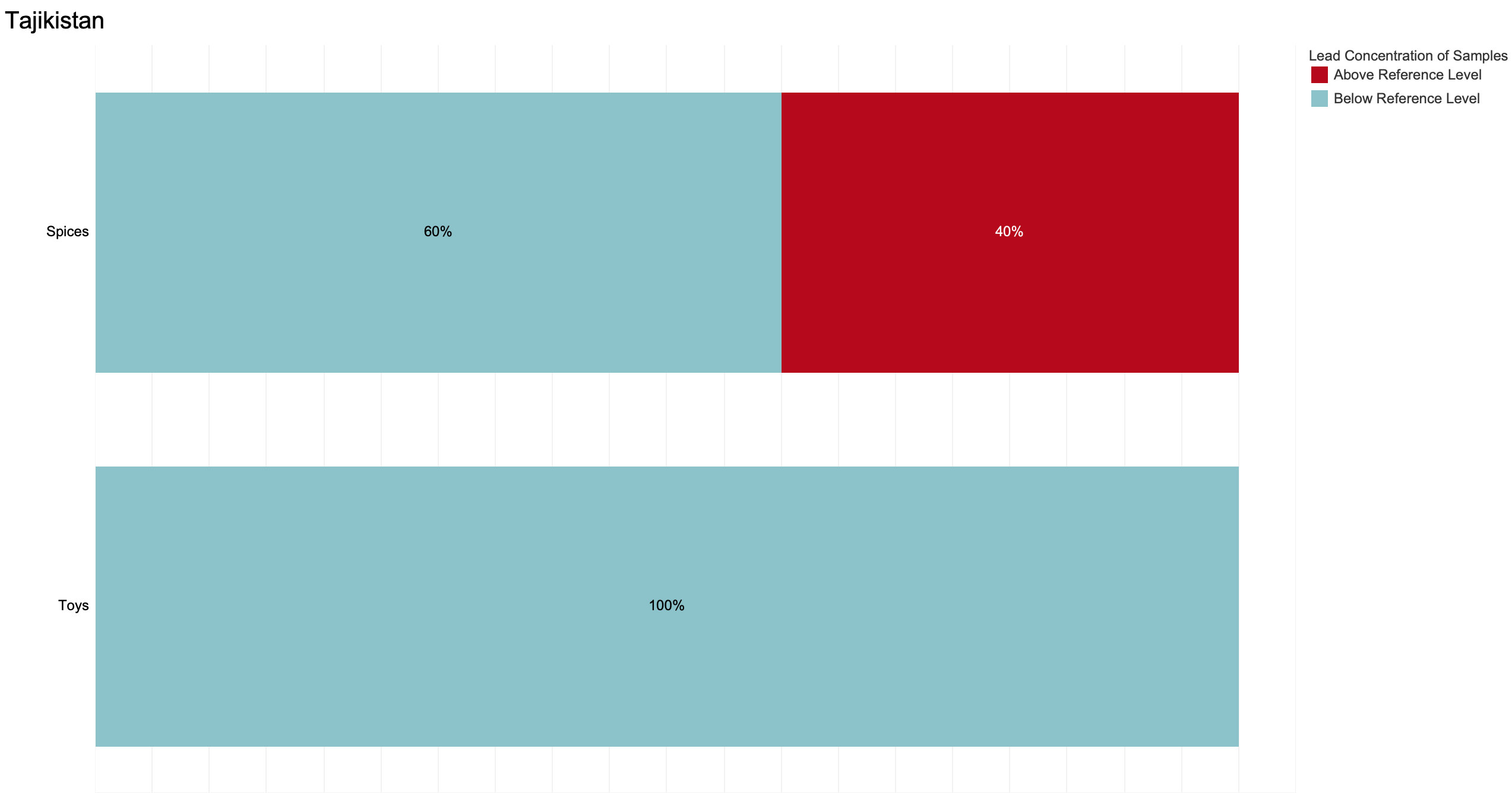


**Key:** Blue = percentage of samples below reference level. Red = percentage above reference level.

**Note**: Sample sizes below 5 are not displayed.

## Tanzania

Pure Earth analyzed a total of 212 samples from Tanzania, and of these, 10% exceeded the relevant reference levels. As with many countries, samples of foodware most commonly exceeded the relevant reference levels.

Summary of Results from Tanzania in Order of % Exceeding Reference Levels

| **Item Category** | **# of Samples** | **Min Value (ppm)** | **Median (ppm)** | **Max Value (ppm)** | **% Above Reference** |
| --- | --- | --- | --- | --- | --- |
| Paint - unclassified | 2 | 2139 | 3446 | 4752 | 100 |
| Ceramic foodware | 3 | 20 | 7780 | 22300 | 67 |
| Metallic foodware | 17 | ND | 30 | 15100 | 35 |
| Plastic foodware | 18 | ND | ND | 2791 | 17 |
| Toys | 30 | ND | ND | 698 | 10 |
| Paints - large surfaces | 28 | ND | ND | 866 | 7 |
| Cosmetics | 30 | ND | ND | 52 | 3 |
| Sweets | 30 | ND | ND | 4 | 3 |
| Spices | 45 | ND | ND | 21 | 2 |
| Staple dry food | 9 | ND | ND | ND | 0 |

ND = “non-detect” (lead concentration was below the XRF’s lower detection limit)

**Note:** The category of Paint (unclassified) includes only two samples.

Percentage of Samples from Tanzania Below and Above the Reference Level


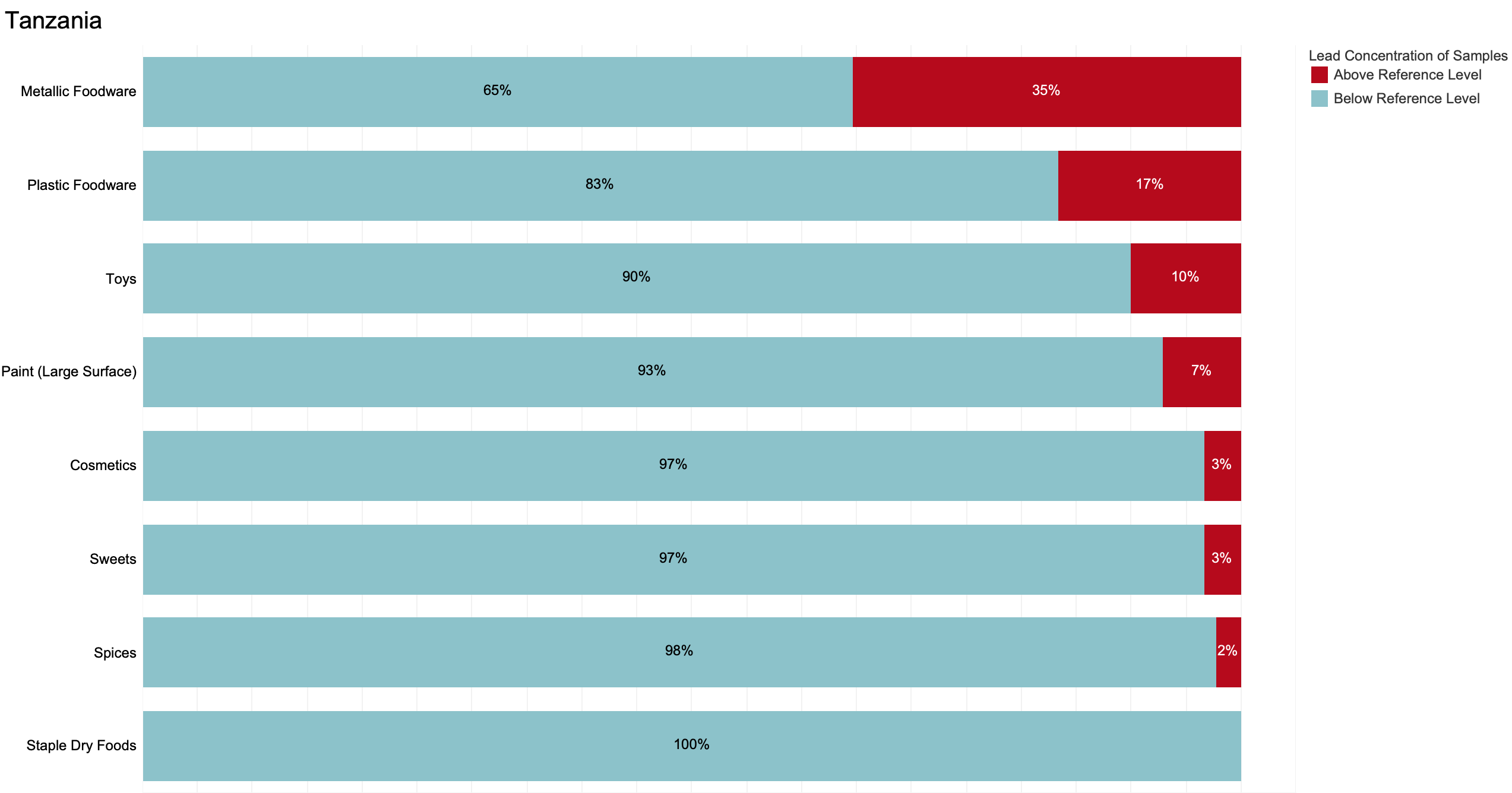


**Key:** Blue = percentage of samples below reference level. Red = percentage above reference level.

**Note**: Sample sizes below 5 are not displayed.

## Tunisia

Pure Earth analyzed 190 samples from Tunisia, and of these, 13% exceeded the relevant reference levels. Samples of ceramic foodware and paint most commonly exceeded the relevant reference levels.

Summary of Results from Tunisia in Order of % Exceeding Reference Levels

| **Item Category** | **# of Samples** | **Min Value (ppm)** | **Median (ppm)** | **Max Value (ppm)** | **% Above Reference** |
| --- | --- | --- | --- | --- | --- |
| Ceramic foodware | 16 | ND | 251 | 68600 | 56 |
| Paints - large surfaces | 14 | ND | 286 | 72000 | 50 |
| Herbal/Trad Medicines | 6 | ND | ND | 19 | 17 |
| Metallic foodware | 25 | ND | ND | 26600 | 12 |
| Cosmetics | 27 | ND | ND | 712 | 11 |
| Plastic foodware | 23 | ND | ND | 3289 | 4 |
| Toys | 25 | ND | ND | 176 | 4 |
| Spices | 36 | ND | ND | ND | 0 |
| Staple dry food | 18 | ND | ND | ND | 0 |

ND = “non-detect” (lead concentration was below the XRF’s lower detection limit)

Percentage of Samples from Tunisia Below and Above the Reference Level


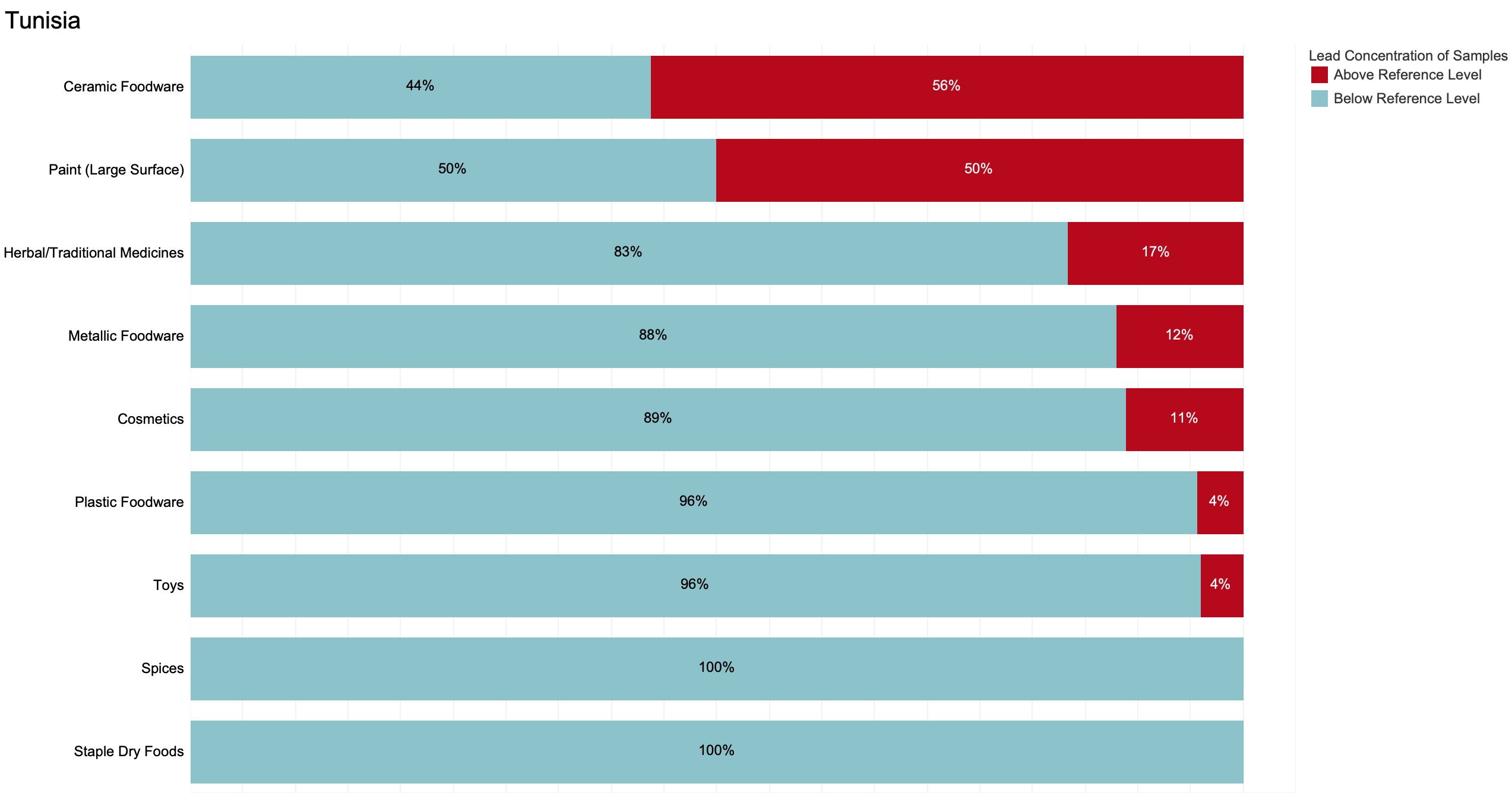


**Key:** Blue = percentage of samples below reference level. Red = percentage above reference level.

**Note**: Sample sizes below 5 are not displayed.

## Turkey

Pure Earth analyzed a total of 104 samples from Turkey, and of these, 38% exceeded the relevant reference levels. Compared to other countries, a high percentage of samples from a wide variety of categories exceeded relevant reference levels.

Summary of Results from Turkey in Order of % Exceeding Reference Levels

| **Item Category** | **# of Samples** | **Min Value (ppm)** | **Median (ppm)** | **Max Value (ppm)** | **% Above Reference** |
| --- | --- | --- | --- | --- | --- |
| Cosmetics | 1 | 121 | 121 | 121 | 100 |
| Paints - large surfaces | 10 | ND | 3937 | 11200 | 70 |
| Metallic foodware | 9 | ND | 171 | 903 | 67 |
| Ceramic foodware | 15 | ND | 119 | 14300 | 53 |
| Toys | 49 | ND | 22 | 4336 | 29 |
| Spices | 4 | ND | ND | 4 | 25 |
| Plastic foodware | 16 | ND | ND | 1281 | 19 |

ND = “non-detect” (lead concentration was below the XRF’s lower detection limit)

Percentage of Samples from Turkey Below and Above the Reference Level


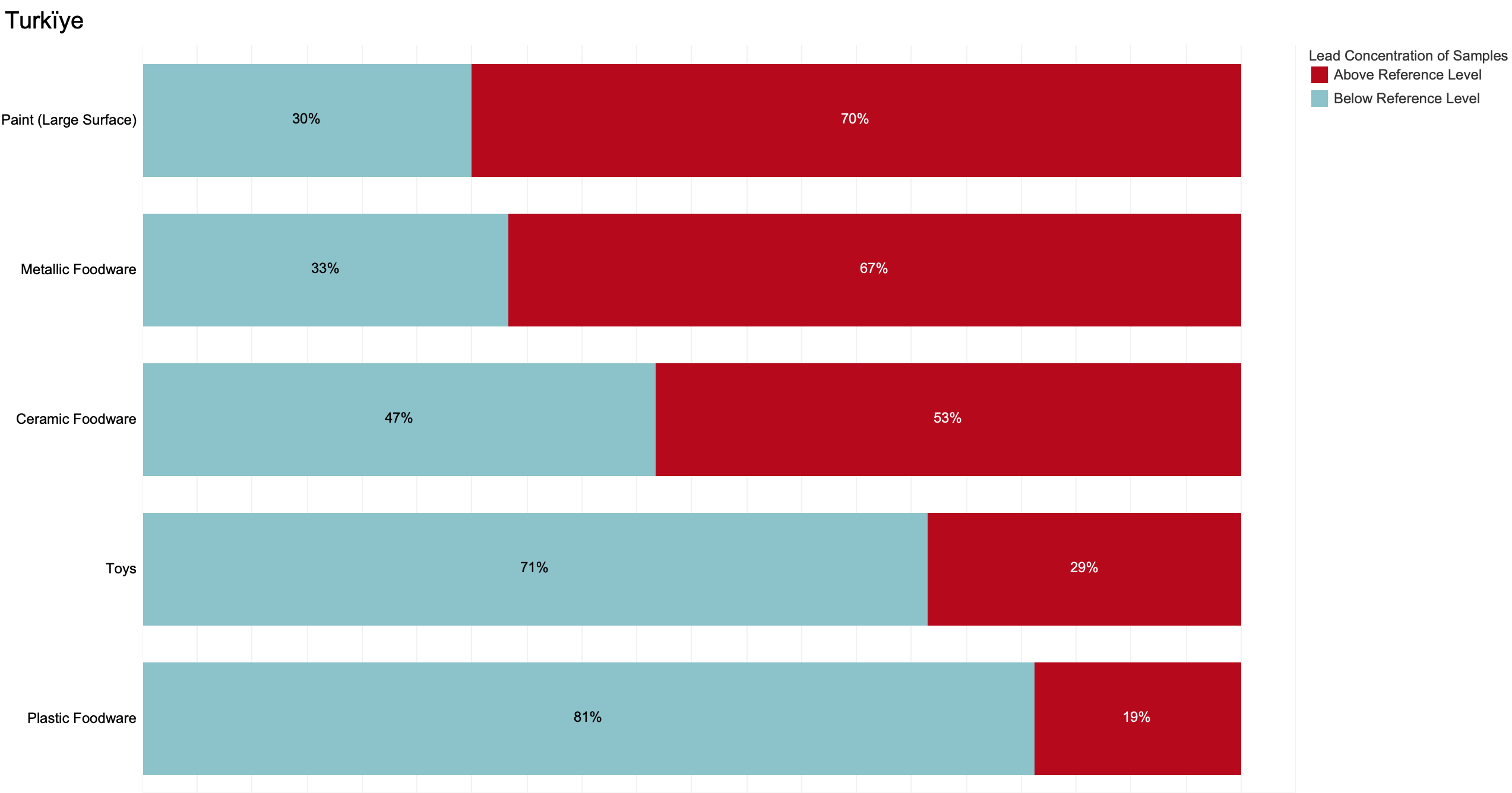


**Key:** Blue = percentage of samples below reference level. Red = percentage above reference level.

**Note**: Sample sizes below 5 are not displayed.

## Uganda

Pure Earth analyzed 224 samples from Uganda, and of these, 12% exceeded the relevant reference levels. Note that only one sample of medicine was analyzed, and thus the 100% of medicine samples exceeding the reference level should be viewed in that context. As with other countries, metallic foodware commonly exceeded the relevant reference level.

Summary of Results from Uganda in Ordered of % Exceeding Reference Levels

| **Item Category** | **# of Samples** | **Min Value (ppm)** | **Median (ppm)** | **Max Value (ppm)** | **% Above Reference** |
| --- | --- | --- | --- | --- | --- |
| Herbal/Trad Medicines | 1 | 31 | 31 | 31 | 100 |
| Metallic foodware | 15 | ND | 303 | 1564 | 73 |
| Plastic foodware | 30 | ND | ND | 1032 | 20 |
| Paints - large surfaces | 32 | ND | ND | 12600 | 16 |
| Ceramic foodware | 12 | 17 | 23 | 6092 | 8 |
| Staple dry food | 17 | ND | ND | 3 | 6 |
| Cosmetics | 48 | ND | ND | 3 | 2 |
| Spices | 40 | ND | ND | ND | 0 |
| Toys | 29 | ND | ND | 81 | 0 |

ND = “non-detect” (lead concentration was below the XRF’s lower detection limit)

Percentage of Samples from Uganda Below and Above the Reference Level


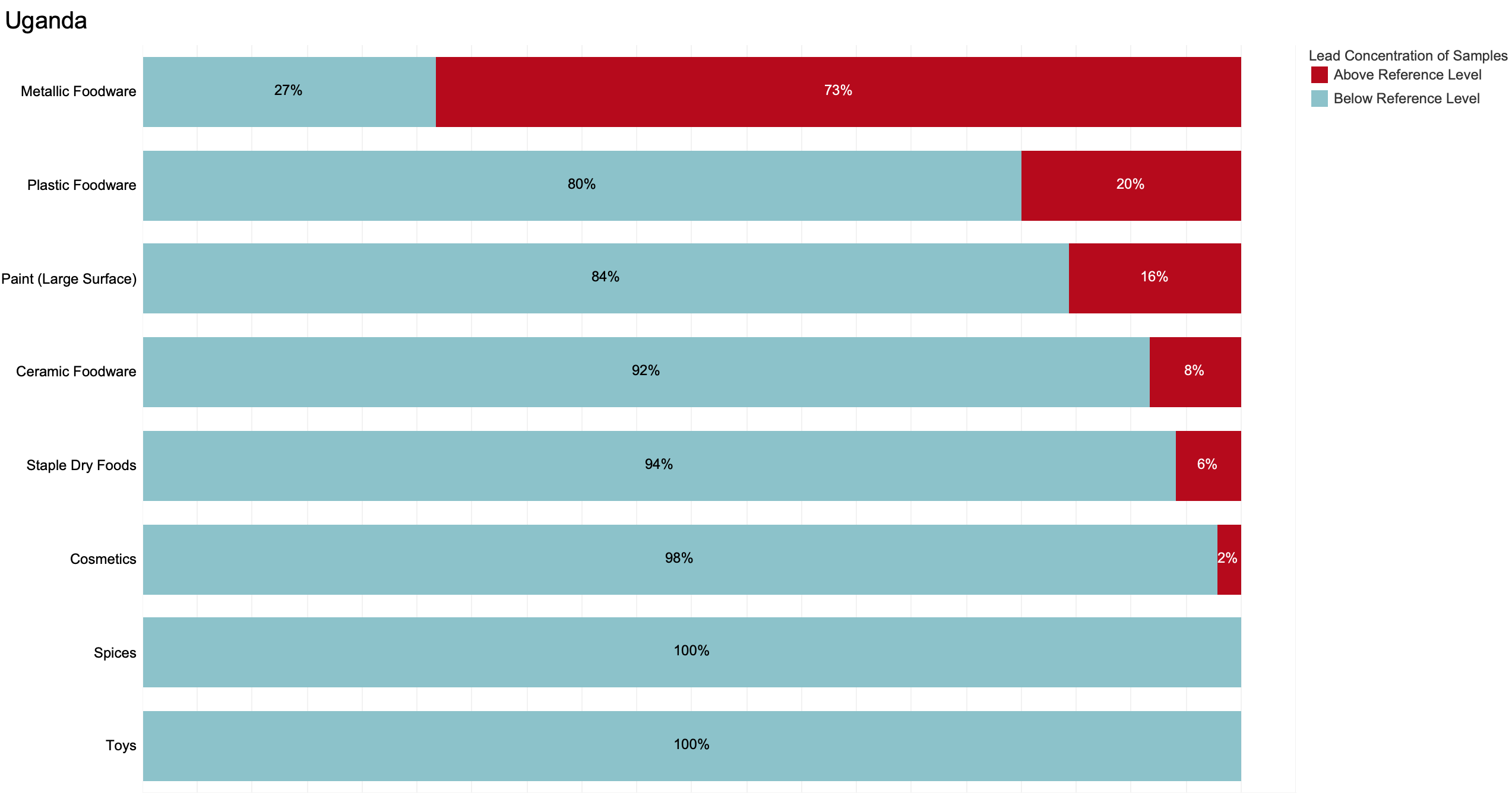


**Key:** Blue = percentage of samples below reference level. Red = percentage above reference level.

**Note**: Sample sizes below 5 are not displayed.

## Vietnam

Pure Earth analyzed a total of 175 samples from Vietnam, and of these, 22% exceeded the relevant reference levels. As with other countries, metallic foodware, ceramic foodware, and paints most commonly exceeded the relevant reference levels.

Summary of Results from Vietnam Ordered by % Exceeding Reference Levels

| **Item Category** | **# of Samples** | **Min Value (ppm)** | **Median (ppm)** | **Max Value (ppm)** | **% Above Reference** |
| --- | --- | --- | --- | --- | --- |
| Paints - large surfaces | 17 | ND | 777 | 25505 | 59 |
| Metallic foodware | 18 | ND | 269 | 13080 | 56 |
| Paint - unclassified | 2 | ND | 552 | 1104 | 50 |
| Paint - craft/art | 6 | ND | 612 | 7296 | 50 |
| Ceramic foodware | 14 | ND | 59 | 19789 | 29 |
| Cosmetics | 30 | ND | ND | 68 | 23 |
| Toys | 30 | ND | ND | 298 | 7 |
| Spices | 31 | ND | ND | 9 | 3 |
| Herbal/Trad Medicines | 5 | ND | ND | ND | 0 |
| Plastic foodware | 13 | ND | ND | 9 | 0 |
| Staple dry food | 9 | ND | ND | ND | 0 |

ND = “non-detect” (lead concentration was below the XRF’s lower detection limit)

Percentage of Samples from Vietnam Below and Above the Reference Level


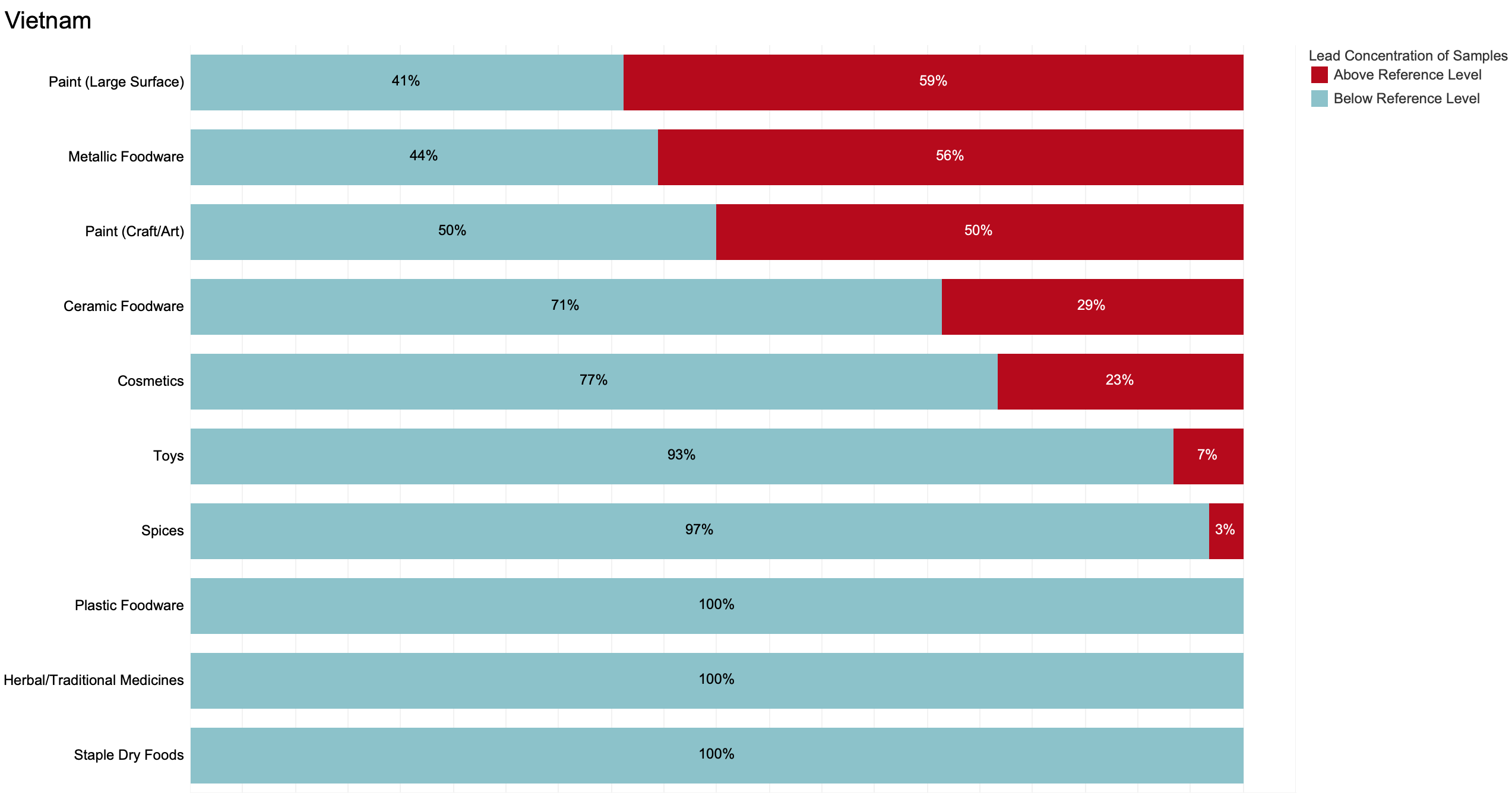


**Key:** Blue = percentage of samples below reference level. Red = percentage above reference level.

**Note**: Sample sizes below 5 are not displayed.

1. Weidenhamer JD, Kobunski PA, Kuepouo G, Corbin RW, Gottesfeld P. Lead exposure from aluminum cookware in Cameroon. Sci Total Environ. 2014 Oct 15;496:339-347. [↑](#footnote-ref-1)
2. US EPA SW-846 Method 6020 for lead (inductively coupled plasma-mass spectrometry or ICP-MS) and SW-846 Method 6010C for aluminum (inductively coupled plasma-atomic emission spectrometry or ICP-AES) [↑](#footnote-ref-2)
3. INUK computations by Dr. Jack Caravanos, DrPH, CIH, Clinical Professor, NYU [↑](#footnote-ref-3)
4. Hore P, Alex-Oni K, Sedlar S, Nagin D. A Spoonful of Lead: A 10-Year Look at Spices as a Potential Source of Lead Exposure. J Public Health Manag Pract. 2019 Jan/Feb;25 Suppl 1, Lead Poisoning Prevention:S63-S70. doi: 10.1097/PHH.0000000000000876. PMID: 30507772. [↑](#footnote-ref-4)
5. Forsyth, Jenna E., et al. "Sources of blood lead exposure in rural Bangladesh." Environmental science & technology 53.19 (2019): 11429-11436. [↑](#footnote-ref-5)
6. Forsyth, Jenna E., et al. "Food safety policy enforcement and associated actions reduce turmeric lead chromate adulteration across Bangladesh." *Environmental Research* (2023): 116328. [↑](#footnote-ref-6)
7. Ericson, Bret, et al. "Elevated levels of lead (Pb) identified in Georgian spices." *Annals of Global Health* 86.1 (2020). [↑](#footnote-ref-7)
